# In Situ Lipoprotein‐seeking Dye for in Vivo Real‐Time Imaging of Lipid Dysregulation Diseases

**DOI:** 10.1002/advs.202514290

**Published:** 2026-01-27

**Authors:** Yijing Du, Zetao Dang, Baofeng Xu, Jiajun Xu, Dan Wang, Yuewei Zhang, Shoujun Zhu

**Affiliations:** ^1^ Joint Laboratory of Opto‐Functional Theranostics in Medicine and Chemistry First Hospital of Jilin University Changchun P.R. China; ^2^ State Key Laboratory of Supramolecular Structure and Materials Center For Supramolecular Chemical Biology College of Chemistry Jilin University Changchun P.R. China; ^3^ Changbaishan Laboratory Jilin University Changchun P.R. China; ^4^ Stroke Center Department of Neurology First Hospital of Jilin University Changchun P.R. China; ^5^ Key Laboratory of Chemical Biology of Hebei Province College of Chemistry and Materials Science Key Laboratory of Medicinal Chemistry and Molecular Diagnosis of the Ministry of Education Hebei University Baoding P.R. China

**Keywords:** fluorescent probes, LDL, lipid disorders, Near‐infrared‐II imaging, natural lipoproteins

## Abstract

Lipoproteins, critical transporters of cholesterol and triglycerides, are essential to cardiovascular health and pathology. However, imaging probes that can specifically target endogenous lipoproteins in situ are lacking. This study introduces an innovative lipoprotein‐seeking near‐infrared‐II (NIR‐II) dye for in vivo imaging of lipid dysregulation diseases independent of immune interactions. This dye demonstrates high affinity and specificity for lipoproteins, enabling in situ selective lipoprotein‐seeking within the body, unaffected by other proteins. The lipoprotein‐seeking dynamics can be precisely modulated through rationally tuning hydrophilic moieties of dye structure. The lipoprotein‐seeking dye enables the real‐time high‐contrast detecting subtle biological changes associated with lipid metabolism disorders, successfully delineating the presence of fatty deposits in hepatic tissues and identifying the early formation of atherosclerotic plaques in cardiovascular systems. Notably, our dye can selectively image low density lipoprotein (LDL) without highlighting high density lipoprotein (HDL) through appropriate irradiation. The NIR‐II dye's ability to target lipoproteins and provide clear imaging could revolutionize the management of lipoprotein‐related conditions, facilitating earlier interventions and more personalized treatment strategies.

## Graphical abstract

This study develops lipoprotein‐seeking NIR‐II dyes that specifically bind circulating lipoproteins. These dyes form ultra‐stable complexes with endogenous lipoprotein, enabling real‐time, high‐contrast imaging of fatty liver and atherosclerotic plaques. Tunable binding kinetics allow customized imaging windows. This technology offers early diagnosis and monitoring of lipid‐related diseases with high specificity.

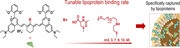

## 1 Introduction

Chronic inflammation coupled with abnormal lipid and lipoprotein metabolism is a fundamental cause of metabolic syndrome‐related diseases, including atherosclerosis, nonalcoholic fatty liver disease (NAFLD), and cardiovascular disease (CVD) [1, 2, 3, 4]. Current imaging modalities for the detection and characterization of fatty liver disease and atherosclerotic plaques, such as ultrasound, computed tomography (CT), and magnetic resonance imaging (MRI) [5, 6, 7, 8, 9], provide valuable insights yet exhibit limitations in sensitivity and specificity, particularly in the early stages of disease. Imaging analysis of test results also requires sufficient relevant experience. Moreover, the use of ionizing radiation in some of these techniques poses additional risks to patients/animal models. Therefore, there is an urgent need for the development of novel imaging agents that can safely, specifically, directly, and non‐invasively illuminate these lipid‐rich pathologies, particular for lab‐scale small animal imaging.

Near‐infrared‐II (NIR‐II) imaging has emerged as a revolutionary technology owing to its exceptional tissue penetration and minimal autofluorescence, enabling high‐resolution visualization of deep tissue structures [10, 11, 12, 13, 14, 15, 16, 17, 18, 19, 20, 21, 22]. Recent advancements of NIR‐II probes have successfully targeted atherosclerotic plaques through antibody conjugation [23, 24, 25]. These probes have proven capable of detecting atherosclerotic plaques in animal models. However, antibody‐based contrast agents suffer from instability in bodily fluids and accumulation by the immune system, leading to strong off‐target signals and low imaging signal‐to‐noise ratios.

As the primary lipid transport carriers, lipoproteins are directly involved in numerous physiological and pathological processes. The hydrophilic phospholipid monolayer and hydrophobic core of lipoproteins make them excellent carriers for hydrophobic drugs and imaging agents [26, 27, 28, 29]. The diverse apolipoprotein species on the surface of lipoproteins enable their specific recognition by various lipoprotein receptors, which have been identified in numerous tissues and organs, particularly showing overexpression in certain cancer or non‐neoplastic diseases [30]. Currently, most studies on lipoproteins for drug delivery and contrast agent primarily involves the reconstitution of purified lipoproteins isolated from human plasma with drugs (recombinant lipoproteins) or the use of synthetic nanoparticles resembling the structure of lipoproteins (synthetic lipoproteins) [31, 32, 33, 34, 35, 36, 37, 38]. These methods are constrained by the challenges of lipoprotein isolation and purification, as well as the modification of the entire lipoprotein particle or extraction of specific lipid or protein components. The intricate production processes and substantial costs have impeded their industrial advancement and clinical application. Previous work by Couvreur demonstrated that squalene‐gemcitabine or squalene‐rhodamine conjugate nanoparticles associate with endogenous lipoproteins [27, 39, 40], encouraging us to explore the potential for NIR‐II small molecules to selective incorporation into endogenous lipoproteins and produce fluorescence enhancement. By utilizing their association with lipoproteins rather than relying on antigen‐antibody interactions, these dyes can reveal lipoprotein metabolism disorders and visualize tissues with aberrant lipoprotein targeting and lipid accumulation, such as atherosclerotic plaques and fatty liver.

**FIGURE 1:**
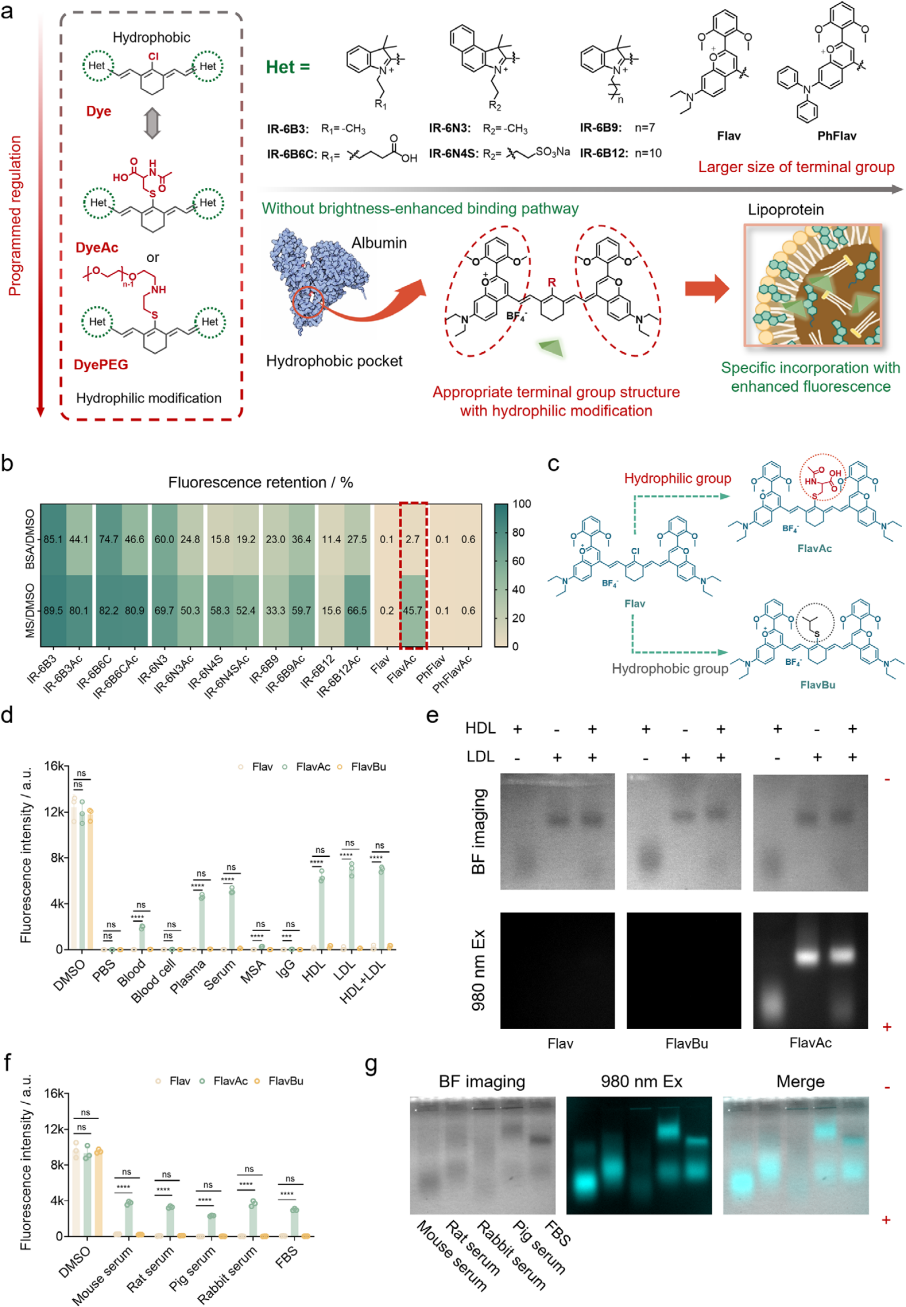
Screening lipoprotein‐seeking dyes with specific NIR‐II fluorescence “on”. (a) Schematic diagram of molecular screening and design of lipoprotein‐seeking dyes with enhanced fluorescence signal. The abundance of albumin generally results in non‐specific fluorescence signal in living organisms whereas the design of lipoprotein‐seeking dyes ensures specific incorporation into lipoprotein. The illustration of the lipoprotein was created in BioRender. (b) Fluorescence retention of typical cyanine dyes and corresponding dyes modified by acetylcysteine incubated in BSA solution (50 mg mL^−1^) and mice serum (MS) at 50°C for 2 h (n = 3). Fluorescence retention is defined as the fluorescence intensity of the dye in a given solution relative to that in DMSO. (c) Chemical structure of FlavAc and FlavBu. (d) Fluorescence intensity of Flav, FlavAc, and FlavBu after incubating with DMSO, PBS, and different blood components (n = 3). MSA: mouse serum albumin; IgG: immunoglobulin G; HDL: high‐density lipoprotein; LDL: low‐density lipoprotein; exposure time: 1 ms. (e) Agarose gel electrophoresis of FlavAc in separate HDL, LDL, and equal volume mixture solution of HDL and LDL. 980 nm Ex: excited by 980 nm laser; BF imaging: bright field imaging; exposure time: 5 ms. (f) Fluorescence intensity of Flav, FlavAc, and FlavBu after incubating with serum from different species (n = 3). FBS: fetal bovine serum; exposure time: 0.8 ms. (g) Agarose gel electrophoresis of FlavAc in serum of different species. Exposure time: 5 ms; over 1100 nm; power density: 65 mW cm^−2^. Significance was defined as *p < 0.05, **p < 0.01, ***p < 0.001, ****p < 0.0001.

Herein, we introduce an innovative lipoprotein‐seeking NIR‐II dye with a high affinity and specificity for lipoproteins, thereby enabling the precise imaging of fatty liver and atherosclerotic plaques. The dye's unique chemical structure allows for its selective incorporation into lipoproteins with great brightness enhancing, but not other interferential proteins like albumin, offering a new window into the pathophysiology of lipid disorders at a molecular level (Figure 1a). Lipoproteins effectively enhance the fluorescence brightness and photostability of the dye by forming exceptionally stable fluorescent complexes (with 128‐fold enhanced brightness) under physiological conditions for precise in situ imaging. By adjusting the size of hydrophilic segment, the binding kinetics between the dye and lipoproteins can be regulated, allowing customized imaging time windows (from minutes to hours) for spatiotemporal visualization of lipoprotein dynamics. The lipoprotein‐seeking dye can incorporate into either high‐density lipoprotein (HDL) or low‐density lipoprotein (LDL), with a stronger affinity particularly for LDL, and through laser irradiation, it selectively illuminates LDL. By combining the advantage of NIR‐II fluorescence imaging, this dye facilitates the early detection, accurate diagnosis, and effective monitoring of fatty liver disease and atherosclerotic progression. Furthermore, this strategy outlines an approach for creating contrast agents that guide therapeutic interventions and monitor treatment efficacy, thereby playing an important role in the clinical management of patients.

## 2 Results and Discussion

### 2.1 Avoiding Albumin Capture of Cyanine Dyes as a Prerequisite for Screening Potential Lipoprotein‐Seeking Dyes

Albumin is one of the most abundant endogenous macromolecules and a versatile drug carrier [41]. A variety of cyanine dyes have a strong affinity for albumin, and the appropriately sized hydrophobic dyes can be captured by the hydrophobic cavity of albumin to produce fluorescence enhancement [42]. However, this nonspecific fluorescence enhancement is not always advantageous. For instance, while ICG‐containing lipid nanoparticles (LNPs) remain stable in phosphate‐buffered saline (PBS), upon intravenous administration, ICG rapidly leaks from the LNPs due to its high affinity for serum albumin [43]. Therefore, developing lipoprotein‐seeking dyes to minimize nonspecific albumin binding (especially the brightness‐enhanced binding pathway) is crucial [44, 45]. Based on the structural characteristics of albumin and lipoproteins, as well as previous protein binding experiences [46, 47, 48], we first established a targeted cyanine dyes library. These dyes featured the same cyclohexene linker with a Cl‐ substituent as a potential site for covalent binding to proteins (through nucleophilic substitution by cysteine thiol groups), while an acetylcysteine group (Ac‐) was introduced to prevent covalent bond formation with proteins and to enhance hydrophilicity, thereby reducing the likelihood of being trapped in hydrophobic protein pockets (Figure S1). We hypothesize that the different size and hydrophobic backbones of cyanine dyes may exhibit varying binding affinities for albumin and lipoproteins.

All dyes were incubated separately with mouse serum (MS containing both albumin and lipoproteins) and albumin (∼50 mg mL^−1^) at 50°C for 2 h. Subsequently, the fluorescence intensity of the reaction mixture was assessed, followed by sample separation via gel electrophoresis (Figure 1b; Figure S2). The corresponding NIR‐II brightness was compared with dyes in dimethyl sulfoxide (DMSO) and PBS, aiming to quantify the protein‐binding ability of dyes (a higher NIR‐II fluorescence retention indicates a greater brightness‐enhanced protein targeting pathway). The results showed that most of dyes containing indole and benzoindole rings exhibited fluorescence retention in both albumin solutions and MS. Although the brightness of the dyes decreased in both albumin and serum with increasing steric hindrance from the side chains, the albumin‐binding ability is dominant. In contrast, Flav and PhFlav, which have larger, more rigid terminal groups, exhibited very low fluorescence retention in both albumin and MS, indicating that they were unable to enter the hydrophobic pocket of albumin and lipoprotein. Notably, FlavAc exhibited considerable fluorescence retention only in serum, but not in albumin and PBS, suggesting its potential interaction with other blood components.

### 2.2 Validation of Specificity and Universality in Dye Incorporation Into Native Lipoproteins

To verify the targeted mechanism of FlavAc in serum, we modified the Flav dye with hydrophilic and hydrophobic groups separately to alter the hydrophilic distribution of the molecule, with minimal change to the dye's peak wavelength of absorption and fluorescence in DMSO. With increasing hydrophobicity, FlavBu exhibited more obvious H‐aggregation behavior in PBS (Figure 1c; Figure S3). Subsequently, all three dyes were separately co‐incubated with DMSO and various blood components, revealing that only FlavAc exhibited bright NIR‐II signals in blood, plasma, serum, and lipoproteins (Figure 1d). The results demonstrated that the interaction between blood lipoproteins and FlavAc is responsible for the observed enhancement of the fluorescence signal. Then, the mixtures of dyes incubated with different human lipoproteins were conducted by agarose gel electrophoresis (Figure 1e). NIR‐II fluorescence was observed in the lipoprotein bands marked by Sudan Black B only after the addition of FlavAc. No NIR‐II intensity was detected in the gels containing Flav or FlavBu. To validate the universality of this phenomenon in serum, we incubated FlavAc with serum from different species labeled by Sudan Black B and separated the lipoproteins using agarose gel electrophoresis (Figure 1f,g; Figure S4). The results indicated that FlavAc exhibited nearly identical labeling specificity for lipoprotein across different serum in accordance with Sudan Black B. Specific NIR‐II fluorescence enhancement was observed in corresponding bands, suggesting that FlavAc effectively excluded interference from abundant albumin and other components in blood, forming stable complexes with lipoproteins while maintaining bright NIR‐II intensity. In addition, we also selected representative albumin‐binding dyes, including IR‐6B6C/IR‐6B6CAc/IR‐6N4S/IR‐6N4SAc, for the same incubation with serum and separation verification (Figure S5). The results showed that fluorescence signal of IR‐6B6C/IR‐6B6CAc barely overlapped with lipoprotein bands, but with the strong interaction between dyes and albumin. Although IR‐6N4S/IR‐6N4SAc could label certain lipoprotein bands, their strong interactions with albumin demonstrated that they lack specificity for lipoprotein labeling (Figure S2).

The binding affinity of FlavAc for various blood components (HDL, LDL, and HSA) was measured by Microscale Thermophoresis (MST).​ The results showed no detectable interaction between FlavAc and HDL, whereas FlavAc exhibited strong binding affinity toward both LDL and albumin (Figure S6). Notably, although there is detectable binding affinity between FlavAc and albumin, FlavAc produced almost no fluorescence enhancement in albumin solution (perheps from non‐brightness‐enhanced binding pathway). We speculated that although the dye might facilitate hydrogen bonding or other non‐covalent interactions with the surface of albumin, this interaction did not alter the aggregation‐caused quenching behavior of the dye in the aqueous environment. Thus, we concluded that the fluorescence signal of FlavAc in blood was still primarily due to lipoprotein‐specific enhancement, with LDL serving as the major contributor. Moreover, the MST results also revealed that Flav had a weaker affinity for LDL compared to FlavAc (Figure S6). Due to the excessively large molecular weight of lipoproteins, which is not ideal for most binding affinity tests, combined with the inherent variability and limitations of MST, the data herein serves primarily as a qualitative reference.

### 2.3 Characterization of NIR‐II Dye@lipoprotein Complexes

**FIGURE 2:**
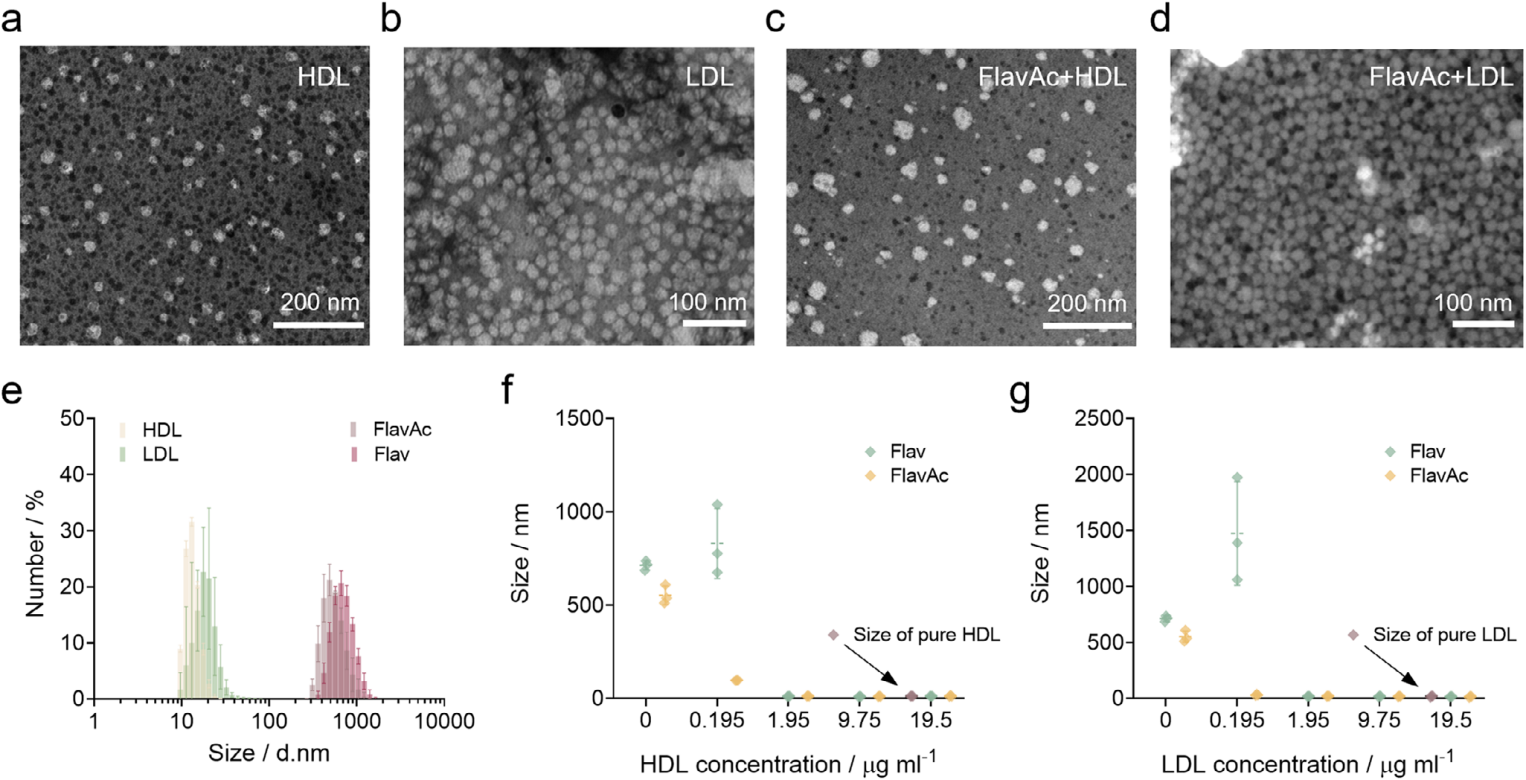
Characterization of FlavAc@lipoprotein. (a–d) TEM images of HDL, LDL, FlavAc@HDL, and FlavAc@LDL. Visualized by phosphotungstic acid negative staining. (e) Particle size distribution of HDL, LDL, Flav, and FlavAc measured by DLS (n = 3). The concentrations of HDL and LDL were respectively 1.95 µg mL^−1^ and 2.4 µg mL^−1^. The concentrations of Flav and FlavAc were 1 µM. (f,g) Particle size changes with added lipoprotein (HDL and LDL) concentration (n = 3). The concentrations of Flav and FlavAc were always 1 µM.

Transmission electron microscopy (TEM) was used to characterize lipoproteins and the FlavAc@lipoprotein complex, further confirming the impact of dye addition on lipoprotein morphology (Figure 2a–d). HDL experienced a partial increase in particle size before and after co‐incubation with FlavAc, while the particle size of LDL did not change obviously and morphology remained spherical before and after co‐incubation with FlavAc. The mean hydration diameters of both single dyes and lipoproteins were determined using dynamic light scattering (DLS) (Figure 2e). Due to their large hydrophobic conjugated structures, both Flav and FlavAc aggregated into larger particles in aqueous solution which resulted in fluorescence quenching. The particle sizes of purified lipoproteins were approximately 13 nm (HDL) and 19 nm (LDL) with a polydispersity index (PDI) ranging from 0.3 to 0.4. The ideal size enabled long circulation by protecting the particles from rapid renal clearance and preventing recognition by the reticuloendothelial system, thereby making the particles potential natural carriers for drugs and imaging agents.

To further validate the specific binding of lipoproteins with FlavAc, different concentrations of lipoprotein were incubated with certain concentrations of dyes to obtain average particle sizes of the incubated system (Figure 2f, g). After adding very low concentrations (∼0.2 µg mL^−1^) of HDL and LDL, the mean particle size in the FlavAc system rapidly decreased, whereas marginal size variation was observed in the Flav system. Statistical analysis of the PDI across different systems revealed a trend of initial increase followed by a decrease with increasing lipoprotein concentration (Figure S7). Notably, the PDI value in the FlavAc group returned to a level comparable to that of purified lipoproteins early. This suggested that the lipoproteins effectively reduced the number of FlavAc aggregates in aqueous solution, leading to the formation of targeted complexes. Absorption spectra for the three dyes were acquired in PBS following adding different concentrations of HDL or LDL (Figure S8), with the mixtures subsequently incubated at 50°C for 30 min to ensure complete binding. The results showed that as the lipoprotein concentration increased, a new absorption peak emerged around 1000 nm specifically for FlavAc, while no such peak was observed for either Flav or FlavBu. This also suggested that the addition of lipoproteins effectively modulated the aggregation behavior of FlavAc in aqueous solution.

### 2.4 In Vitro Binding Kinetics Between Dyes and Lipoprotein

**FIGURE 3:**
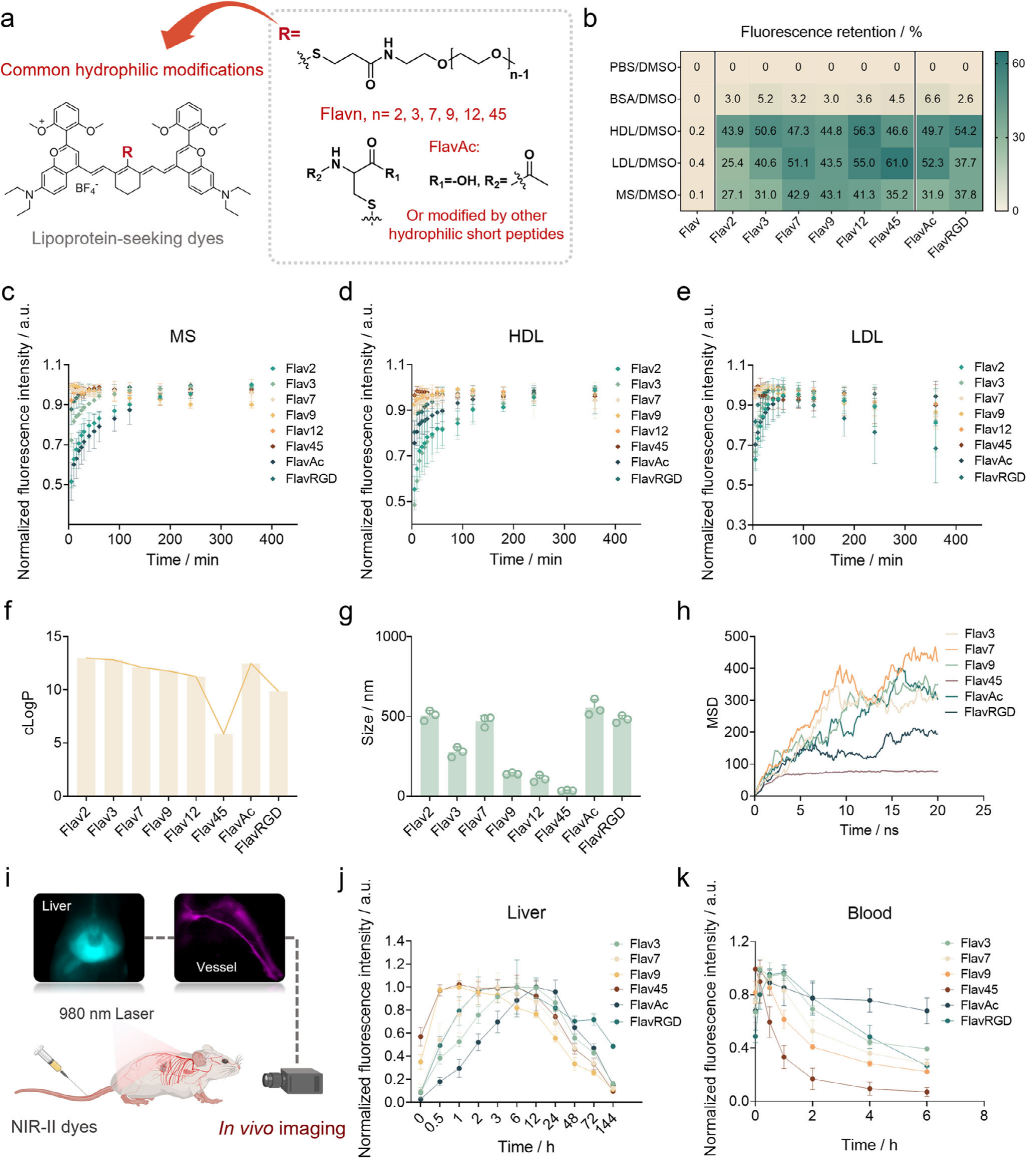
Kinetics of binding of dyes to lipoproteins and in situ lipoprotein‐seeking for in vivo imaging. (a) General chemical structure of mPEG with different molecular weights and amino acid or polypeptide modified cyanine dyes. (b) Fluorescence retention of various dyes (10 µM) incubated in MS, BSA (50 mg mL^−1^), HDL (1.95 mg mL^−1^), and LDL (2.4 mg mL^−1^) solution. Fluorescence retention is defined as the fluorescence intensity of the dye in a given solution relative to that in DMSO (100%). (c–e) Time‐dependent changes in the normalized fluorescence intensity of different dyes (10 µM) in MS (c), HDL (d), and LDL (e). Data were normalized to the maximum value within each group (n = 3). (f) The calculated LogP value of the molecules calculated by ChemDraw. (g) The average particle size of dyes formed in PBS (n = 3). Concentration: 1 µM. (h) Mean square displacement (MSD) of different dyes in simulation boxes with 20 ns. (i) NIR‐II imaging of mouse liver and hind limb vessels after intravenous injection of dyes. The illustration was created in BioRender. (j) Normalized fluorescence intensity of liver after tail vein injection of different dyes (n = 3). (k) Normalized fluorescence intensity of collected blood at different post‐injection time points of different dyes (n = 3). Data were normalized to the maximum value.

Based on the above study, the introduction of certain hydrophilic groups can effectively alter the binding behavior of Flav dyes with lipoproteins. To achieve the efficient incorporation of dyes into lipoproteins in vivo, it is essential to explore the relationship between molecular structure and binding kinetics with lipoproteins. We further synthesized lipoprotein‐seeking dyes variants by modifying Flav with varying lengths of PEG chains or short peptides to programmed modulation of the binding rate between the dye and lipoproteins (Figure 3a). These dyes exhibited similar absorption and emission peak wavelength in DMSO and PBS, indicating that the optical properties of the dyes were not altered by the substitution of the thiol group (Figures S9 and S10). The fluorescence retention of dyes in PBS, bovine serum albumin (BSA), HDL, LDL, and MS were measured following co‐incubation at 50°C for 2 h, using the fluorescence intensity in DMSO as reference (Figure 3b). The results indicated that even with the introduction of longer PEG chains to enhance hydrophilicity, the dyes remained aggregated in PBS and BSA solutions causing fluorescence quenching, but more than 40% of the NIR‐II fluorescence could be retained in both MS and lipoprotein solutions. It should be noted that​ the fluorescence retention was below 0.1% for all dyes in PBS and for some in the BSA solution. These values are consequently shown as “0” in Figure 3b.

Subsequently, we investigated the targeting kinetics of different dyes with MS, HDL, and LDL at 37°C by measuring the time‐dependent changes in fluorescence intensity (Figure 3c–e). Obviously, as the length of the modified PEG chain increased, the time it took for the dye to reach equilibrium brightness in MS, HDL, and LDL was shorter, such as Flav12 and Flav45 reaching maximum brightness almost immediately after addition. To further quantify the binding process, we took advantage of the tendency of free dye to form large aggregates in aqueous solution. Mixtures of FlavAc (slow‐binding) or Flav45 (fast‐binding) with FBS were incubated at 37°C for varying durations and then subjected to high‐speed centrifugation (14,500 × g) to separate free dye. As shown in Figure S11a, the amount of precipitate visibly decreased over time for FlavAc, whereas no precipitate was observed for Flav45 at any time point. In both cases, the fluorescence intensity of the supernatant remained nearly unchanged before and after centrifugation (Figure S11b,e), indicating that the precipitated dyes contributed negligibly to the fluorescence signal. Based on this observation, we suggested that the precipitate represents free dye. The precipitates were redissolved in an equal volume of 2% (w/v) SDS solution, and the dissolved dye was quantified by absorption spectroscopy. Based on these measurements, the fraction of protein‐bound dye was calculated for each incubation time from standard curves of each dye in 2% SDS solution (Figure S11c,d,f,g). The results demonstrated that the binding ratio of FlavAc increased from 40% to 90% over time (from 5 min to 6 h), while Flav45 was almost fully bound within 5 min of mixing.

FlavAc with higher (50 µM) and lower (5 µM) concentrations was added to MS and lipoprotein solutions (Figure S12). When incubated at 37°C, the groups with lower concentrations of FlavAc rapidly reached the maximum fluorescence intensity, whereas those with higher concentrations required more time to achieve. But at elevated incubation temperatures, the maximum fluorescence intensity was attained within a shorter timeframe, independent of dye concentration. We further selected a representative subset of dyes to investigate the optimal loading concentration (Figure S13). The results showed that at physiological lipoprotein concentrations, the dye concentration required to achieve maximum fluorescence intensity in MS or lipoproteins was approximately 50 µM. As the dye concentration continued to increase, the overall fluorescence intensity decreased, which we speculated may be due to multiple dye molecules incorporating​​ into a single lipoprotein molecule, leading to intermolecular quenching.

### 2.5 Dispersion Behavior of Dyes and Specific Fluorescence Enhancement by Natural Lipoproteins

The evaluation of molecular dispersion behavior in PBS further elucidated the relationship between molecule structure and lipoprotein affinity. Initially, logP values of different dye structures were calculated using software to assess their hydrophilicity (Figure 3f). As designed, dyes with longer PEG chains exhibited higher hydrophilicity, with Flav45 showing greater hydrophilicity compared to the other dyes. Increased hydrophilicity enhanced the stability of molecular dispersion in aqueous solutions. We speculated that this hydrophilicity facilitated the proximity of the molecules to the hydrophilic side of the phospholipids on the surface of lipoproteins. The average hydrodynamic diameter of these dyes in PBS were further measured using DLS (Figure 3g and Figure S14). Dyes that exhibited faster binding kinetics with lipoproteins, such as Flav9, Flav12, and Flav45, formed particles with sizes smaller than 100 nm in PBS, and demonstrated greater stability in water, instead of tending to form larger aggregates and precipitate. By molecular dynamics simulation, we assessed the molecular aggregation states over time within a water box (Figure S15). After 20 ns, all the molecules exhibited aggregation in water. The mean squared displacement (MSD) analysis indicated that only Flav45 quickly reached equilibrium and maintained a stable presence (Figure 3h). Flav45 was recognized as the optimal molecule for rapid incorporation into lipoproteins.

To further validate the specificity of lipoprotein‐incorporation of the NIR‐II dye was only suitable for natural lipoproteins, we evaluated the dye binding ability of artificially synthesized lipoproteins, which simulate lipoproteins with different components, as well as co‐assembly strategy (Figure S16a). Compared to the natural lipoproteins in serum, these artificial approaches exhibited weaker fluorescence enhancement when incubated with dye (Figure S16b, c). Furthermore, these methods failed to generate nanoparticles with sizes comparable to those of natural lipoproteins (Figure S16d). This also demonstrated that our lipoprotein‐incorporation strategy based on in situ seeking of lipoproteins is simpler, cost‐effective, safer, and more controllable.

### 2.6 Biosafety of Lipoprotein‐Seeking Dyes and Photostability for Distinguishing Lipoprotein Types

We selected both lipoprotein slow‐binding and fast‐binding dyes (FlavAc and Flav45) to assess biocompatibility. Pathological analysis of the major organs from mice injected with Flav45 and FlavAc was performed, and H&E staining showed no obvious lesions compared to the control group (Figure S17a). Lower photostability is an inherent drawback of polymethine cyanine dyes. These molecules have been reported to be​ susceptible to photochemical reactions that generate reactive oxygen species (ROS) upon laser irradiation, which can break the polymethine chain and lead to potential biotoxicity [46, 48, 49]. We evaluated the reactive oxygen species (ROS) generation of Flav45 and FlavAc in DMSO under continuous irradiation using 1,3‐diphenylisobenzofuran (DPBF). The results indicated that the characteristic absorption peak at 410 nm completely disappeared within 20 min, confirming that our compounds also suffer from the same drawback of low photostability and ROS generation, which is consistent with the expected behavior of polymethine cyanine dyes (Figure S17b). However, the addition of FBS enhanced the photostability of both Flav45 and FlavAc (Figure S17c). The MTT assay was used to assess the impact of laser‐induced photobleaching on cytotoxicity (Figure S17d). Regardless of laser exposure, the groups supplemented with FBS exhibited lower cytotoxicity. Furthermore, Flav45 demonstrated increased cytotoxicity against 4T1 cells, which we speculated was due to the selective cytotoxic effects of the flavonoid structure on cancer cells. In summary, the interaction of the dyes with lipoproteins increased photostability, reducing the biological toxicity of the dyes.

We next investigated the photostability of Flav45 in various solutions (Figure S18a). Unexpectedly, Flav45 exhibited different half‐lives when mixed with HDL, LDL, and the equal volume mixture of HDL and LDL (Figure S18b). We further explored the brightness decay of Flav45 upon laser irradiation after mixing with different volumetric ratios of HDL and LDL (Figure S18c). As the proportion of HDL increased, the half‐life decreased from 90 to 30 min. Moreover, under laser irradiation at a fixed power density, the half‐life showed a linear relationship with the HDL ratio (Figure S18d). Next, we attempted to determine the linear response range of the dye to lipoprotein concentration (Figure S18e,f), where the lipoprotein cholesterol concentration detected by the assay kit served as a proxy for the actual lipoprotein concentration. The fluorescence intensity was found to be linearly correlated with lipoprotein content in the ranges of 0.005–0.012 mM (high‐density lipoprotein cholesterol, HDL‐C) and 0.01–0.03 mM (low‐density lipoprotein cholesterol, LDL‐C). Referencing the concentration of lipoproteins in serum, this suggests that Flav45 can respond to small sample amounts effectively through dilution. This encouraged us to validate this relationship using human serum samples (Figure S18g). Fresh human serum was divided into two portions: one portion was not diluted followed by Flav45 added, with fluorescence intensity recorded before and after a fixed duration of laser irradiation (FL1 and FL2). The other portion was diluted 150‐fold before the addition of Flav45, and its fluorescence intensity was also recorded (FL). The results indicated that the fluorescence decay rate showed a strong correlation with the proportion of HDL‐C in the total HDL‐C and LDL‐C as determined by the clinical assay (Figure S18h), and FL was associated with the total cholesterol (TC) content in serum (Figure S18i). Due to the complexity of blood lipid composition, considering only the contributions of HDL and LDL to the fluorescence intensity of Flav45 is insufficient to infer the proportion of various lipoproteins in serum. However, these findings suggest that Flav45 demonstrates a rapid and sensitive response to lipoprotein concentrations and holds potential for distinguishing lipoprotein types and concentrations based on concentration‐dependent responses and LDL‐selective photostability.

### 2.7 In Vivo Pharmacokinetics and High‐Contrast Angiography of Lipoprotein‐Seeking Dyes

Evaluating the metabolic clearance and imaging capability of imaging agents in vivo is essential (Figure 3i). NIR‐II imaging of mice after intravenous injection of the dyes revealed that the fluorescence signal was primarily distributed in the liver, and it gradually cleared until undetectable seven days post‐injection (Figure 3j; Figure S19). At an equivalent injection dose, dyes with stronger lipoprotein‐binding affinity (e.g., Flav9 and Flav45) exhibited more intense hepatic fluorescence that appeared more rapidly, while the control group (Flav) showed nearly undetectable fluorescence. Subsequently, mice were euthanized at 2 and 14 days post‐injection, and major organs were harvested to evaluate the clearance rate of the dyes. The results showed that the fluorescence intensity in the liver decreased significantly by day 14 compared to day 2, indicating that the dye was almost completely cleared (Figure S20). To further evaluate the blood clearance kinetics of the dyes, we collected blood samples at various time points after intravenous administration and quantified the fluorescence intensity. The results showed that dyes with rapid lipoprotein‐binding kinetics exhibited faster blood signal activation and clearance (Figure 3k). Finally, we assessed the vascular imaging capability of these lipoprotein‐seeking dyes via hindlimb vessel imaging in mice (Figure S21). Dyes with rapid lipoprotein binding rate, such as Flav9 and Flav45, exhibited bright NIR‐II vascular imaging. In contrast, dyes with slower lipoprotein binding rate, such as Flav7 and FlavAc, were able to gradually illuminate the vessels after injection but the fluorescence intensity was comparatively weaker. Notably, we selected images captured at the optimal imaging time for each dye and calculated the fluorescence intensity ratio between limb vasculature and skin. Nearly all dyes enabled high‐contrast vascular imaging due to their lipoprotein‐incorporation properties (Figure S22).

### 2.8 Lipoprotein‐Seeking Dye for NIR‐II Real‐Time Accurate Visualization of Early Fatty Liver Disease

**FIGURE 4:**
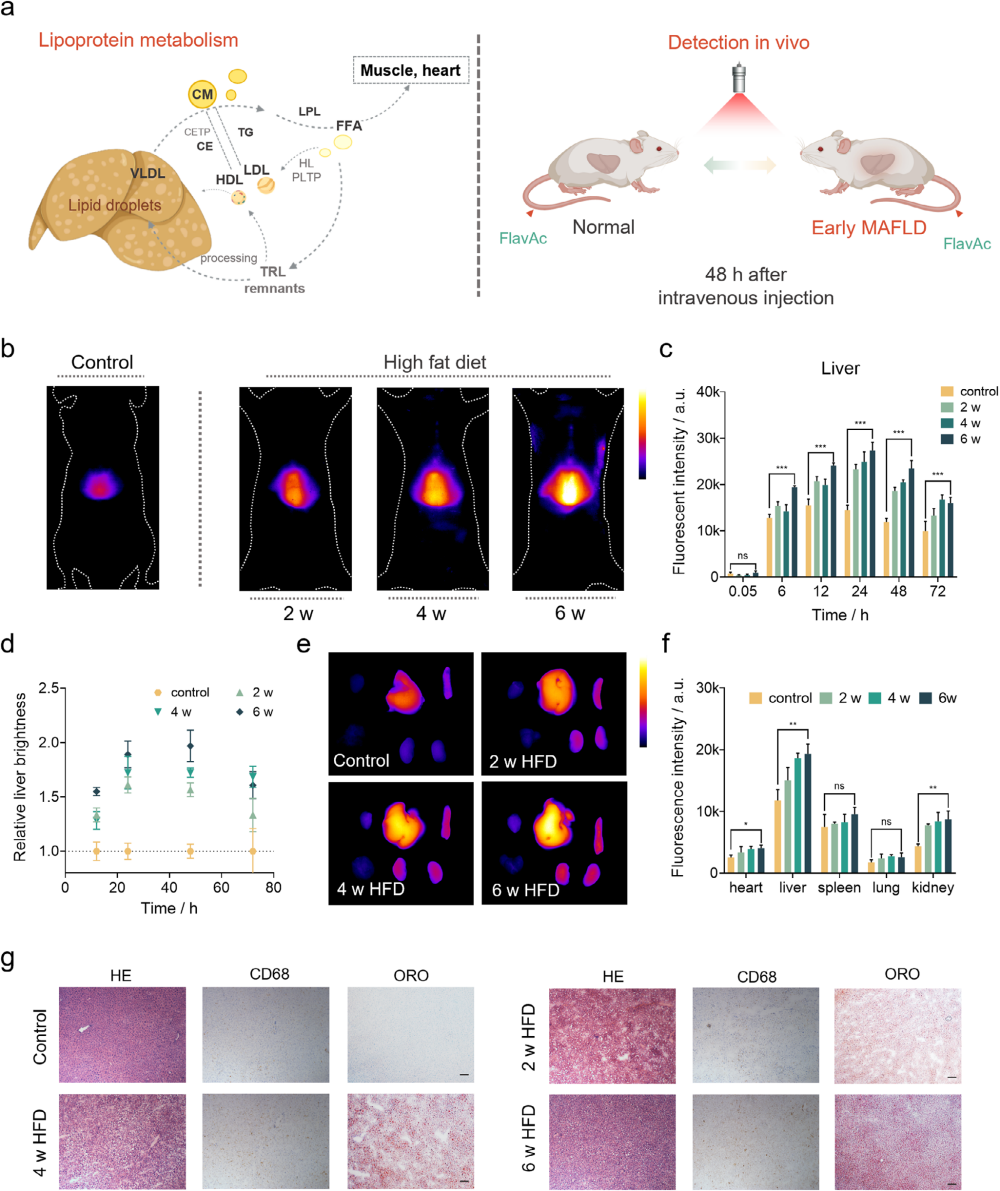
Lipoprotein‐seeking dyes for early fatty liver monitoring. (a) The diagram of metabolic‐associated fatty liver disease and the detection of dyslipidemia and fatty liver. VLDL: very low‐density lipoproteins; CM: chylomicrons; CETP: cholesterol ester transfer protein; CE: cholesterol ester; TG: triglyceride; LPL: lipoprotein lipase; FFA: free fatty acids; PLTP: phospholipid transfer protein; HL: hepatic lipase; TRL: triglyceride‐rich lipoproteins. Created in BioRender. (b) NIR‐II imaging of living mice in the control group and the HFD group after tail vein injection of FlavAc. Exposure time: 10 ms. (c) NIR‐II fluorescence intensity statistics of mice liver 48 h after injection (n = 3). (d) Relative liver brightness after injection in mice under high‐fat diet (n = 3). The signal in high‐fat diet groups is shown relative to the control group (set as 1.0). (e) NIR‐II imaging of main organ ex vivo after injecting FlavAc for 48 h. Exposure time: 5 ms. (f) NIR‐II Fluorescence intensity statistics of major organ ex vivo after injecting dyes for 48 h (n = 3). (g) Hematoxylin and eosin (H&E), CD68, and Oil Red O (ORO) staining of liver sections with different degrees of fat accumulation. Injection dose: 0.6 µmol kg^−1^; over 1100 nm; power density: 65 mW cm^−2^. Significance was defined as *p < 0.05, **p < 0.01, ***p < 0.001, ****p < 0.0001.

The liver is a critical organ in lipoprotein metabolism, and systemic lipid metabolism disorders, including elevated blood lipids and hepatic fat accumulation, are key markers of fatty liver disease progression [2, 50, 51]. Lipoproteins serve as carriers for lipid exchange between the liver, blood, and peripheral organs [52]. We aimed to utilize this lipoprotein‐seeking probe as a tool for non‐invasive monitoring of fatty liver disease progression (Figure 4a). To evaluate the monitoring capability of the lipoprotein‐seeking dye for early non‐alcoholic fatty liver disease, we observed the changes in hepatic NIR‐II signals in C57BL/6J mice after intravenous injection of FlavAc (2 mg kg^−1^). These mice were fed a high‐fat diet for durations of two, four, and six weeks and the mice exhibited a stable increase in body weight (Figure S23). Meanwhile, the control group mice were maintained on a normal diet. The NIR‐II brightness of liver in the HFD group significantly increased compared to the control group mice 48 h after injection (Figure 4b,c; Figure S24). The liver signal ratios between model mice and normal mice were quantified at key time points (Figure 4d). The results showed that, the distinction was observed as early as two weeks into the high‐fat feeding regimen and intensified as the disease progressed. Furthermore, ex vivo fluorescence signals were obtained from major organs of mice 48 h post‐injection, and the fluorescence signals were quantified (Figure 4e,f). All organs in the HFD group exhibited increased fluorescence signals, with significant accumulation observed in the liver and kidneys, which are the main organs affected by lipid metabolism abnormalities. Hematoxylin and eosin (H&E) and Oil Red O (ORO) staining of liver tissue showed minimal lipid accumulation beginning at 2 weeks of HFD (Figure 4g). By 6 weeks of high‐fat feeding, small lipid droplets were present in the majority of liver cells. CD68 immunohistochemical staining indicated that inflammatory changes in the liver of mice increased with the duration of the high‐fat diet. Further quantification of ORO staining revealed a significant increase in hepatic fat content corresponding to the length of high‐fat feeding (Figure S25). However, no larger lipid vacuoles were observed in the sections, suggesting that the lipoprotein‐seeking probe is expected to serve as an ultra‐early warning tool for lipid metabolism disorders, such as fatty liver disease, occurring in the absence of inflammation.

### 2.9 Lipoprotein‐Seeking Dyes for NIR‐II Long‐Term Targeted Imaging of Atherosclerotic Plaques

**FIGURE 5:**
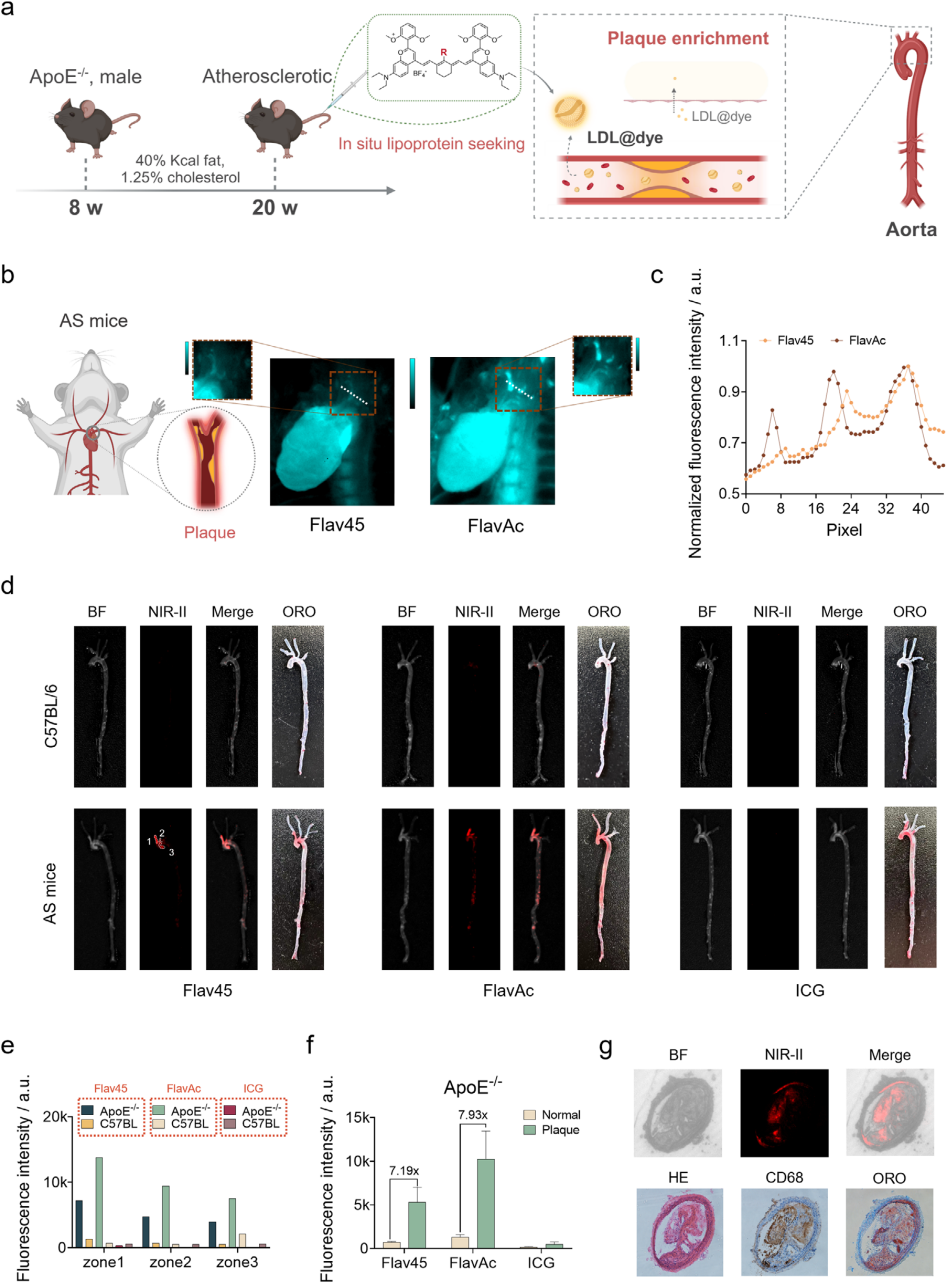
NIR‐II imaging of atherosclerotic plaques. (a) The diagram of accumulation of lipoprotein‐seeking dyes in aortic plaques. LDL is proposed as the main carrier mediating dye accumulation in plaques. Created in BioRender. (b) In situ NIR‐II imaging of an intact aorta isolated from an AS mouse. AS: atherosclerosis. (c) NIR‐II fluorescent cross‐sectional intensity profile of whole‐body vessels from b. (d) Ex vivo aortas imaging from C57BL/6J (control) and ApoE^−/−^ mice after 12 weeks of high‐fat diet feeding and intravenous injection of FlavAc, Flav45, and ICG for 24 h. Injection dose: 600 µM, 200 µL. (e) NIR‐II fluorescence intensity of different plaque zone from d. (f) NIR‐II fluorescence intensity of plaque and normal vascular tissue in ApoE^−/−^ mice from d (n = 3). (g) Comparison of NIR‐II imaging, H&E staining, ORO staining, and CD86 staining for arterial plaque from arterial plaque.

Atherosclerosis is a leading cause of cardiovascular disease‐related mortality worldwide [53]. Early detection and intervention are essential for reducing these mortality rates [54]. LDL naturally accumulates in atherosclerotic plaques, where it undergoes oxidation and contributes to disease progression [55]. This property positions LDL as a potential natural carrier for delivering contrast agents and therapeutic drugs. However, there are few dyes that can specifically incorporate into lipoproteins in vivo. To address these challenges, the use of lipoprotein‐seeking dyes for in situ incorporation into lipoproteins presents a safer and more precise approach. These dyes can accumulate in plaques by targeting endogenous lipoproteins, thus facilitating the monitoring of atherosclerosis progression (Figure 5a). After 12 weeks on a high‐fat diet for ApoE^−/−^ mice (Figure 5b), tail vein injections of FlavAc or Flav45 resulted in obvious NIR‐II fluorescence accumulation in the aortic arch 24 h post‐injection, which is one of the primary sites of plaque formation in the atherosclerotic mouse model. Statistical analysis revealed that the plaque region exhibited a distinguishable signal‐to‐noise ratio (1.3–1.5) compared to the normal region (Figure 5c).

To further confirm the accuracy of targeted labeling of plaques, we conducted ex vivo NIR‐II imaging of the aortic vasculature in the model mice while employing ORO staining for plaque identification, comparing the results with those from C57BL/6J mice that do not exhibit a risk of atherosclerosis (Figure 5d). The results revealed a strong correlation between the NIR‐II fluorescence regions and both visually identifiable plaques and ORO‐stained areas. This suggested that the lipoprotein‐seeking dye effectively targeted atherosclerotic plaques, enabling high‐contrast in situ imaging of these lesions. In contrast, very weak fluorescence was detected in the entire aorta of the C57BL/6J mice. ICG, a widely used NIR fluorescent dye for clinical near‐infrared imaging, has been reported to bind to various plasma proteins. After incubation of ICG with rat, rabbit, and pig serum, agarose gel electrophoresis showed that the fluorescent signal was concentrated at the albumin and lipoprotein bands (Figure S26). Although ICG was able to bind to lipoproteins, its non‐specific binding and rapid clearance from the bloodstream made it difficult for targeting imaging of plaques. NIR‐II fluorescence intensity was quantified in three characteristic regions of plaque formation (Figure 5e). In the similar regions, fluorescence enhancement was observed in the model group following the injection of Flav45 and FlavAc, while ICG exhibited low fluorescence signals. The fluorescence intensity of the plaque area was more than 7 times that of the normal area in aorta of AS mice injected with lipoprotein‐seeking dye (Figure 5f). Surprisingly, these dyes provided long‐term targeted labeling of plaques. They were still able to precisely localize plaque positions 10 d post‐administration while maintaining severalfold brightness compared to normal tissue (Figure S27). Furthermore, to investigate the details of fluorescence distribution, we collected the aorta from atherosclerotic mice and normal C57BL/6J mice 24 h after the injection of FlavAc, subsequently preparing frozen sections for microscopy imaging. Notably, the specific distribution of NIR‐II fluorescence signals was observed within the plaques (Figure 5g; Figure S28). H&E staining, along with CD68 and ORO staining, revealed a distribution of atherosclerotic lesions that corresponded with the NIR‐II fluorescence signals. Regrettably, the strong NIR‐II signals were concentrated on the side of the plaque in contact with blood and gradually decreased with increasing plaque depth. This is speculated to be due to the lipoproteins carrying the dye being unable to transport and exchange into the plaque interior within a short time. But it is advantageous to rapidly label plaques by lipoprotein‐seeking dyes for the detection of early minor lesions.

## 3 Conclusion

In this study, we developed a class of NIR‐II dyes that specifically incorporate into lipoproteins in situ, providing a novel endogenous biomolecular platform to enhance the optical performance and targeted delivery of dyes in vivo. Flav modified with hydrophilic groups was validated through screening common molecular structures as the optimal choice for specific lipoprotein incorporation. The lipoprotein‐seeking properties of the dye were determined by the design of an appropriate molecular scaffold and the modification of hydrophilic groups. We also validated that modifying the length of mPEG to introduce varying hydrophilicity can influence the binding kinetics of the dye with lipoproteins. We proposed that increased hydrophilicity enhances the dispersion of dye molecules in aqueous solutions and reduces dye self‐aggregation; the introduction of hydrophilic groups may potentially facilitate interactions between the dye molecules and the hydrophilic phospholipids on the surface of lipoproteins in aqueous environments. Furthermore, we conducted a series of in vitro experiments that demonstrated the effectiveness of this lipoprotein‐seeking strategy in improving the brightness, photostability, and biosafety of dyes. Dyes encapsulated by different types of lipoproteins exhibit varying photostability, making them a potential method for screening and quantifying the subtypes of intact lipoprotein content. Utilizing​ its superior bioimaging resolution and lipoprotein‐specific labeling properties, this platform not only sensitively reflects the accumulation of liver fat in early‐stage fatty liver disease but also achieves longitudinal visualization of atherosclerotic plaques.

Overall, triggering selective dye‐lipoprotein interactions within the blood circulation system represents a more readily applicable approach that utilizes natural lipoprotein transport mechanisms, effectively enhancing targeted imaging of lipoprotein‐related conditions. The potential to visualize and monitor lipoprotein distribution and accumulation in vivo promises to enhance our understanding of lipid metabolism and its associated disorders, contributing to the improvement of cardiovascular healthcare.

## 4 Experimental Section

### 4.1 Materials

Bovine serum albumin (BSA, ≥ 98%, V90093), IR‐6B3 (IR‐780), IR‐6N4S (IR‐820), and L‐α‐Phosphatidylcholine were purchased from Sigma‐Aldrich. Cholesterol and cholesteryl ester were purchased from Macklin. Acetylcysteine, isobutylmercaptan, 1‐methyl‐2‐pyrrolidinone, 3‐mercaptopropionic acid, 2‐(7‐Azabenzotriazol‐1‐yl)‐N,N,N',N'‐tetramethyluronium hexafluorophosphate (DIPEA), Pd_2_(dba)_3_, XPhos, CH_3_MgBr, Cs_2_CO_3_, methoxypolyethylene glycol amine (M_w_ = 550, 2000), and anhydrous dimethyl sulfoxide (DMSO, ≥ 99%) were purchased from Energy Chemical. c(RGDfC) was purchased from Ruixibio. Fetal bovine serum (FBS), human high‐density lipoprotein (HDL) and low‐density lipoprotein (LDL) were purchased from Yeasen Biotechnology (Shanghai) Co., Ltd. Human IgG (SP001) was purchased from Beijing Solarbio Science & Technology Co., Ltd. Rat serum (RS) was purchased from Shanghai Yuanye Bio‐Technology Co., Ltd. Pig serum was purchased from Gibco.

### 4.2 Instrument Characterization

The UV‐absorption spectra of different probes were measured using a LAMBDA 1050+ spectrophotometer and Thermo Fisher Evolution 201 spectrophotometer. The NIR‐II fluorescence spectra of different probes were measured by Edinburgh instrument FLS920 fluorescence spectrophotometer. Sodium dodecyl sulfate‐polyacrylamide gel electrophoresis (SDS‐PAGE) was performed on different probes using an American BIO‐RAD electrophoresis system. The particle sizes of different compounds were analyzed using Malvern Zetasizer Nano ZS size analyzer. Transmission electron microscope (TEM) images were acquired through HT7800 of HITACHI Ltd. The ^1^H‐NMR and ^13^C‐NMR spectra of all dyes were obtained on Bruker AVANCE III 400 MHz NMR spectrometers. Matrix‐Assisted Laser Desorption/ Ionization Time of Flight Mass Spectrometry (MALDI‐TOF‐MS) was performed on a Bruker Microflex TOF using *trans*‐2‐[3‐(4‐tert‐butylphenyl)‐2‐methyl‐2‐propenylidene] malononitrile (DCTB) as a matrix. High‐resolution mass spectrum (HRMS) was operated under the specific conditions (ESI^+^ spray voltage, 4.5 kV, or ESI^−^ spray voltage, −3.5 kV; nebulizer gas, 1.5 L min^−1^; drying gas, 100 kPa; heat block temperature, 200°C; CDL temperature, 200°C; IT Area Vacuum, 1.0 × 10^−2^ Pa; TOF Area Vacuum, 5 × 10^−4^ Pa). The ion accumulation time was set to 10 ms, and the detector voltage was fixed at 1.6 kV. Chromatography of all dyes were obtained on Agilent 1260 Infinity.

### 4.3 Synthesis Procedure of NIR‐II Dyes

The polymethine linker and heterocycles were conjugated through Knoevenagel reaction. The thiol‐containing substituent was introduced to the chloro‐cyclohexene through nucleophilic substitution reaction under basic catalysis. The detailed synthesis protocols for each NIR‐II dye were documented in the supplementary information.

### 4.4 Chromatographic Separation Conditions

Separation of IR‐6B3Ac, IR‐6B6CAc, IR‐6N3Ac, IR‐6N4SAc, IR‐6B9Ac, FlavAc, FlavBu, FlavRGD, and PhFlavAc were carried out on a YMC‐Pack ODS‐A (250 mm × 4.6 mm; particle size 5 µm) obtained from YMC Co., Ltd. Separation of IR‐6B12Ac, Flav2, Flav3, Flav7, Flav9, Flav12, and Flav45 were carried out on a InertSustainSwift C18 (250 mm × 4.6 mm; particle size 5 µm) obtained from GL Sciences (Shanghai) Ltd.

Separation conditions of IR‐6B3Ac, IR‐6B6CAc, IR‐6N3Ac, and IR‐6N4SAc: The mobile phase consisted of (A) H_2_O with 0.1% TFA and (B) acetonitrile with 0.1% TFA, delivered at a flow rate of 1 mL min^−1^ under the following gradient program: initial composition of 15% B was held for 3 min, ramped to 90% B over 7 min (time: 10 min), maintained for 9 min, and finally returned to 15% B for a 1 min column re‐equilibration.

Separation conditions of IR‐6B9Ac: The mobile phase consisted of (A) H_2_O with 0.1% TFA and (B) acetonitrile with 0.1% TFA, delivered at a flow rate of 1 mL min^−1^ under the following gradient program: initial composition of 15% B was held for 3 min, ramped to 90% B over 7 min (time: 10 min), maintained for 20 min, and finally returned to 15% B for a 1 min column re‐equilibration.

Separation conditions of IR‐6B12Ac: The mobile phase consisted of (A) H_2_O with 0.1% TFA and (B) methanol with 0.1% TFA, delivered at a flow rate of 1 mL min^−1^ under the following gradient program: initial composition of 80% B was held for 6 min, ramped to 100% B over 1 min (time: 7 min), maintained for 20 min, and finally returned to 80% B for a 1 min column re‐equilibration.

Separation conditions of FlavAc, PhFlavAc, and FlavRGD: The mobile phase consisted of (A) H_2_O with 0.1% TFA and (B) acetonitrile with 0.1% TFA, delivered at a flow rate of 1 mL min^−1^ under the following gradient program: initial composition of 15% B was held for 3 min, ramped to 90% B over 7 min (time: 10 min), maintained for 5 min, then ramped to 100% B over 10 min (time: 25 min), and finally returned to 15% B for a 1 min column re‐equilibration.

Separation conditions of FlavBu: The mobile phase consisted of (A) H_2_O with 0.1% TFA and (B) acetonitrile with 0.1% TFA, delivered at a flow rate of 1 mL min^−1^ under the following gradient program: initial composition of 15% B was held for 3 min, ramped to 90% B over 7 min (time: 10 min), then ramped to 100% B over 5 min (time: 15 min), maintained for 15 min, and finally returned to 15% B for a 1 min column re‐equilibration.

Separation conditions of Flav2: The mobile phase consisted of (A) a 1:1 (v/v) mixture of water and methanol and (B) methanol, delivered at a flow rate of 1 mL min^−1^ under the following gradient program: initial composition of 60% B was held for 6 min, ramped to 100% B over 1 min (time: 7 min), maintained for 23 min, and finally returned to 60% B for a 1 min column re‐equilibration.

Separation conditions of Flav3, Flav7, Flav9, Flav12, and Flav45: The mobile phase consisted of (A) a 1:1 (v/v) mixture of water and methanol and (B) methanol, delivered at a flow rate of 1 mL min^−1^ under the following gradient program: initial composition of 80% B was held for 6 min, ramped to 100% B over 1 min (time: 7 min), maintained for 23 min, and finally returned to 80% B for a 1 min column re‐equilibration.

### 4.5 Animal Models

Balb/c (female, 6–8 weeks old) and C57BL/6J mice (female and male, 6–8 weeks old) were purchased from Jilin Qianhe Biotechnology Co., Ltd. ApoE^−/−^ mice (male, 8 weeks old) were purchased from and Beijing Vital River Laboratory Animal Technology Co., Ltd. All animals were housed under controlled conditions (20–22°C, 35%–45% humidity, 12 h light/dark cycle) with free access to food and water. Only mice meeting the health criteria (body weight 18–20 g with normal activity levels) were included in the study.

Specific experimental groupings were as follows: In the metabolism study, Balb/c mice were randomly divided into 3 groups (n = 7 per group) for long‐term distribution observation following injection of one of seven dyes. For blood clearance and hindlimb vascular imaging, C57BL/6 mice (female) were randomly allocated into 6 groups (n = 6 per group); three groups injected one of six different dyes for serial blood collection, and the remaining three groups were used for hindlimb vascular imaging. In the fatty liver model, C57BL/6 mice (male) were randomly assigned to 4 groups (n = 6 per group) and fed either a high‐fat diet (XTHF60, 60% calories from fat, from Xietong Pharmaceutical Bio‐engineering Co., Ltd.) for 2, 4, or 6 weeks, or a standard chow diet as control. For atherosclerosis imaging, ApoE^−/−^ mice fed a high‐fat diet for 12 weeks were randomly divided into 3 groups (n = 3 per group) for injection of three dyes. Prior to NIR‐II imaging, mice were anesthetized using isoflurane and depilated with hair removal cream over the entire body except the head. During imaging, anesthesia was maintained, and the animals were allowed to recover freely afterward. At the designated experimental endpoints or specific time points, euthanasia was performed via CO_2_ inhalation. Death was confirmed before tissue samples were collected.

### 4.6 NIR‐I/II Imaging

NIR‐II imaging set‐up was home‐built and consisted of a camera (Princeton Instruments, NIRvana‐640; Raptor), laser, and off‐the‐shelf optics (Thorlabs, Edmund optics, etc.). The excitation light was generated by 808 or 980 nm fiber‐coupled diode laser with adjustable power density. Different long‐pass (LP) filters were combined to capture different waveband images in the NIR‐II window. All mice were shaved and depilated by depilatory cream before imaging and anesthetized with isoflurane for the procedure. All fluorescence images were processed and analyzed by Image J software.

### 4.7 Lipoprotein Electrophoresis on Agarose Gel

1% agarose gel electrophoresis separates plasma lipoproteins (VLDL, LDL, and HDL) according to their electrostatic charges. 1 µL Sudan black dye solution was added to 10 µL lipoprotein or serum and incubated at 37°C in a dark place for 30 min. Then the system was centrifuged for 10 min (3000 rpm) to remove the insoluble solid dye. 10 µL of each experimental condition were loaded onto the agarose gel. Following electrophoresis at 100 V for 40 min, the gel was imaged for NIR‐II fluorescence and by bright‐field microscopy. Superimposing the fluorescence and the black/white images allowed the determination of possible dyes‐lipoprotein interactions.

### 4.8 Microscale Thermophoresis (MST)

To determine the solution equilibrium binding constant between the analyte and the protein, we employed Microscale Thermophoresis (MST). The analyte was used as the ligand, and the protein served as the target. The protein was labeled using the RED‐NHS protein labeling kit. A 16‐point, two‐fold serial dilution of the analyte was prepared in the reaction buffer (50 mM HEPES [pH 7.4] containing 0.05% Tween 20) and mixed with an equal volume of the labeled protein. The mixture was incubated at room temperature for 20 min. Subsequently, the samples were loaded into capillaries, and the thermophoretic signals were measured using a Monolith X instrument (NanoTemper) following the manufacturer's standard protocol. The dissociation constant (Kd) was determined using the NanoTemper analysis software.

### 4.9 Measurement of the Dye Binding Ratio in Serum

The dye (10 µM) was thoroughly mixed with FBS (500 µL) and incubated at 37°C for 5 min, 1, 2, 4, and 6 h, respectively. After incubation, the mixture was centrifuged at 14,500 × g and 5°C for 20 min to separate the free dye. The supernatant was carefully removed, and the pellet was resolved in 400 µL 2% (w/v) SDS solution. The absorption spectrum of the resulting solution was acquired using a UV–Vis spectrophotometer. The concentration of the free dye in the original system was calculated based on a standard curve of the dye prepared in 2% SDS solution. The fraction of binding dye was then calculated as follows:

(1) $$Binding\;ratio\;\left(\%\right)=\frac{C_{total}-C_{free\;dye}}{C_{total}}\;\times 100\%$$

### 4.10 MD Simulation

All MD simulations were performed with the LAMMPS [56]. Ovito software was applied for trajectory visualization and analysis. The system consisted of solid and ions solvent. The GAFF force field was used for Flav2, Flav3, Flav7, Flav9, Flav12, Flav45, FlavAc, and FlavRGD. The water molecules were described by the TIP3P [Comparison of simple potential functions for simulating liquid water]. Hydrogen atomic positions were kept rigid with the SHAKE and RATTLE algorithms [57]. The non‐bonded van der Waals interactions were molded using the 12–6 Lennard‐Jones potential, while the electrostatic interactions, such as long‐range Coulomb interactions, were addressed by the particle‐particle‐particle mesh technique [58]. Nonbonded interactions were calculated with a 12 Å atom‐based cutoff, correcting for the long‐range electrostatics.

The nine systems are solution models, where nine simulation boxes all have a size of 10.0 nm × 10.0 nm × 10.0 nm. The nine systems each contained 6 polymer molecules and 30000 water molecules. In the production run, a time step of 1 fs was used, and the data were collected every 1 ps. The system was minimized (atomic positions and cell sizes), keeping the box length isotropic. For each system, two independent trajectories of 20 ns were generated. All production runs were under in microcanonical ensemble at 298 K and 1 bar pressure for 10 ps to equilibration. Then a microcanonical ensemble at 298 K was performed to obtain the parametric of the system for 20 ns. Periodic boundary conditions were applied in all directions.

### 4.11 ROS Detection

DPBF solution (0.1 mM, dissolved in DMSO) was prepared in the dark and Flav45 or FlavAc solution in DMSO (0.1 mM) was prepared. Then the mixture was irradiated continuously under NIR laser (65 mW cm^−2^) until the characteristic absorption peak almost disappear. The generation of ROS was demonstrated by the characteristic absorption decrease of the DPBF at 410 nm by a UV–vis absorption spectrum [59].

### 4.12 Cell Culture and Cell Viability

Mouse breast cancer cell line (4T1) was purchased from the Shanghai Enzyme Research Biotechnology Co., LTD (Shanghai, China). Mouse fibroblasts cell line (L929) was kindly provided by the Joint Laboratory of Opto‐Functional Theranostics in Medicine and Chemistry, First Hospital of Jilin University. 4T1 cell line and L929 cell line were cultured in DMEM (containing 80 U mL^−1^ penicillin and 0.08 mg mL^−1^ streptomycin) supplemented with 10% (v/v) FBS in a relatively humidified atmosphere of 5% CO_2_ at 37°C. The phototoxicity of Flav45 and Flav45@FBS in two cell lines (L929 and 4T1) was evaluated using the MTT assay. L929 cells and 4T1 cells were seeded in 96 well plates at a cell density of 1×10^4^ cells/well and both were incubated until 80% confluent. Subsequently, the mixed solution Flav45 and DMEM medium at the concentrations of 0, 1, 10, 20, 50, and 100 µM were added to the wells and incubated for 12 h. One group was placed under a 980 nm laser for 30 min before incubation (power density: 65 mW cm^−2^). The cells without treatment were used as controls. MTT (200 µL, 0.5 mg mL^−1^, DMSO) was added to each well and incubated for 4 h. Finally, the absorbance at 570 nm was measured by a microplate reader (Bio‐Tek, Synergy LX, USA). The cytotoxicity was evaluated based on the absorption values.

### 4.13 Human Serum Sample

This study analyzed a total of 20 human serum samples from individuals of Asian descent (13 females and 7 males). The participants were categorized into four age groups: 0–19 years (n = 3), 20–39 years (n = 5), 40–59 years (n = 2), and 60–80 years (n = 10).

### 4.14 Statistical Analyses

Data points were collected and compiled in Microsoft Excel, and statistical analyses were performed using GraphPad Prism and Origin Pro software. Statistical significance was determined by a student's t‐test. In all cases, significance was defined as *p < 0.05, **p < 0.01, ***p < 0.001, ****p < 0.0001. For the continuous variables, the data were presented as mean ± standard deviation.

## Ethics Statement

All animal experiments were conducted according to the protocols approved by the Animal Ethical Committee of The First Hospital of Jilin University (20210642). The protocol for using human blood was approved by the ethics committee of the First Hospital of Jilin University (22K006‐002).

## Conflicts of Interest

The authors declare no conflict of interest

## Supporting information




**Supporting File**: advs74028‐sup‐0001‐SuppMat.docx.

## Data Availability

The data that support the findings of this study are available from the corresponding author upon reasonable request.
