# Supplementary material for: In Situ Lipoprotein‐seeking Dye for in Vivo Real‐Time Imaging of Lipid Dysregulation Diseases
Source: Adv Sci (Weinh). 2026 Jan 27;13(19):e14290. doi: 10.1002/advs.202514290 (PMC13045373; doi:10.1002/advs.202514290)
Supplement: Supplementary file 1 — Supporting File: advs74028‐sup‐0001‐SuppMat.docx. [file ADVS-13-e14290-s001.docx]

Supporting Information

**In Situ Lipoprotein-seeking Dye for in Vivo Real-Time Imaging of Lipid Dysregulation Diseases**

*Yijing Du, Zetao Dang, Baofeng Xu, Jiajun Xu, Dan Wang, Yuewei Zhang, Shoujun Zhu^*^***General Procedure for Synthesis of NIR-II Dyes**

**1. Synthesis of DyeAc and DyeBu**


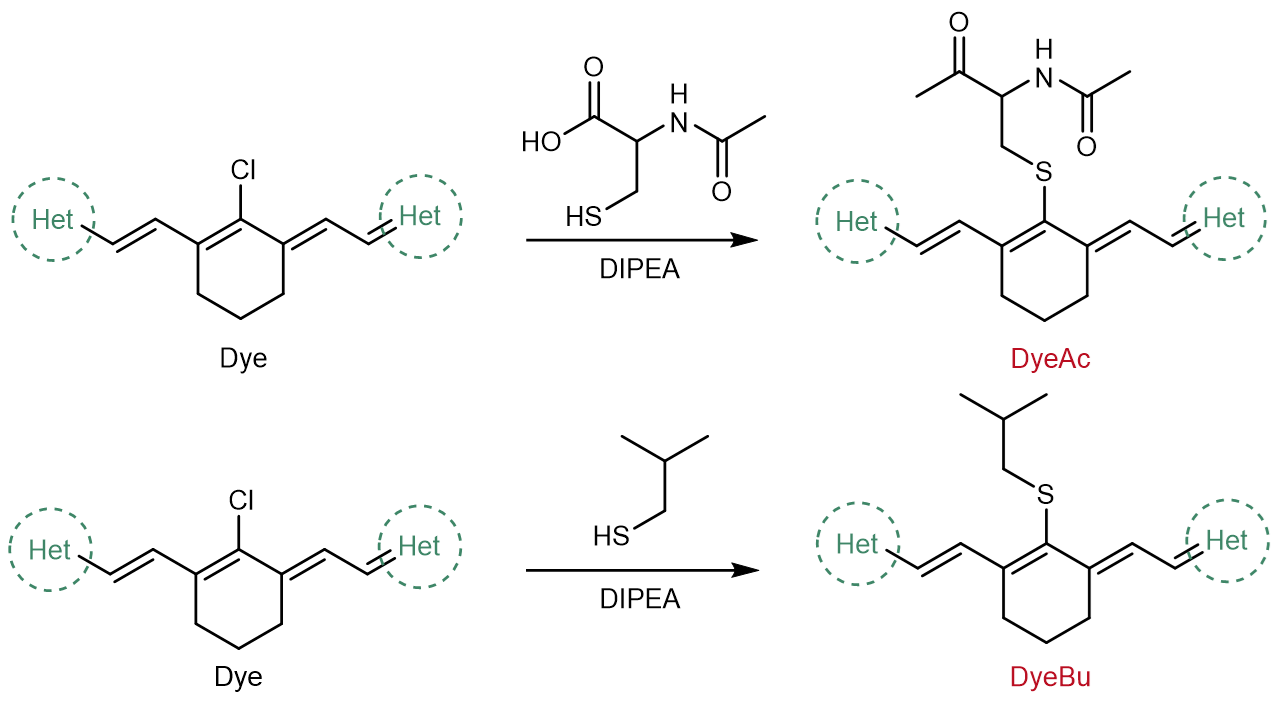


**Scheme S1.** General synthetic route of DyeAc and DyeBu.

**Synthesis of IR-6B3Ac, IR-6B6CAc, IR-6N3Ac, IR-6N4SAc, IR-6B9Ac, and IR-6B12Ac.** Dye (0.013 mmol) and acetylcysteine (0.03 mmol) were dissolved in 1 mL dimethyl sulfoxide (DMSO). Then added 5 μL N, N-Diisopropylethylamine (DIPEA) and stirred at room temperature for 1 h. The crude product was purified by column chromatography on silica gel using DCM/MeOH. IR-6N3, IR-6B9, and IR-6B12 were easily synthesized according to the previous report.^1^

^1^H NMR of IR-6B3Ac (400 MHz, CDCl_3_) δ 8.88 (d, *J* = 14.0 Hz, 2H), 7.42 (d, *J* = 7.4 Hz, 2H), 7.38 (t, *J* = 7.6 Hz, 2H), 7.23 (t, *J* = 7.4 Hz, 2H), 7.07 (d, *J* = 7.9 Hz, 2H), 6.05 (d, *J* = 14.0 Hz, 2H), 4.45 (s, 1H), 4.01 (t, *J* = 7.6 Hz, 4H), 3.64 (s, 2H), 2.59 (s, 4H), 1.96 (s, 2H), 1.92 (dd, *J* = 15.5, 8.3 Hz, 4H), 1.80 (s, 12H), 1.33 (s, 3H), 1.09 (t, *J* = 7.4 Hz, 6H), 0.91 (t, *J* = 6.7 Hz, 4H). LC-HRMS (ESI-TOF): calcd. for C_41_H_52_N_3_O_3_S^+^ ([M]^+^) 666.3724; found 666.3733.

^1^H NMR of IR-6B6CAc (400 MHz, DMSO-*d_6_*) δ 8.62 (d, *J* = 14.0 Hz, 2H), 7.59 (d, *J* = 7.3 Hz, 2H), 7.39 (d, *J* = 3.1 Hz, 4H), 7.30 – 7.17 (m, 2H), 6.24 (d, *J* = 14.1 Hz, 2H), 4.19 (d, *J* = 6.3 Hz, 4H), 4.00 (d, *J* = 6.3 Hz, 1H), 3.13 (dd, *J* = 12.9, 6.6 Hz, 2H), 2.58 (s, 4H), 2.19 (t, *J* = 7.2 Hz, 4H), 1.78 (s, 2H), 1.75 (d, *J* = 7.3 Hz, 3H), 1.67 (s, 12H), 1.55 (q, *J* = 7.4 Hz, 4H), 1.36 (q, *J* = 7.4 Hz, 4H). LC-HRMS (ESI-TOF): calcd. for C_47_H_60_N_3_O_7_S^+^ ([M]^+^) 810.4146; found 810.4135.

^1^H NMR of IR-6N3Ac (400 MHz, MeOD) δ 8.94 (d, *J* = 14.0 Hz, 2H), 8.29 (d, *J* = 8.6 Hz, 2H), 8.00 (t, *J* = 9.0 Hz, 4H), 7.76 – 7.56 (m, 4H), 7.48 (t, *J* = 7.5 Hz, 2H), 6.31 (d, *J* = 14.3 Hz, 2H), 4.50 – 4.45 (m, 1H), 4.45 – 4.39 (m, 2H), 4.25 (t, *J* = 7.3 Hz, 4H), 2.92 (t, *J* = 6.1 Hz, 3H), 2.70 (d, *J* = 6.6 Hz, 4H), 2.07 (d, *J* = 1.8 Hz, 12H), 1.98 (s, 4H), 1.95 – 1.92 (s, 2H), 1.10 (t, *J* = 7.4 Hz, 6H). LC-HRMS (ESI-TOF): calcd. for C_49_H_56_N_3_O_3_S^+^ ([M]^+^)766.4037; found 766.3951.

^1^H NMR of IR-6N4SAc (400 MHz, MeOD) δ 8.99 (d, *J* = 14.2 Hz, 2H), 8.34 (d, *J* = 9.0 Hz, 2H), 8.03 (t, *J* = 9.0 Hz, 4H), 7.75 – 7.62 (m, 4H), 7.51 (t, *J* = 7.4 Hz, 2H), 6.37 (d, *J* = 14.4 Hz, 2H), 4.39 – 4.32 (m, 4H), 2.99 – 2.91 (m, 4H), 2.76 (d, *J* = 6.3 Hz, 4H), 2.12 (s, 12H), 2.02 (s, 4H), 1.63 (s, 1H), 1.41 (d, *J* = 3.5 Hz, 2H), 1.32 (s, 4H), 1.02 – 0.85 (m, 3H). LC-HRMS (ESI-TOF): calcd. For C_51_H_60_N_3_O_9_S_3_^+^ ([M+2H]^+^) 954.3486; found 954.3421.

^1^H NMR of IR-6B9Ac (400 MHz, CDCl_3_) δ 8.89 (d, *J* = 14.0 Hz, 2H), 7.42 (d, *J* = 7.4 Hz, 2H), 7.38 (td, *J* = 7.6, 1.2 Hz, 2H), 7.24 (t, *J* = 7.4 Hz, 2H), 7.05 (d, *J* = 8.0 Hz, 2H), 6.04 (d, *J* = 14.1 Hz, 2H), 4.42 (q, *J* = 4.3 Hz, 1H), 3.99 (t, *J* = 7.6 Hz, 4H), 3.72 – 3.60 (m, 2H), 2.58 (t, *J* = 6.4 Hz, 4H), 1.95 (s, 5H), 1.88 – 1.83 (m, 4H), 1.82 (s, 12H), 1.52 – 1.38 (m, 8H), 1.37 – 1.24 (m, 16H), 0.91 (t, *J* = 6.6 Hz, 6H). LC-HRMS (ESI-TOF): calcd. for C_53_H_76_N_3_O_3_S^+^ ([M]^+^/2) 417.2801; found 417.2827.

^1^H NMR of IR-6B12Ac (400 MHz, CDCl_3_) δ 8.91 (d, *J* = 14.0 Hz, 2H), 7.46 – 7.33 (m, 4H), 7.24 (t, *J* = 7.5 Hz, 2H), 7.05 (d, *J* = 8.0 Hz, 2H), 6.03 (d, *J* = 14.0 Hz, 2H), 4.42 (d, *J* = 4.5 Hz, 1H), 3.98 (t, *J* = 7.6 Hz, 4H), 3.70 – 3.65 (m, 2H), 2.57 (d, *J* = 6.7 Hz, 4H), 1.95 (s, 5H), 1.88 – 1.83 (d, *J* = 6.1 Hz, 1H), 1.82 (s, 12H), 1.53 – 1.38 (m, 8H), 1.30 (d, *J* = 7.5 Hz, 28H), 0.91 (t, *J* = 6.7 Hz, 6H). MALDI-TOF-MS: calcd. for C_63_H_96_N_3_O_3_S^+^ [M]^+^ 918.6541; found 918.7242.

**Synthesis of FlavAc, FlavBu, and PhFlavAc.** The synthesis of Flav and PhFlav was based on our previous article. Dye (0.013 mmol) and acetylcysteine or 4-mercaptobutanoic acid (0.03 mmol) were dissolved in 1 mL DMSO. Then added 5 μL DIPEA and stirred at 70^o^C for 1 h. The crude product was purified by column chromatography on silica gel using DCM/MeOH. Flav and PhFlav were synthesized according to the previous report^2^.

^1^H NMR of FlavAc (400 MHz, MeOD) δ 8.38 – 8.23 (m, 1H), 7.82 (d, *J* = 9.2 Hz, 2H), 7.38 (t, *J* = 8.4 Hz, 2H), 6.95 – 6.83 (m, 4H), 6.79 – 6.66 (m, 2H), 6.62 (d, *J* = 8.7 Hz, 5H), 6.38 (s, 2H), 4.00 (d, *J* = 9.8 Hz, 1H), 3.73 (s, 12H), 3.36 (s, 8H), 3.23 (d, *J* = 6.9 Hz, 2H), 1.90 (s, 2H), 1.58 (s, 4H), 1.32 – 1.24 (m, 3H), 1.17 (t, *J* = 6.9 Hz, 12H). LC-HRMS (ESI-TOF): calcd. for C_57_H_64_N_3_O_9_S^+^ ([M+H]^+^/2) 483.7216; found 483.7233.

^1^H NMR of FlavBu (400 MHz, CDCl_3_) δ 8.56 (d, *J* = 13.6 Hz, 2H), 7.43 (t, *J* = 8.5 Hz, 2H), 7.03 – 6.90 (m, 6H), 6.73 – 6.64 (m, 6H), 6.51 (s, 2H), 3.83 (s, 12H), 3.57 – 3.44 (m, 8H), 2.74 (s, 4H), 2.56 (d, *J* = 6.8 Hz, 1H), 1.93 (s, 2H), 1.57 (s, 12H), 1.42 (d, *J* = 1.9 Hz, 2H), 0.94 (d, *J* = 6.6 Hz, 6H). LC-HRMS (ESI-TOF): calcd. For C_56_H_65_N_2_O_6_S^+^ ([M]^+^/2) 446.7279 ; found 446.7298.

^1^H NMR of PhFlavAc (400 MHz,CDCl_3_) δ 8.56 (d, *J* = 13.7 Hz, 2H), 7.88 (d, *J* = 9.4 Hz, 2H), 7.35 (dd, *J* = 8.8, 6.8 Hz, 10H), 7.22 – 7.15 (m, 14H), 7.04 (dd, *J* = 9.1, 2.4 Hz, 2H), 6.96 (d, *J* = 14.1 Hz, 2H), 6.80 (d, *J* = 2.3 Hz, 2H), 6.60 (d, *J* = 8.4 Hz, 4H), 4.38 (d, *J* = 5.8 Hz, 1H), 3.78 (s, 12H), 3.28 (ddd, *J* = 50.7, 14.0, 5.6 Hz, 2H), 2.67 (d, *J* = 6.4 Hz, 4H), 2.60 (s, 6H), 1.87 (s, 2H), 1.74 (s, 3H). MALDI-TOF-MS: calcd. for C_73_H_64_N_3_O_9_S^+^ [M]^+^ 1158.4358; found 1158.4430.

**2. Synthesis of FlavRGD**


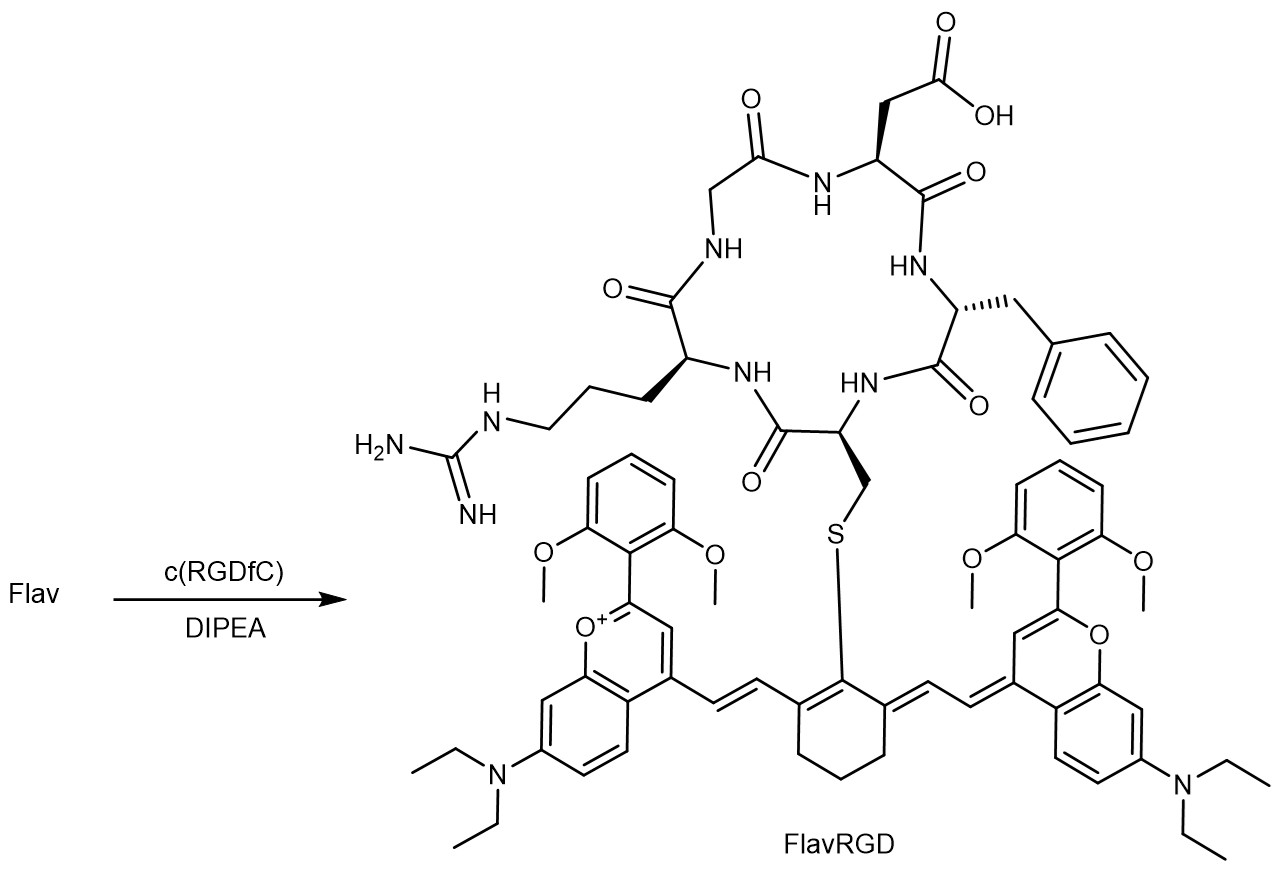


**Scheme S2.** General synthetic route of FlavRGD.

Flav (0.013 mmol) and c(RGDfC) (0.03 mmol) were dissolved in 1 mL DMSO. Then added 5 μL DIPEA and stirred at 70^o^C for 1 h. The crude product was purified by column chromatography on silica gel using DCM/MeOH.

^1^H NMR of FlavRGD (400 MHz, CDCl_3_) δ 8.58 (s, 2H), 8.38 (s, 1H), 7.77 (d, *J* = 9.3 Hz, 2H), 7.40 (t, *J* = 8.5 Hz, 2H), 6.94 (t, *J* = 8.3 Hz, 6H), 6.83 (d, *J* = 13.7 Hz, 2H), 6.77 (d, *J* = 9.6 Hz, 2H), 6.65 (d, *J* = 8.5 Hz, 4H), 6.47 (s, 2H), 4.81 (s, 1H), 4.50 (s, 1H), 4.26 (s, 1H), 3.81 (s, 1H), 3.78 (s, 12H), 3.55 – 3.38 (m, 8H), 2.67 (s, 4H), 1.89 (s, 2H), 1.68 – 1.58 (m, 2H), 1.33 – 1.24 (m, 112H), 1.22 (d, *J* = 7.1 Hz, 5H). MALDI-TOF-MS: calcd. for C_76_H_89_N_10_O_13_S^+^ [M]^+^ 1381.6326; found 1381.5212.

**3. Synthesis of Flavn**


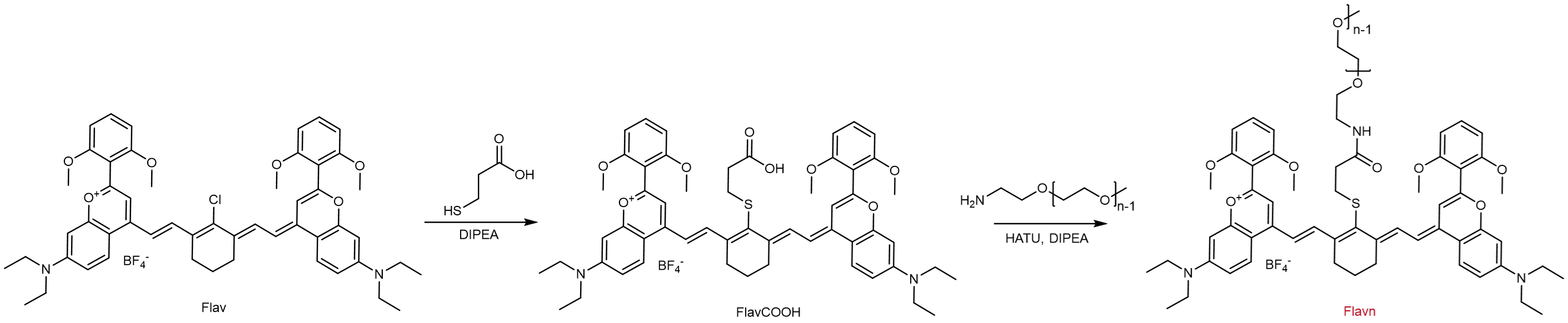


**Scheme S3.** General synthetic route of Flavn.

**Synthesis of FlavCOOH.** The 3-sulfanylpropanoic acid (3 mmol), and DIPEA (522 μL, 3 mmol) were added to a flame-dried flask and dissolved in anhydrous DMSO (0.1 M). Dye (1 mmol) was subsequently added to the mixture with vigorous stirring and heated to 70°C for 1 h under a protection gas atmosphere. The crude product was purified by column chromatography on silica gel using DCM/MeOH.

^1^H NMR of FlavCOOH (400 MHz, CDCl_3_) δ 8.52 (d, *J* = 13.8 Hz, 2H), 7.97 (d, *J* = 9.5 Hz, 2H), 7.43 (t, *J* = 8.5 Hz, 2H), 7.03 – 6.91 (m, 6H), 6.69 (d, *J* = 8.5 Hz, 4H), 6.51 (d, *J* = 2.6 Hz, 2H), 3.83 (s, 12H), 3.50 (q, *J* = 7.1 Hz, 8H), 2.89 (t, *J* = 6.4 Hz, 2H), 2.76 (s, 4H), 2.52 (t, *J* = 6.4 Hz, 2H), 1.94 (t, *J* = 6.1 Hz, 2H), 1.25 (t, *J* = 7.1 Hz, 12H).

**Synthesis of Flav2, Flav3, Flav7, Flav9, Flav12, and Flav45.** The FlavCOOH (1 mmol), and 2-(7-Azabenzotriazol-1-yl)-N,N,N',N'-tetramethyluronium hexafluorophosphate (1 mmol) was added to a flame-dried flask and dissolved in anhydrous THF (0.1 M) and the mixture was stirred at room temperature for 30 min. mPEG2000-NH_2_ (for Flav45); mPEG550-NH_2_ (for Flav12); 2-(2-Methoxyethoxy)ethanamine (for Flav2); 2-(2-(2-Methoxyethoxy)ethoxy)ethanamine (for Flav3); mPEG7-Amine (for Flav7); mPEG9-Amine (for Flav9, 1.1 mmol) and DIPEA (2 mmol) were subsequently added to the mixture and then stirred for 12 h under a protection gas atmosphere. The reaction was monitored by TLC using MeOH/DCM (20:1) as the solvent system. The crude product was purified by column chromatography on silica gel using DCM/MeOH.

^1^H NMR of Flav2 (400 MHz, CDCl_3_) δ 8.65 – 8.53 (m, 1H), 8.01 (d, *J* = 9.3 Hz, 2H), 7.44 (t, J = 8.3 Hz, 3H), 7.13 – 6.80 (m, 6H), 6.70 (dd, *J* = 8.5, 2.1 Hz, 4H), 6.54 (s, 2H), 3.86 (d, *J* = 2.1 Hz, 12H), 3.56 – 3.49 (m, 8H), 3.49 – 3.44 (m, 6H), 3.33 (s, 3H), 3.30 – 3.24 (m, *J* = 6.1 Hz, 2H), 3.01 (t, *J* = 7.4 Hz, 2H), 2.90 – 2.75 (s, 4H), 2.49 (t, *J* = 7.3 Hz, 2H), 2.00 (s, 2H), 1.29 (d, *J* = 7.2 Hz, 12H). LC-HRMS (ESI-TOF): calcd. For C_60_H_73_N_3_O_9_S^+^ ([M+H]^+^/2) 505.7529; found 505.7524.

^1^H NMR of Flav3 (400 MHz, CDCl_3_) δ 8.59 (d, *J* = 13.6 Hz, 2H), 8.02 (d, *J* = 9.4 Hz, 2H), 7.54 – 7.41 (m, 3H), 7.10 – 6.92 (m, 6H), 6.71 (d, *J* = 8.7 Hz, 4H), 6.55 (s, 2H), 3.87 (s, 12H), 3.69 (s, 2H), 3.64 – 3.57 (m, 6H), 3.55 – 3.50 (m, 8H), 3.48 – 3.43 (m, 2H), 3.36 (s, 3H), 3.30 – 3.23 (m, 2H), 3.06 – 2.99 (m, 2H), 2.82 (s, 4H), 2.50 (s, 2H), 2.10 – 1.95 (m, 2H), 1.29 (t, *J* = 6.8 Hz, 12H). MALDI-TOF-MS: calcd. for C_62_H_76_N_3_O_10_S^+^ ([M+H]^+^) 1055.5324; found 1055.4374.

^1^H NMR of Flav7 (400 MHz, CDCl_3_) δ 8.59 (d, *J* = 13.7 Hz, 2H), 8.01 (d, *J* = 9.4 Hz, 2H), 7.55 (s, 1H), 7.45 (t, *J* = 8.3 Hz, 2H), 7.09 – 7.90 (m, 6H), 6.70 (d, *J* = 8.8 Hz, 4H), 6.55 (s, 2H), 3.87 (s, 12H), 3.70 – 3.60 (m, 24H), 3.55 – 3.50 (m, 8H), 3.47 – 3.43 (m, 2H), 3.40 (s, 3H), 3.26 (d, *J* = 6.1 Hz, 2H), 3.02 (t, *J* = 7.4 Hz, 2H), 2.85 – 2.78 (m, 4H), 2.50 (t, *J* = 7.4 Hz, 2H), 2.04 –1.96 (m, 2H), 1.32 – 1.25 (m, 12H). MALDI-TOF-MS: calcd. for C_70_H_93_N_3_O_14_S^+^ ([M+H]^+^) 1231.6373; found 1231.3808.

^1^H NMR of Flav9 (400 MHz, CDCl_3_) δ 8.58 (d, *J* = 13.7 Hz, 2H), 8.02 (t, *J* = 8.5 Hz, 2H), 7.53 – 7.37 (m, 3H), 7.07 – 6.90 (m, 6H), 6.71 (t, *J* = 8.9 Hz, 4H), 6.55 (d, *J* = 5.9 Hz, 2H), 3.87 (d, *J* = 2.5 Hz, 12H), 3.67 (d, *J* = 6.1 Hz, 32H), 3.58 (q, *J* = 2.9 Hz, 14), 3.53 (d, *J* = 7.3 Hz, 8H), 3.41 (d, *J* = 3.2 Hz, 3H), 3.29 – 3.17 (m, 2H), 2.80 (s, 4H), 2.58 – 2.46 (m, 2H), 2.00 (s, 2H), 1.29 (d, *J* = 6.9 Hz, 12H). MALDI-TOF-MS: calcd. for C_74_H_101_N_3_O_16_S^+^ ([M+H]^+^) 1319.6897; found 1319.8079.

^1^H NMR of Flav12 (400 MHz, CDCl_3_) δ 8.54 (d, *J* = 13.7 Hz, 2H), 7.97 (d, *J* = 9.4 Hz, 2H), 7.40 (t, *J* = 8.5 Hz, 2H), 7.01 (s, 2H), 6.95 (d, *J* = 13.8 Hz, 2H), 6.89 (dd, *J* = 9.3, 2.6 Hz, 2H), 6.66 (d, *J* = 8.5 Hz, 4H), 6.49 (d, *J* = 2.6 Hz, 2H), 3.82 (s, 12H), 3.65 – 3.61 (m, 44H), 3.55 – 3.51 (m, 4H), 3.50 – 3.45 (m, 8H), 3.36 (s, 3H), 2.97 (t, *J* = 7.4 Hz, 2H), 2.77 (t, *J* = 6.2 Hz, 4H), 2.44 (t, *J* = 7.3 Hz, 2H), 2.01 – 1.92 (m, 2H), 1.24 (t, *J* = 7.1 Hz, 12H).

^1^H NMR of Flav45 (400 MHz, CDCl_3_) δ 9.09 – 7.86 (m, 4H), 7.65 – 7.41 (m, 6H), 6.76 – 6.63 (m, 8H), 3.91 – 3.84 (m, 12H), 3.68 (s, 176H), 3.60 – 3.56 (m, 4H), 3.51 (d, *J* = 5.2 Hz, 8H), 3.41 (s, 3H), 3.27 – 3.21 (m, 2H), 3.13 (dd, *J* = 7.5, 4.4 Hz, 4H), 2.46 – 2.33 (m, 2H), 2.05 (s, 2H), 1.29 (d, *J* = 5.9 Hz, 12H).

^13^C NMR of Flav45 (101 MHz, DMSO-*d_6_*) δ 170.32, 158.92, 157.27, 154.44, 152.46, 151.56, 144.74, 140.84, 136.97, 133.08, 130.10, 126.97, 113.71, 112.01, 111.90, 111.32, 108.33, 104.88, 71.75, 70.25, 70.11, 70.05, 69.92, 60.48, 58.52, 56.57, 53.91, 44.92, 42.17, 38.95, 27.03, 18.50, 17.19, 12.93, 12.80.

**Supplementary Figures and Tables**

**
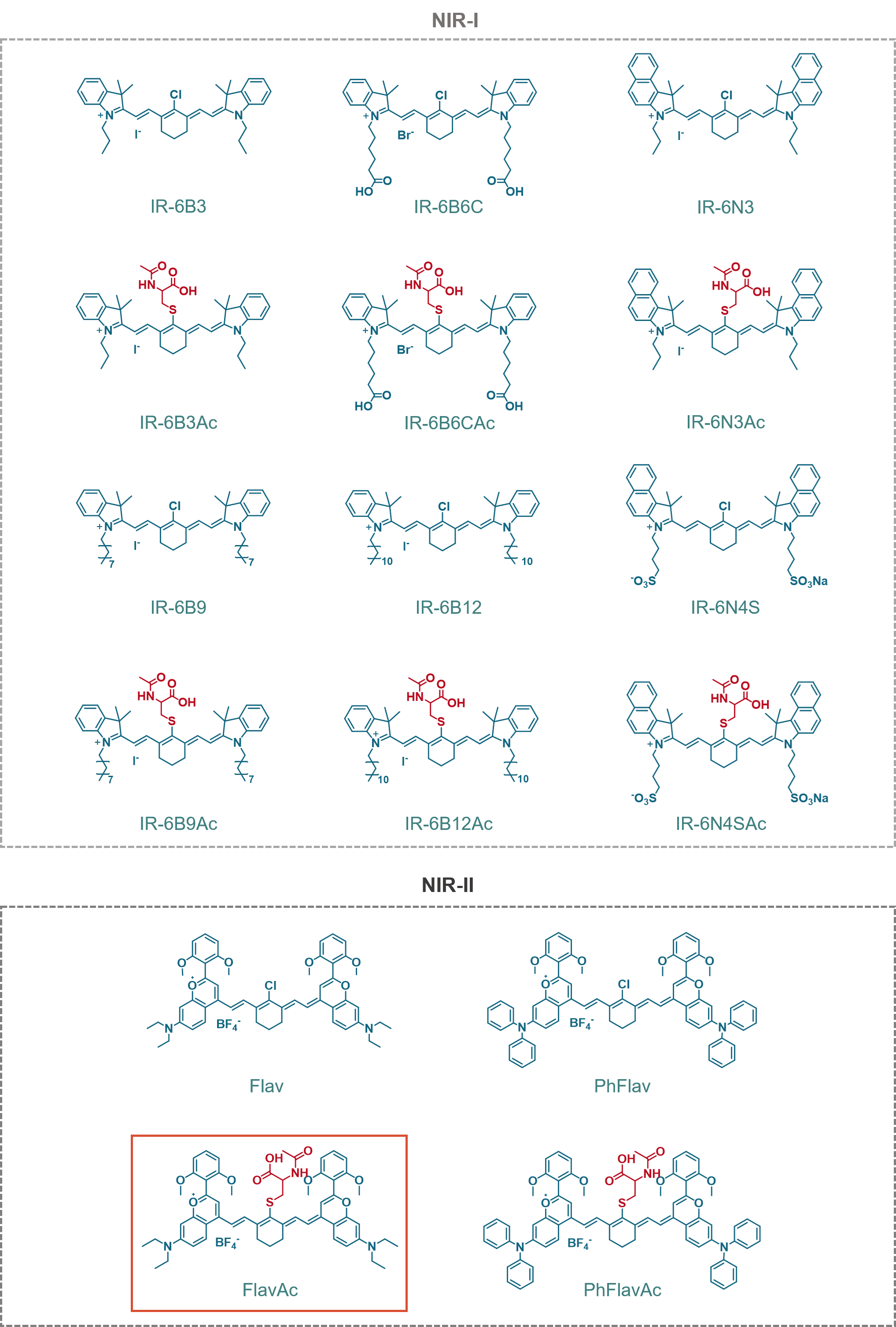
**

**Figure S1.** Chemical structures of dyes used in figure 1b. The tail emission of NIR-I dyes can be used for NIR-II imaging.

**
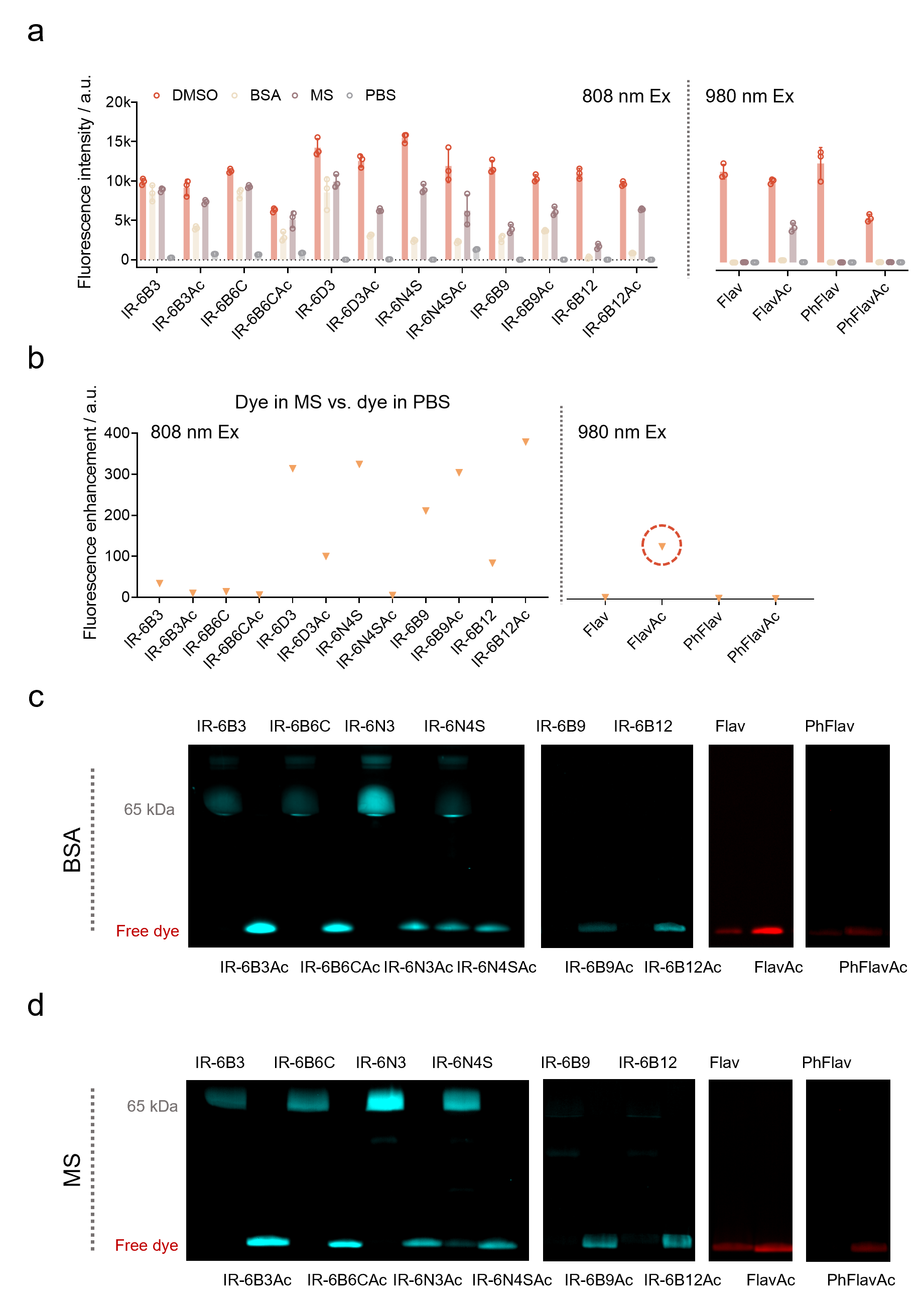
**

**Figure S2.** (a) NIR-II fluorescence intensity of dyes in DMSO, bovine serum albumin (BSA), mouse serum (MS), and PBS 2 h after co-incubation at 50^o^C (n=3). Dye concentration: 10 μM; BSA concentration: 50 mg mL^-1^. (b) Brightness enhanced folds of dye in MS versus those in PBS. (c-d) NIR-II imaging of the gel after separating the mixture in a by gel electrophoresis. Diluted the BSA solution and MS 5 times when loading the samples.

**
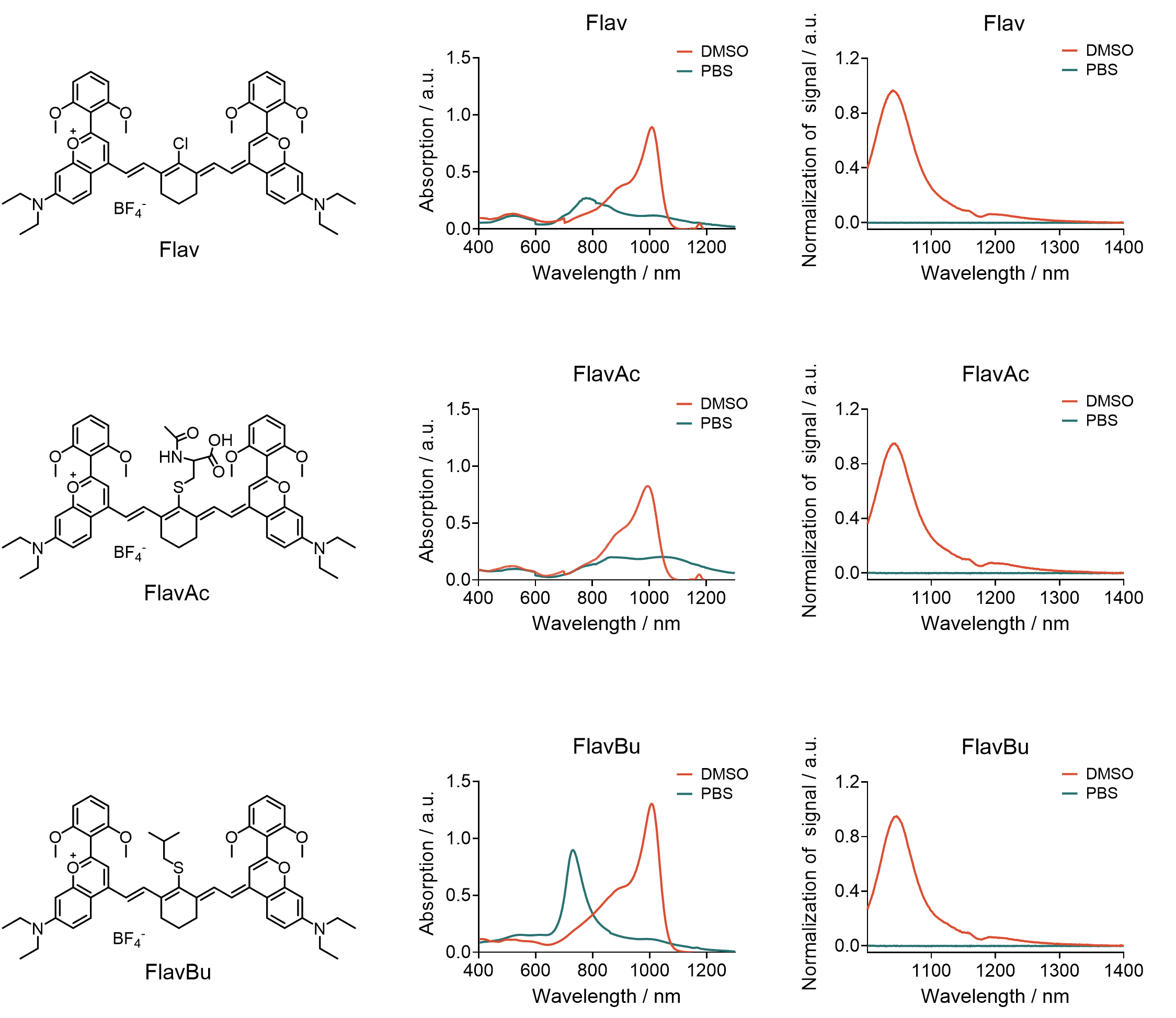
**

**Figure S3****.** Chemical structure, absorption and fluorescence spectra of Flav, FlavAc, and FlavBu in DMSO and PBS respectively. Concentration of dyes: 10 μM; excited with 980 nm laser.

**
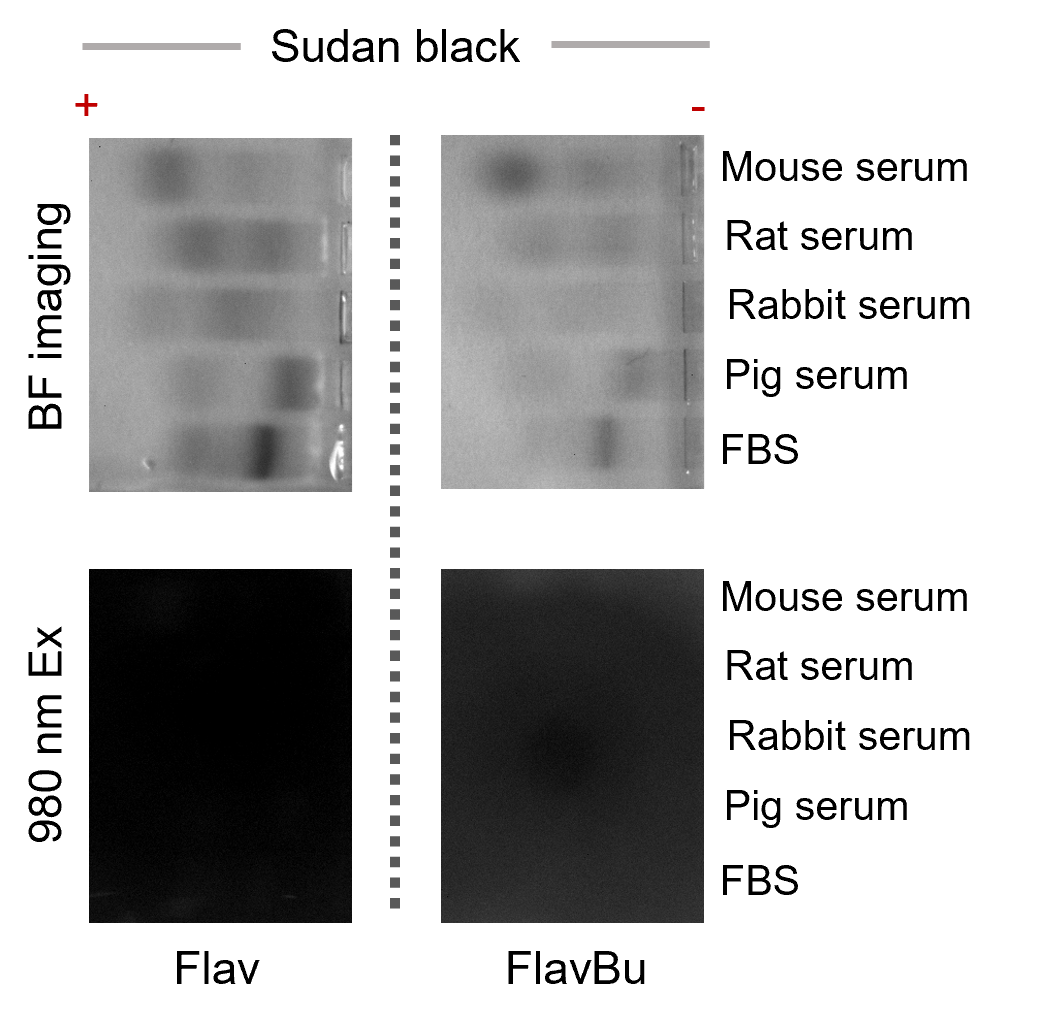
**

**Figure S4.** Agarose gel electrophoresis of Flav and FlavBu in serum from different species. BF: bright field; 980 nm Ex: excited with 980 nm laser; FBS: fetal bovine serum.


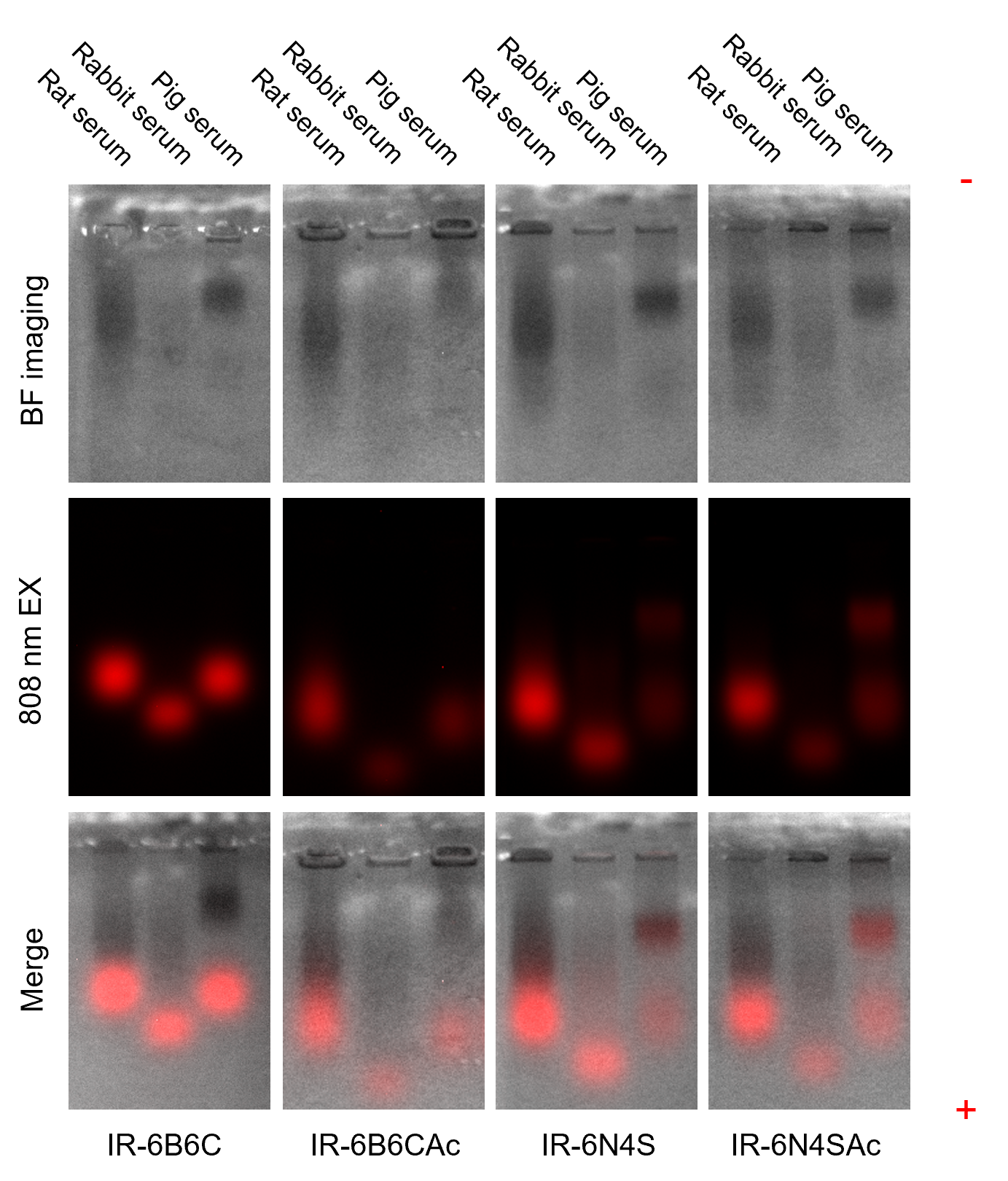


**Figure S5.** Agarose gel electrophoresis of IR-6B6C, IR-6B6CAc, IR-6N4S, and IR-6N4SAc in serum from different species.

**
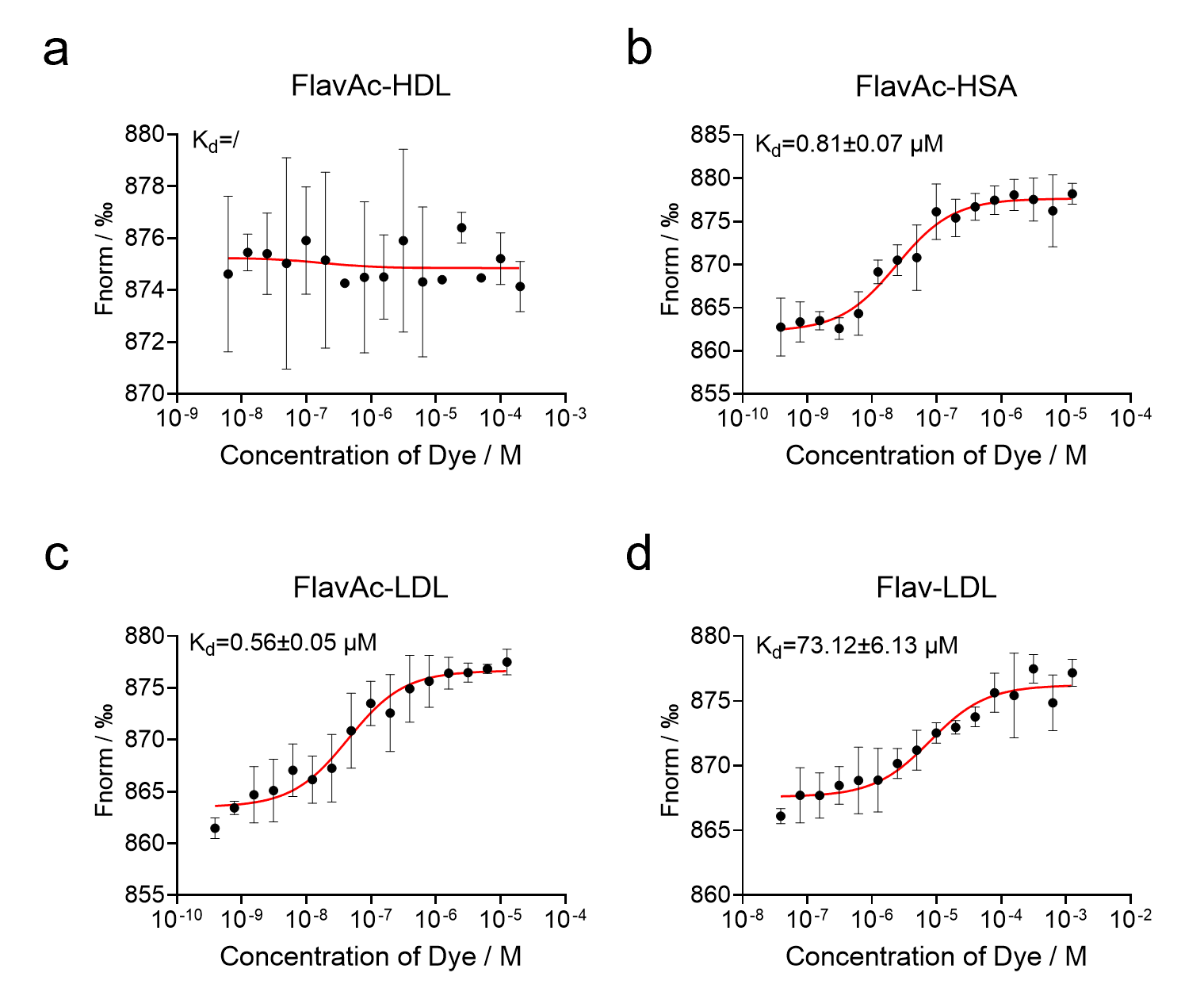
**

**Figure S6.** (a-c) Binding affinity of FlavAc for different components measured by Microscale thermophoresis (MST, n=3). (d) Binding affinity of Flav with low-density lipoprotein (LDL) measured by MST. HSA: human serum albumin; HDL: high-density lipoprotein. Due to the weak fluorescence of FlavAc in albumin solution, we speculated that the interaction between HSA and FlavAc didn’t alter its aggregation-caused quenching behavior in water, and the fluorescence enhancement was specifically generated by LDL.

**Note:** The above data was acquired with same concentration gradient of molecule and same concentration of biomacromolecule (200 μg mL^-1^). It should be noted that variations in experimental conditions or methodologies can alter the measured Kd values.


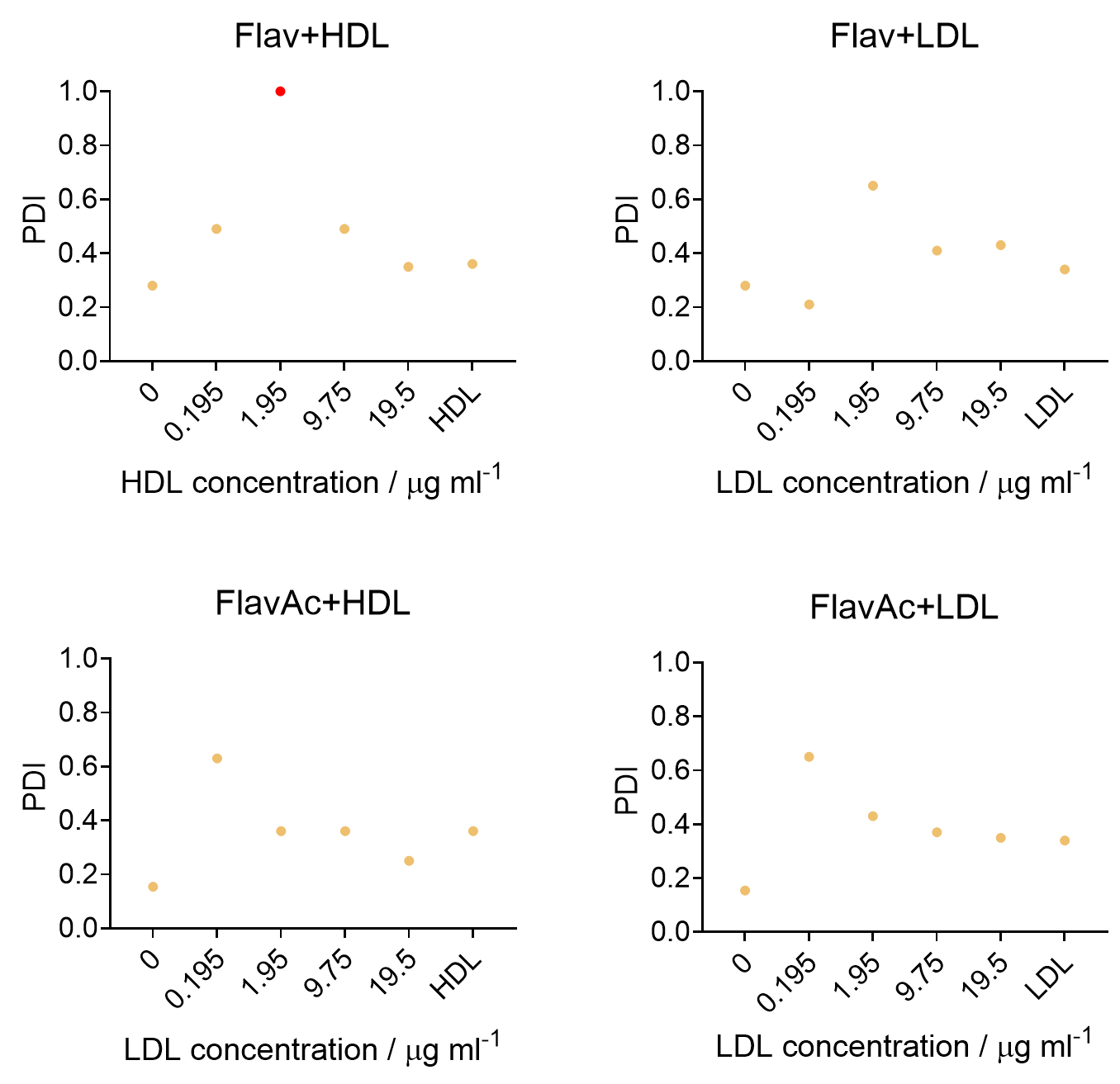


**Figure S7.** Statistical analysis of the average PDI from figure 2e-g.


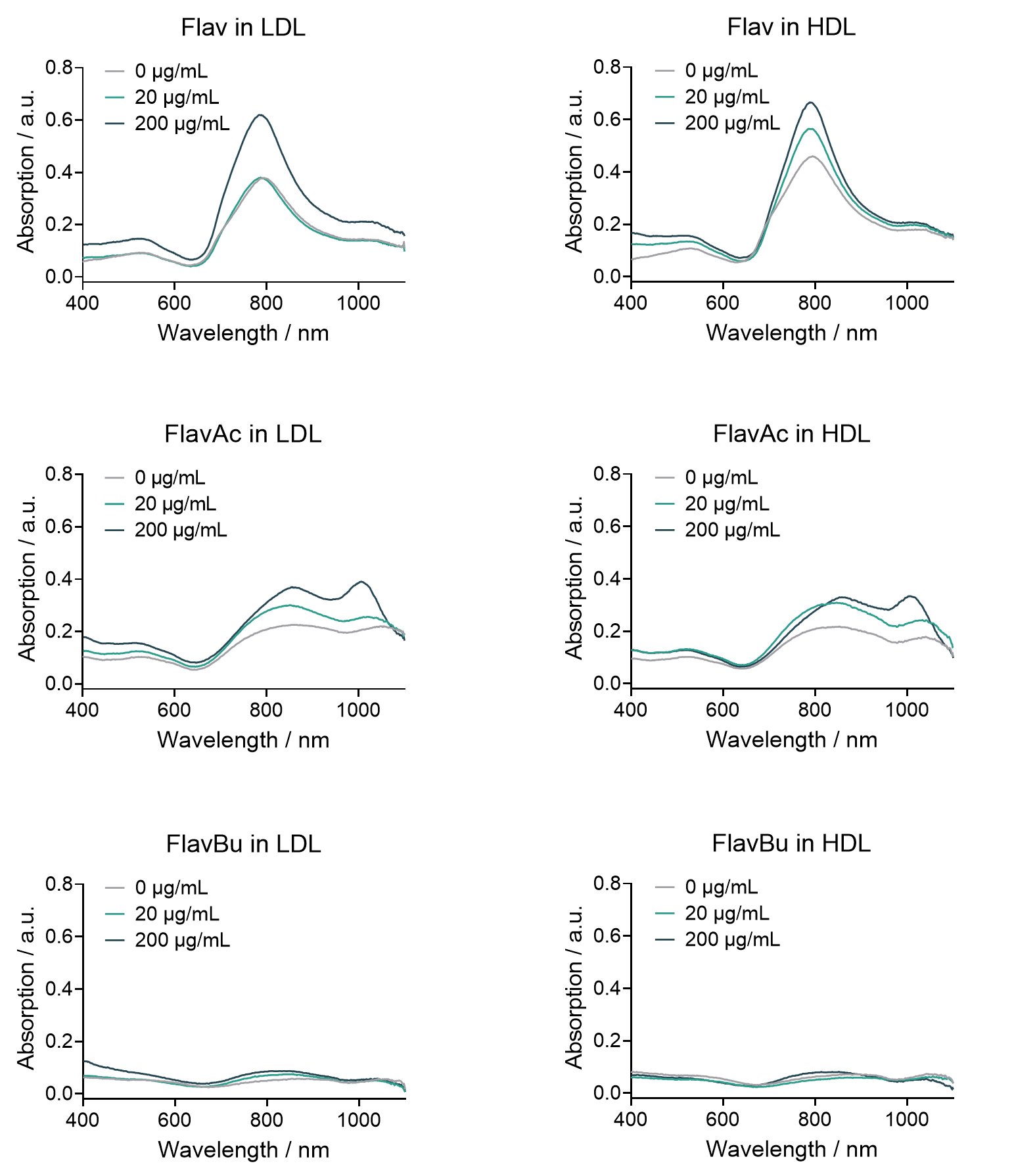


**Figure S8.** Absorption spectra of Flav, FlavAc, and FlavBu in different concentrations of lipoproteins after incubation at 50°C for 30 min.


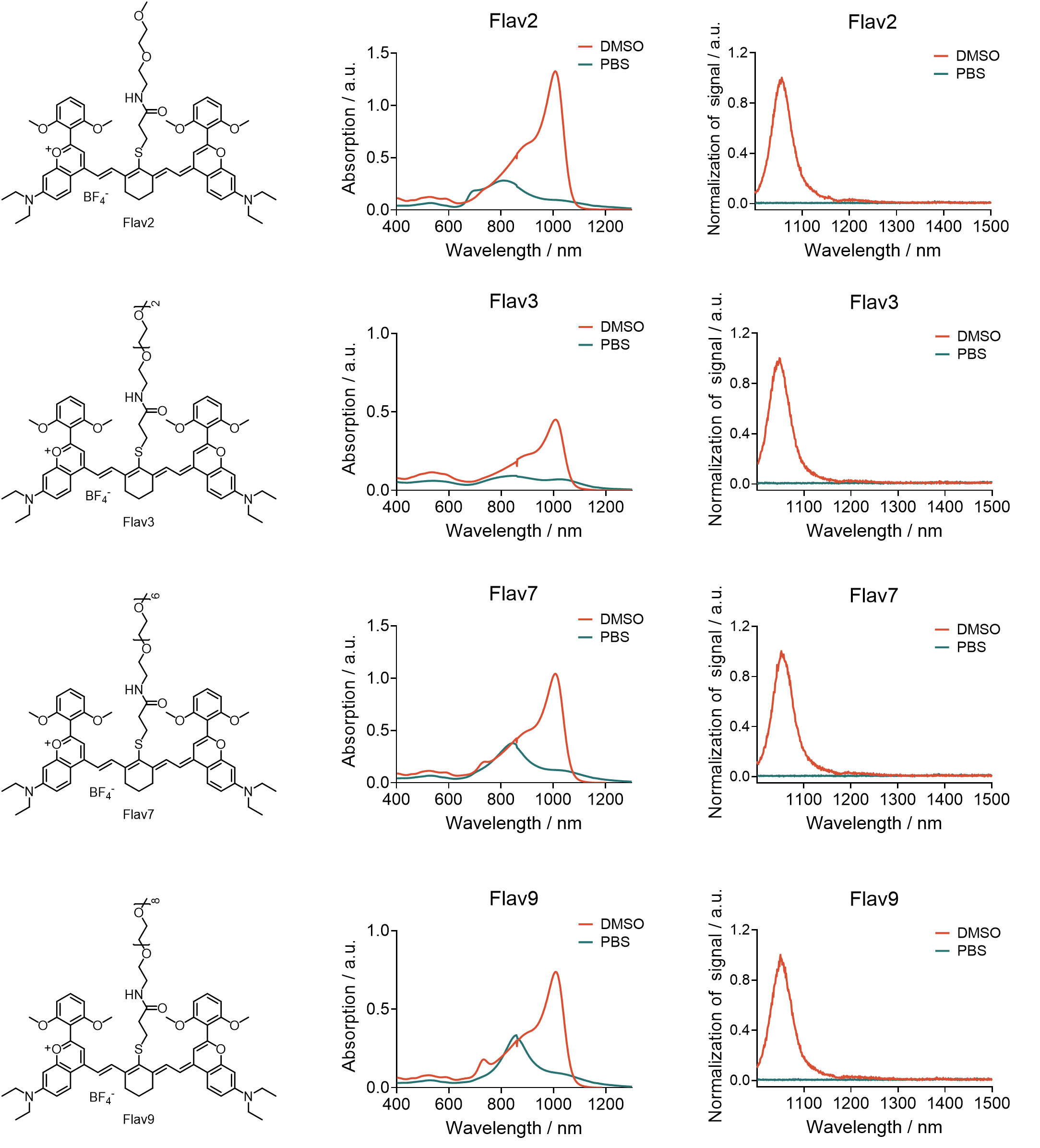


**Figure S9.** Molecular structure, absorption and fluorescence spectra of Flav2, Flav3, Flav7, and Flav9 in DMSO and PBS respectively. Excited by 980 nm laser.


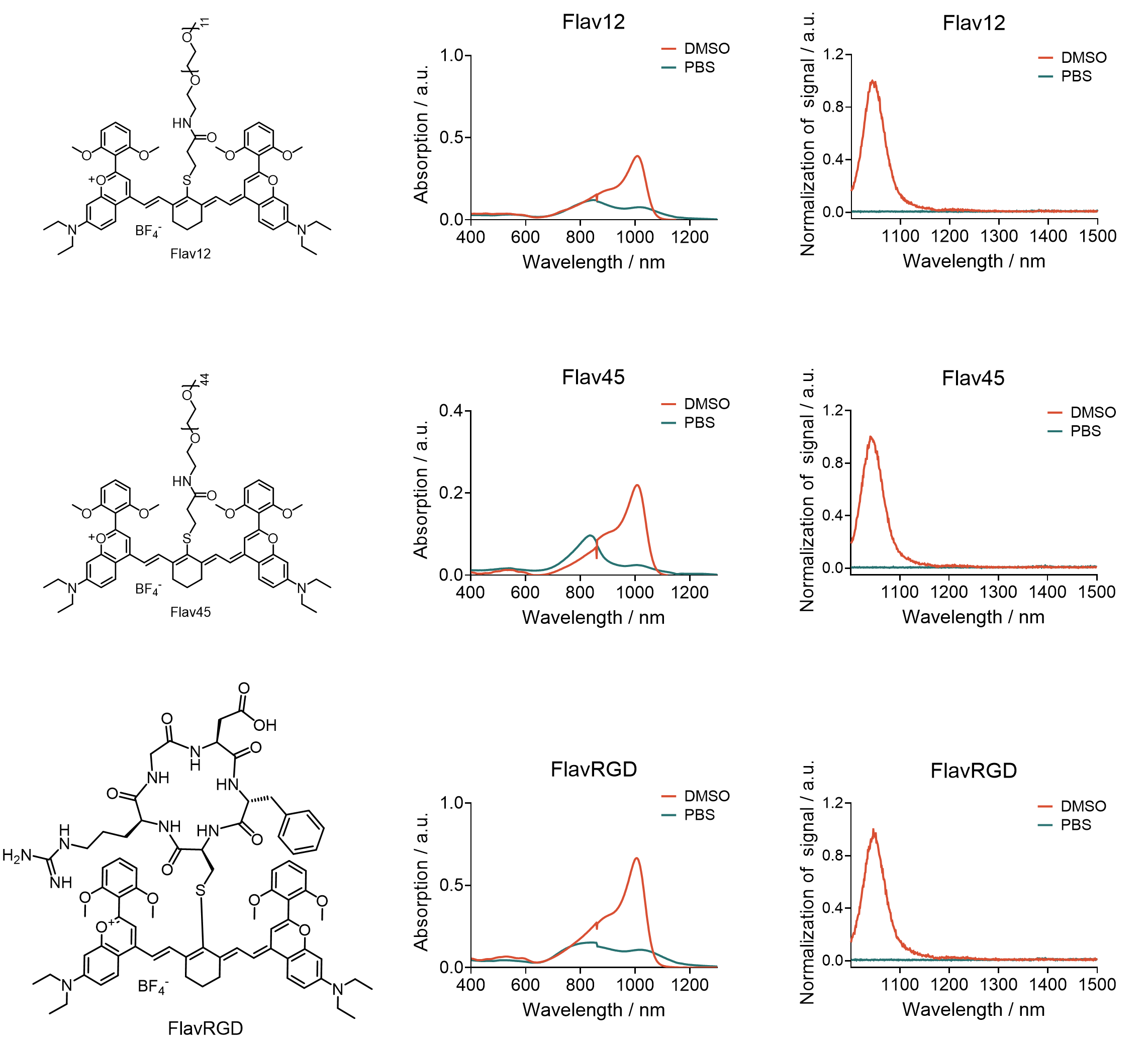


**Figure S10.** Molecular structure, absorption and fluorescence spectra of Flav12, Flav45, and FlavRGD in DMSO and PBS respectively. Excited by 980 nm laser.


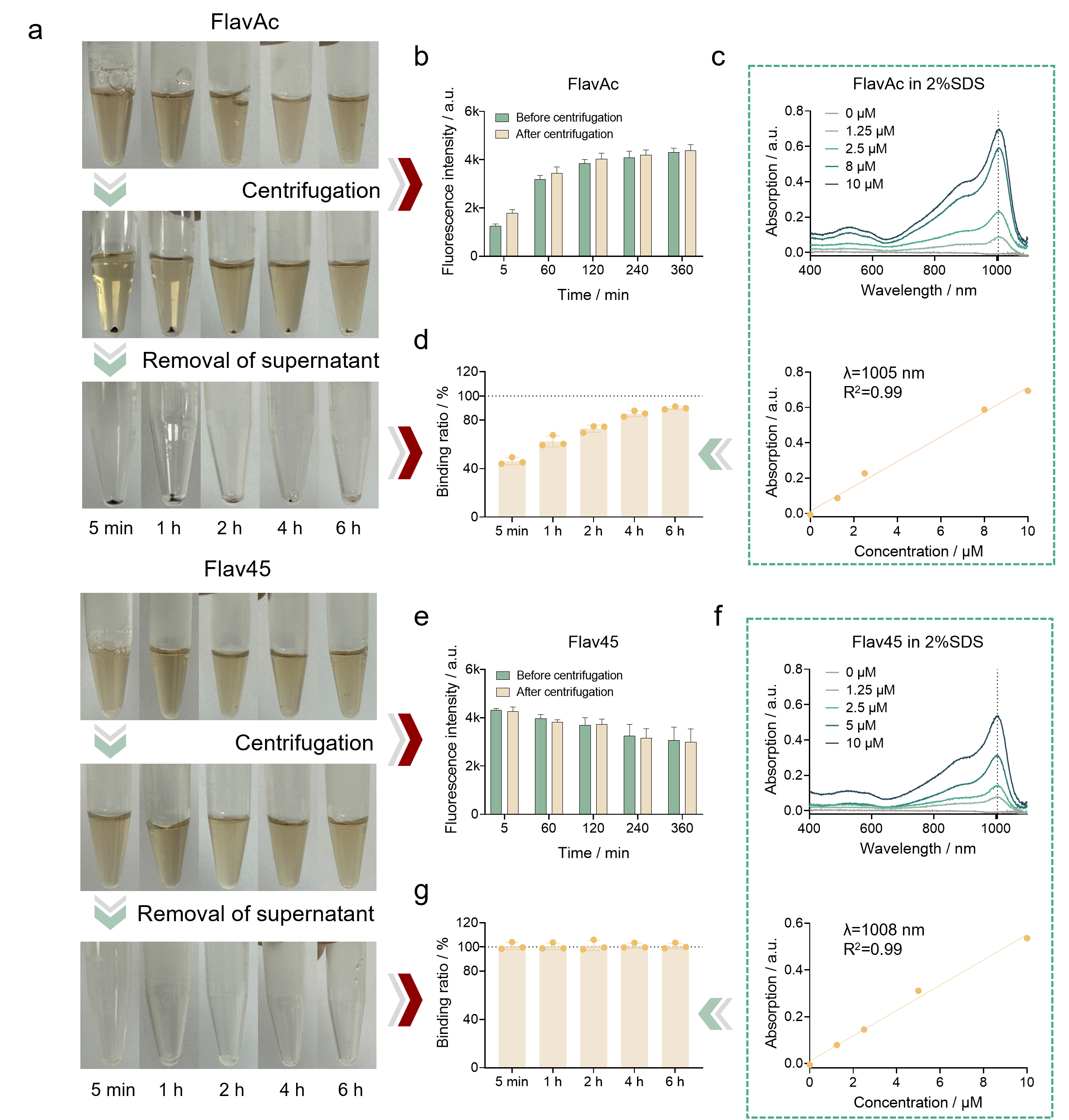


**Figure S11.​​** (a) Photographs of FlavAc and Flav45 after incubation with FBS at different time points, followed by centrifugation to separate the aggregates of dye without combining with lipoproteins. (b) Statistics of supernatant brightness before and after centrifugation for the mixtures of Flav45 and FBS incubated for different durations (corresponding to images in a, n=3). (c) Absorption spectra of FlavAc at different concentrations in 2% (w/v) SDS solution and standard curve of absorbance versus concentration at 1005 nm for FlavAc in 2% (w/v) SDS solution. (d) Percentage of binding dye relative to the total added dye after incubation for different time periods (for FlavAc). (e) Statistics of supernatant brightness before and after centrifugation for the mixtures of Flav45 and FBS incubated for different durations (corresponding to images in a, n=3). (f) Absorption spectra of Flav45 at different concentrations in 2% (w/v) SDS solution. and standard curve of absorbance versus concentration at 1008 nm for Flav45 in 2% (w/v) SDS solution. (g) Percentage of binding dye relative to the total added dye after incubation for different time periods (for Flav45).


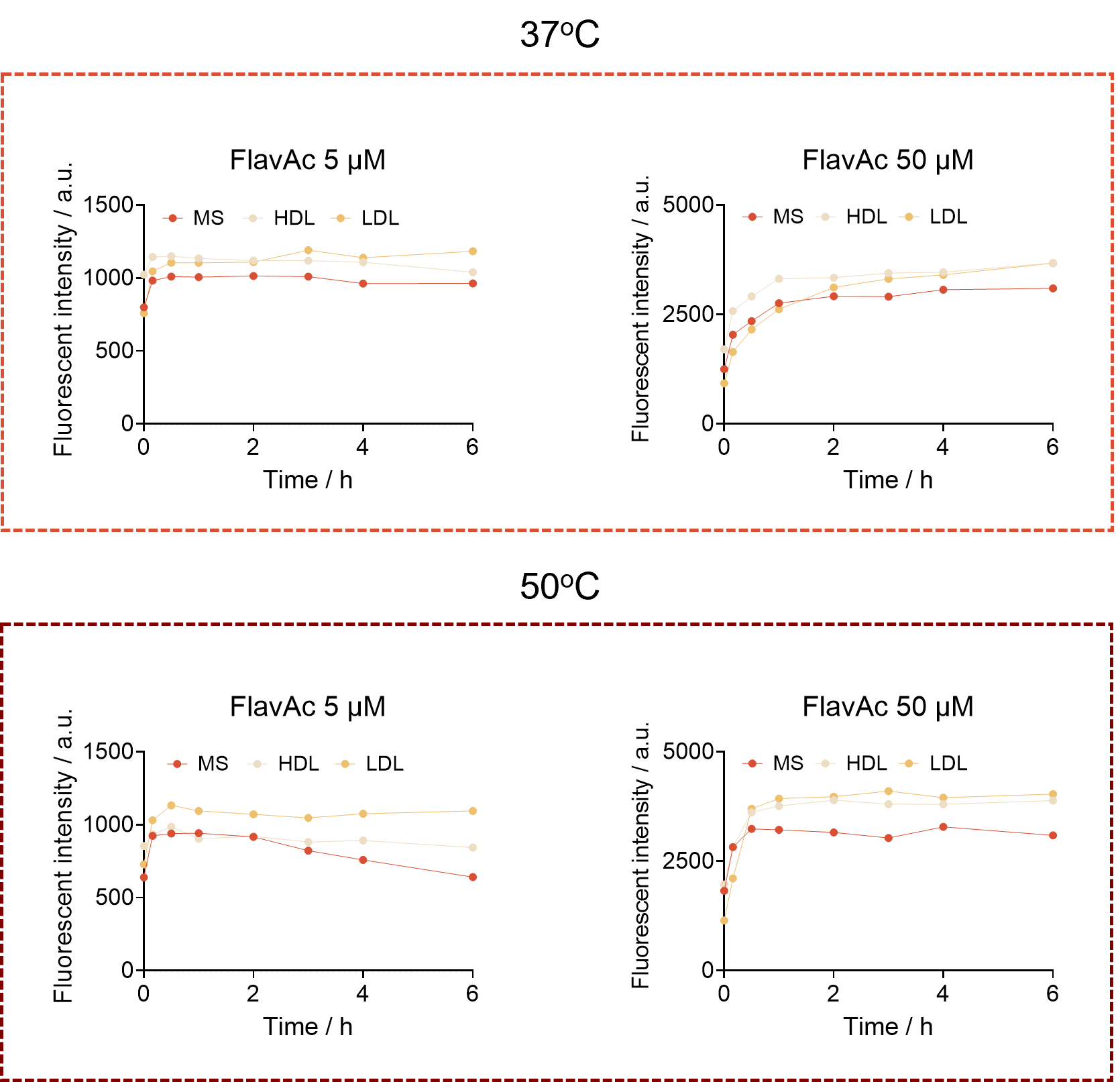


**Figure S12.** NIR-II fluorescence change of FlavAc at different concentration in MS, HDL, and LDL; incubated at 37^o^C and 50^o^C.

**
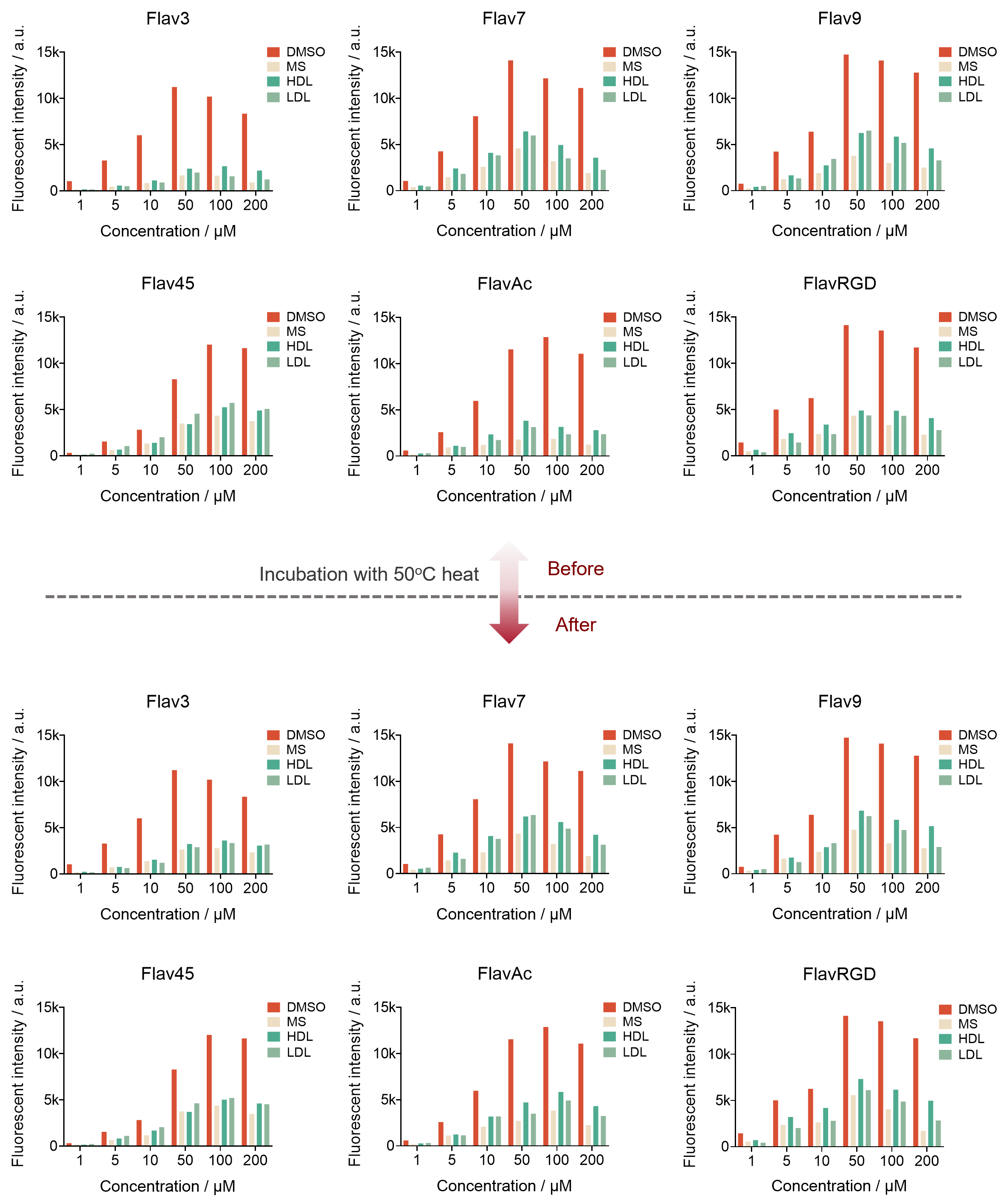
**

**Figure S13.** NIR-II fluorescence intensity of dyes at different concentrations in DMSO, MS, HDL, and LDL after and before the co-incubation at 50^o^C for 2 h.

**
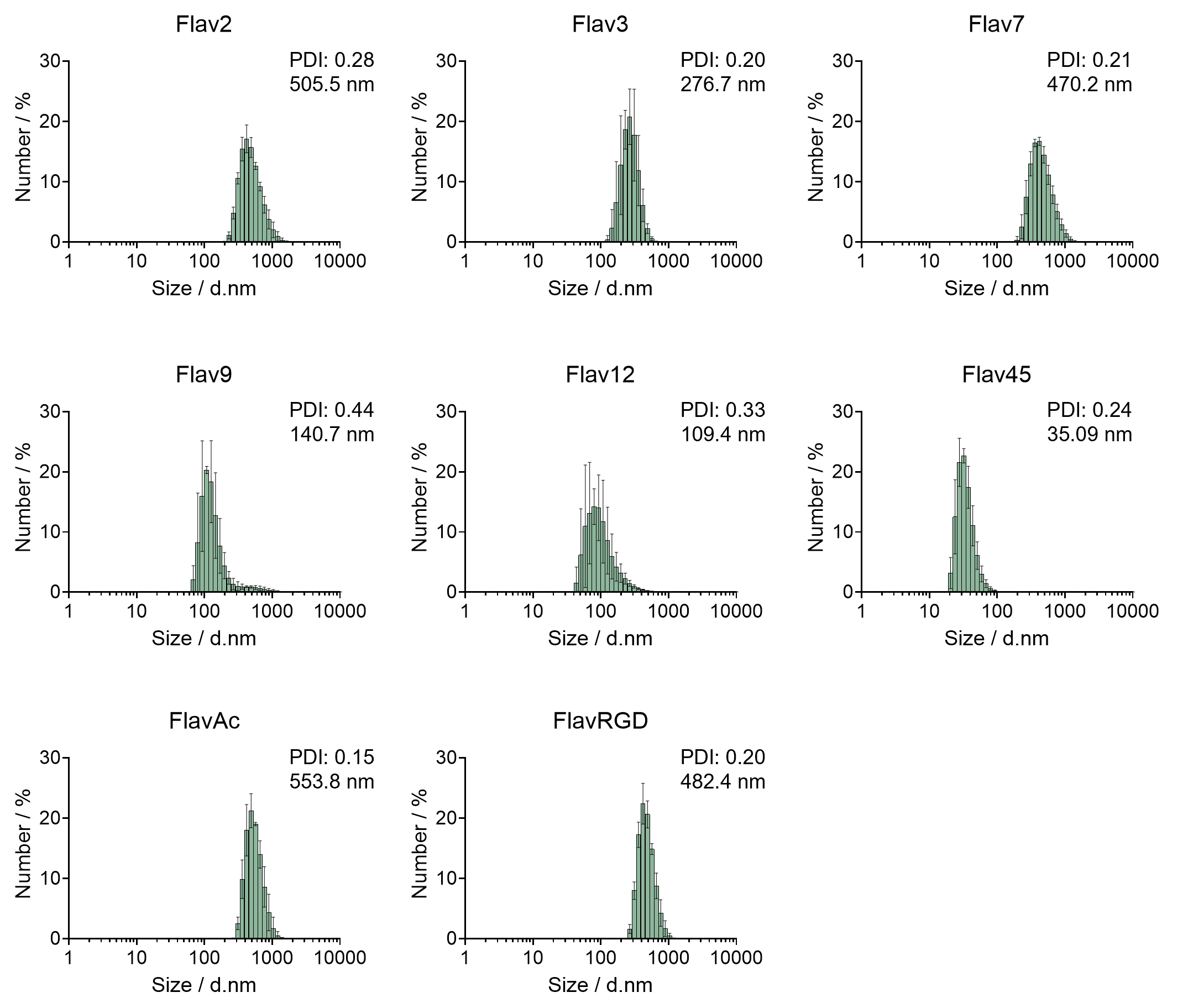
**

**Figure S14.** Particle size distribution of Flav2, Flav3, Flav7, Flav9, Flav12, Flav45, FlavAc, and FlavRGD in PBS (n=3). Concentration of dyes: 1 μM.


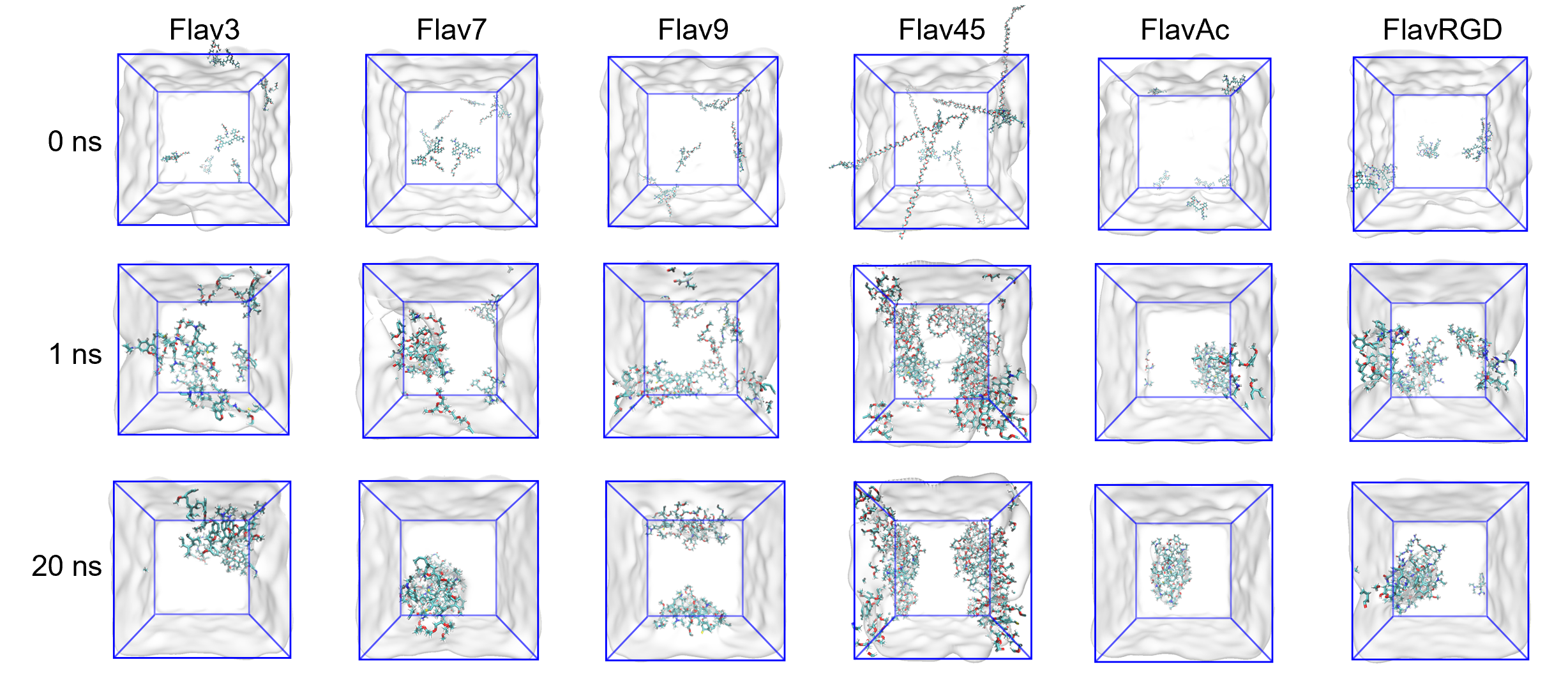


**Figure S15.** The molecular dynamics simulations process of different dyes in aqueous solution.

**
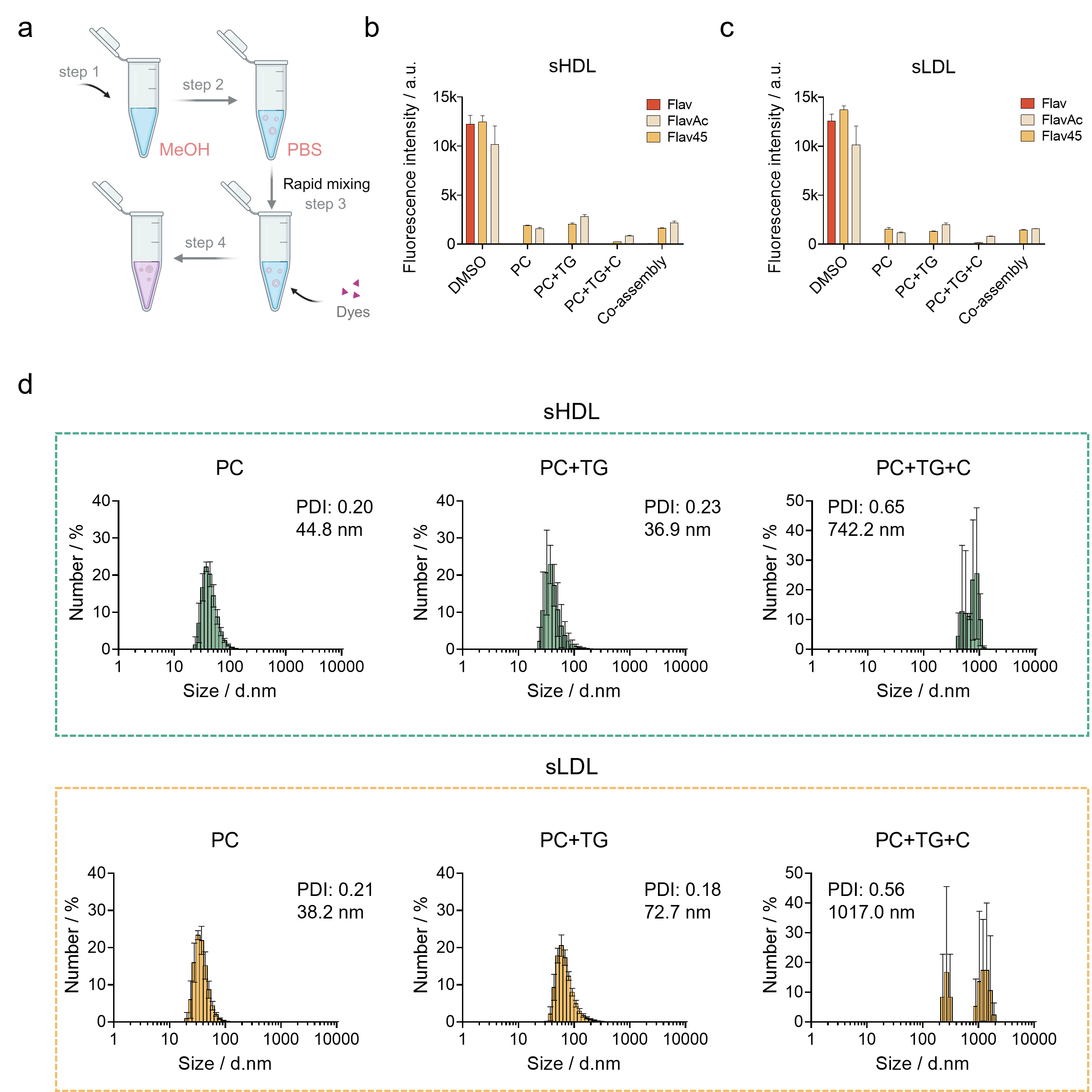
**

**Figure S16.** (a) Preparation process of synthetic lipoprotein coated dye. (b-c) NIR-II fluorescence intensity of dyes by adding sHDL (b) and sLDL (c) with different components, compared with in DMSO and co-assembly group (n=3). (d) Particle size distribution of sHDL and sLDL with different components (n=3). sHDL: Synthetic high-density lipoprotein; sLDL: Synthetic low-density lipoprotein; PC: phosphatidyl choline; TG: triglyceride; C: cholesterol. Due to its poor size homogeneity and excessively large average size, the “PC+TG+C” groups were poor representatives for lipoprotein properties.

**Note:** The synthesis method of synthetic lipoprotein was as follows. Step 1: The stocks were prepared according to natural lipoprotein component in MeOH (sHDL group: the stocks of “PC” was composed of 9.2 mg mL^-1^ PC; the stocks of “PC+TG” was composed of 9.2 mg mL^-1^ PC and 1.15 mg mL^-1^ TG; the stocks of “PC+TG+C” was composed of 9.2 mg mL^-1^ PC, 1.15 mg mL^-1^ TG, and 9.6 mg mL^-1^ C; sLDL group: the stocks of “PC” was composed of 5 mg mL^-1^ PC; the stocks of “PC+TG” was composed of 5 mg mL^-1^ PC and 2.5 mg mL^-1^ TG; the stocks of “PC+TG+C” was composed of 5 mg mL^-1^ PC, 2.5 mg mL^-1^ TG, and 12.5 mg mL^-1^ C respectively);^3^ Step 2-3: 30 μL PBS was added into EP tube, 10 μL stocks was added, then 90 μL PBS was added and the mixture was ​​pipetted up and down ten times to thoroughly mix; Step 4: All mixtures were sonicated in an ice-water bath for 30 min. After the formation of stable nanoparticles was observed, 10 μL was added into 8-tube strips and 0.1 μL (1 mM in DMSO) dye was added. Co-assembly group: 1.4 μL dye and 10 μL lipoprotein components were first mixed. The resulting mixture was then added to 130 μL PBS and sonicated in an ice-water bath for 30 min.^2^

**
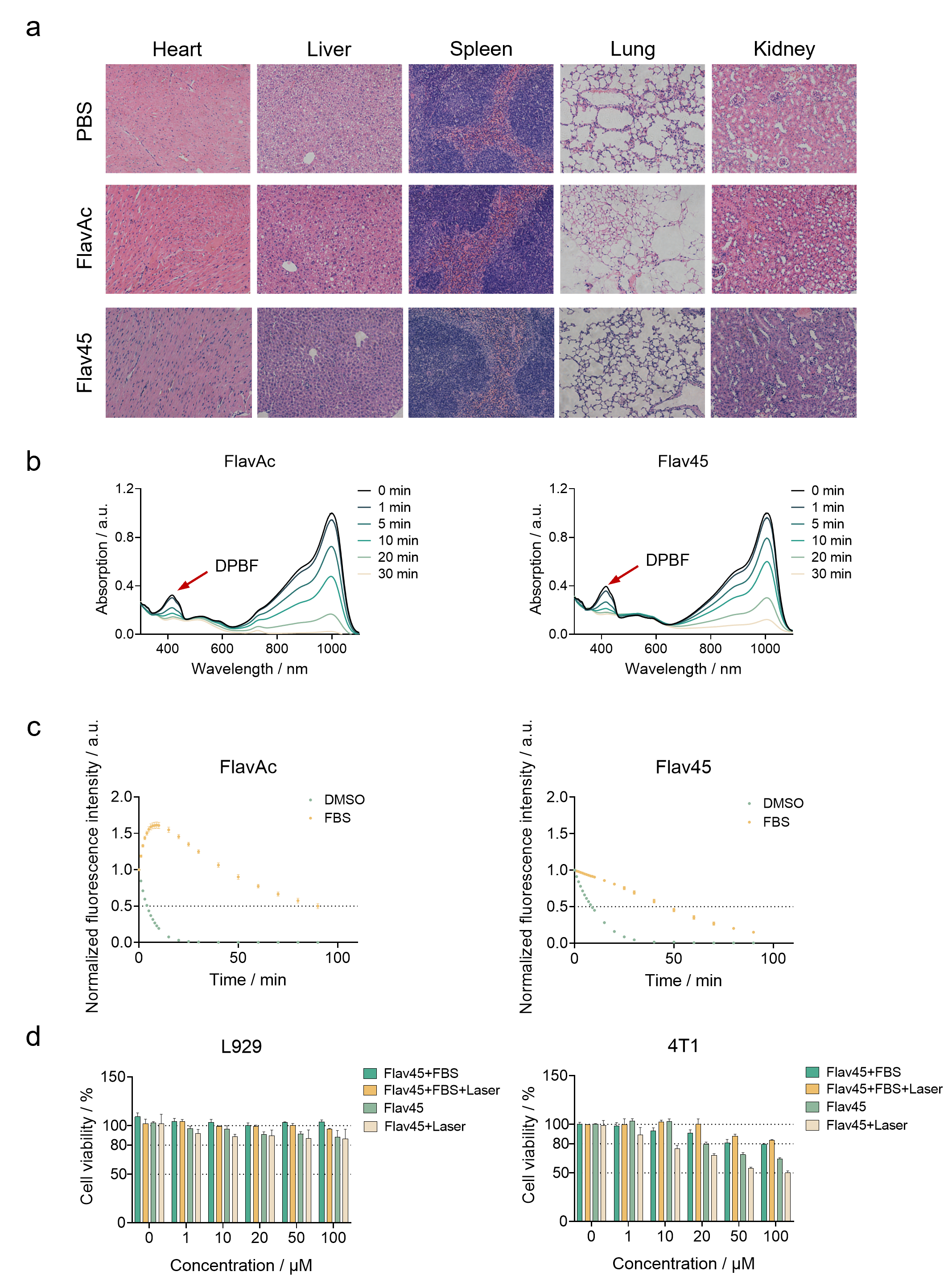
**

**Figure S17.** (a) H&E staining images of major mouse organs on day 2 after tail vein injection of PBS, FlavAc, and Flav45. (b) Time-dependent absorption spectra of FlavAc and Flav45 in DMSO with DPBF probe under a 980 nm laser (65 mW cm^-2^). (c) Photostability of FlavAc and Flav45 in DMSO and FBS (n=3). Data were normalized to the initial intensity. (d) Cytotoxicity of Flav45 at different concentrations on the mouse fibroblasts (L929) and breast cancer (4T1) cell lines (n=3).

**
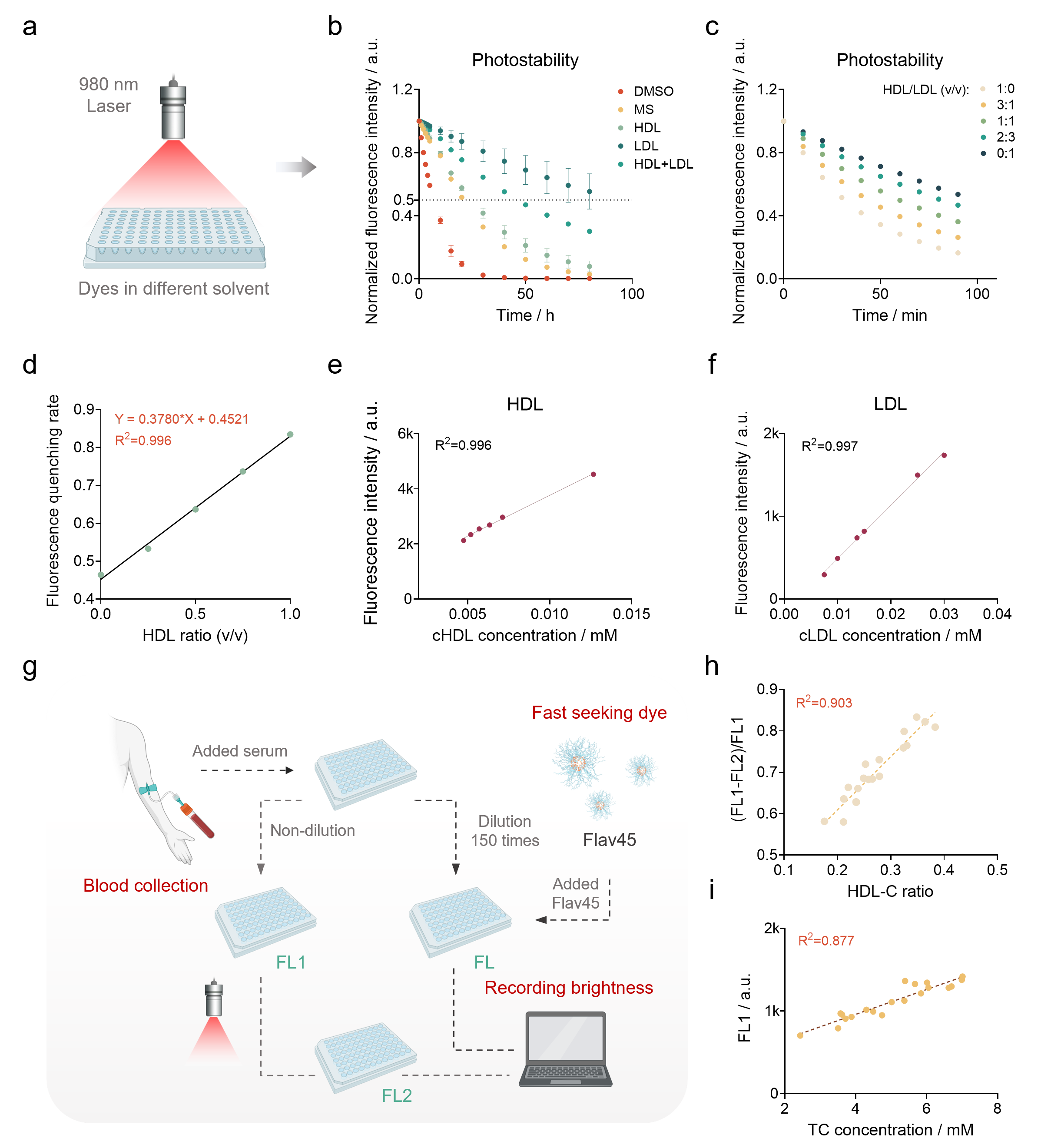
**

**Figure S18.** Blood lipids testing and distinguishing method. (a) Scheme for photostability test of Flav45 in different solvent. (b) Photostability of Flav45 in DMSO, MS, HDL, LDL, and the mixture of HDL and LDL (v/v=1/1). Data were normalized to the initial intensity. (c) Photostability of Flav45 in solvent containing different volume ratios of HDL and LDL. Data were normalized to the initial intensity. (d) Fluorescence quenching in solvent containing different volume ratios of HDL and LDL 90 min after 980 nm laser irradiation. (e) Linear relationship between fluorescence intensity of solvent with high-density lipoprotein cholesterol (HDL-C) concentration (diluted by PBS). (f) Linear relationship between fluorescence intensity of solvent with low-density lipoprotein cholesterol (LDL-C) concentration (diluted by PBS). (g) Schematic of blood lipids testing. (h) The correlation between fluorescence quenching of Flav45 mixed with serum and laser irradiated for 90 min and proportion of HDL-C to the sum of HDL-C and LDL-C in serum tested by the clinical assay. (i) The correlation between fluorescence intensity of Flav45 mixed with diluted serum and total cholesterol (TC) in serum tested by commercial kit. Concentration of Flav45 was 15 μM.

**
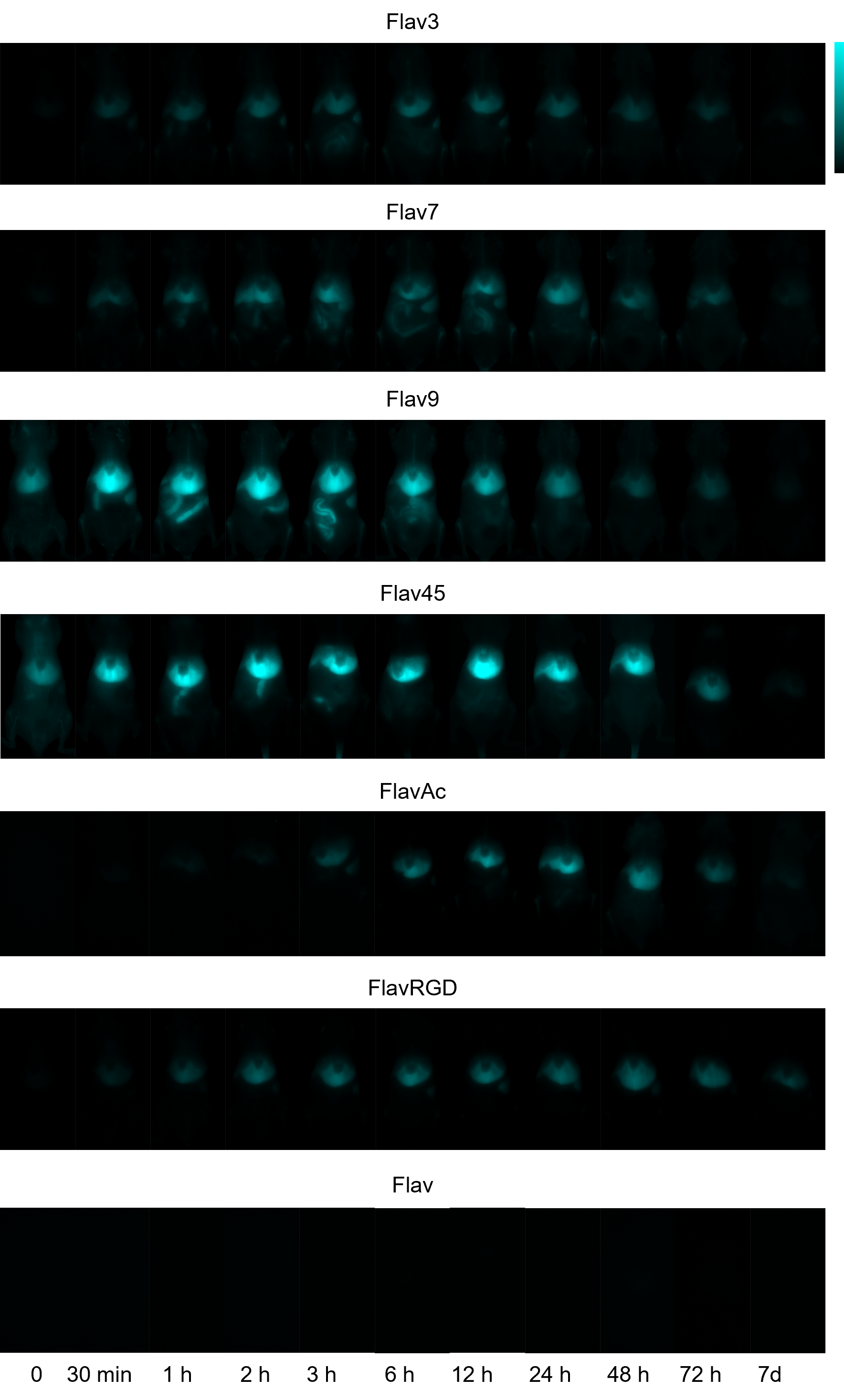
**

**Figure S19.** Whole-body NIR-II imaging of mice 0–168 h after tail vein injection of dyes. Injection dose: 0.2 μmol kg^-1^; over 1100 nm; exposure time: 10 ms; power density: 65 mW cm^-2^.


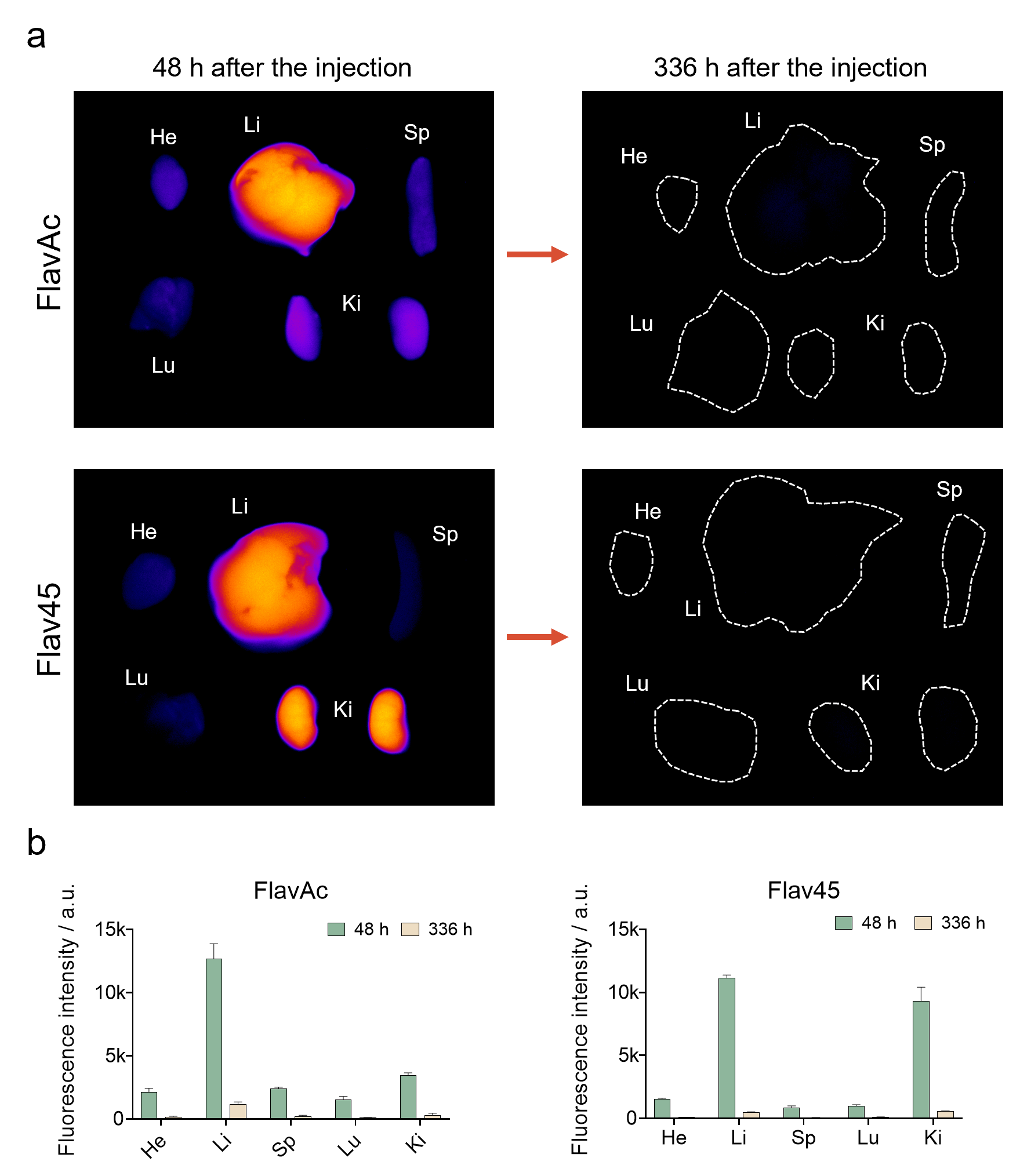


**Figure S20.** (a) NIR-II imaging of mouse main organs obtained 48 h and 336 h after intravenous administration of FlavAc and Flav45. (b) NIR-II fluorescence statistics of organs in a (n=3). He: heart; Li: liver; Sp: spleen; Lu: lung; Ki: kidney.


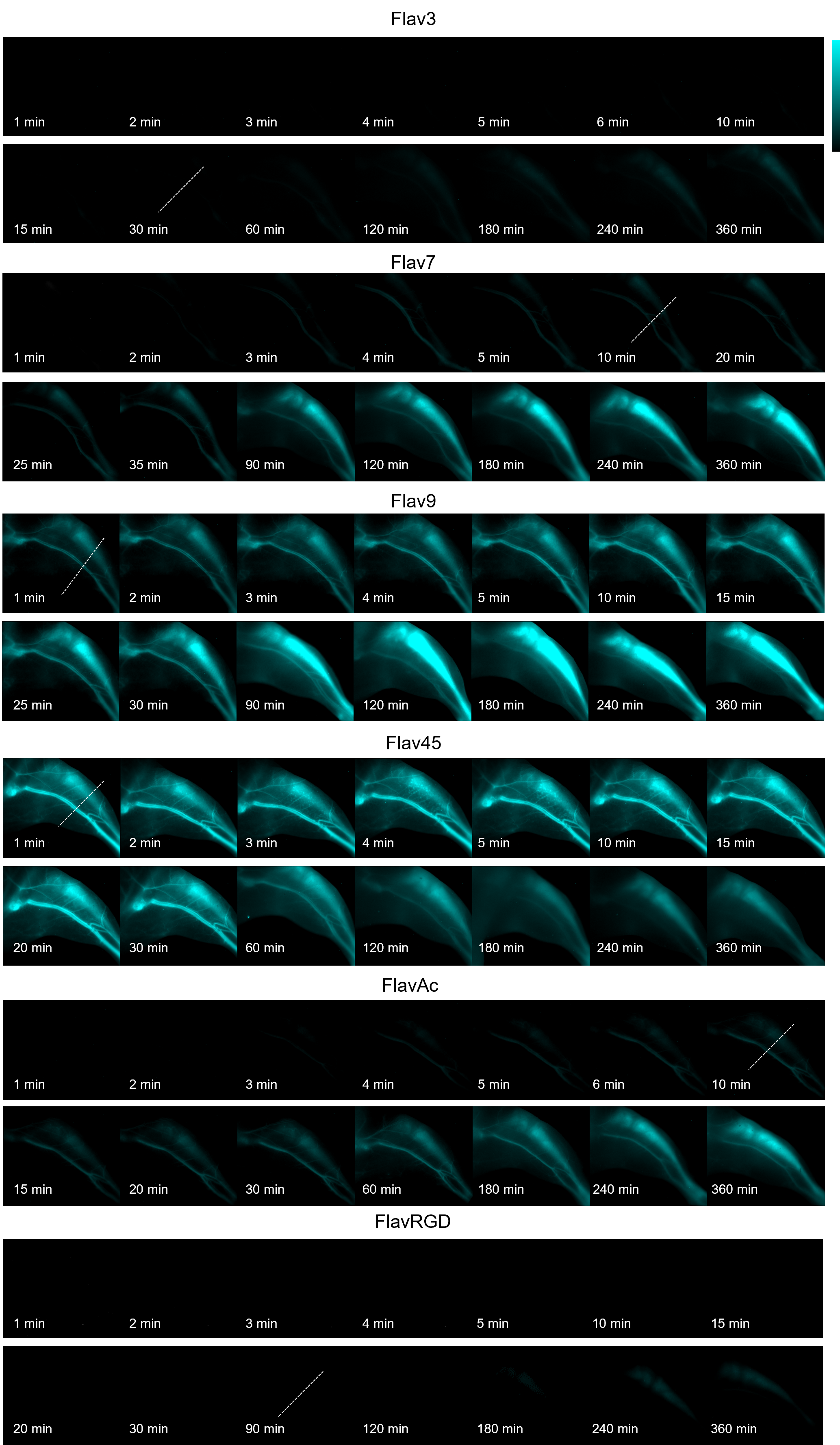


**Figure S21.** NIR-II hindlimb vessel imaging of mice 0–6 h after intravenous injection of dyes. Injection dose: 600 μM, 200 μL; over 1200 nm; exposure time: 40 ms; power density: 65 mW cm^-2^.

**
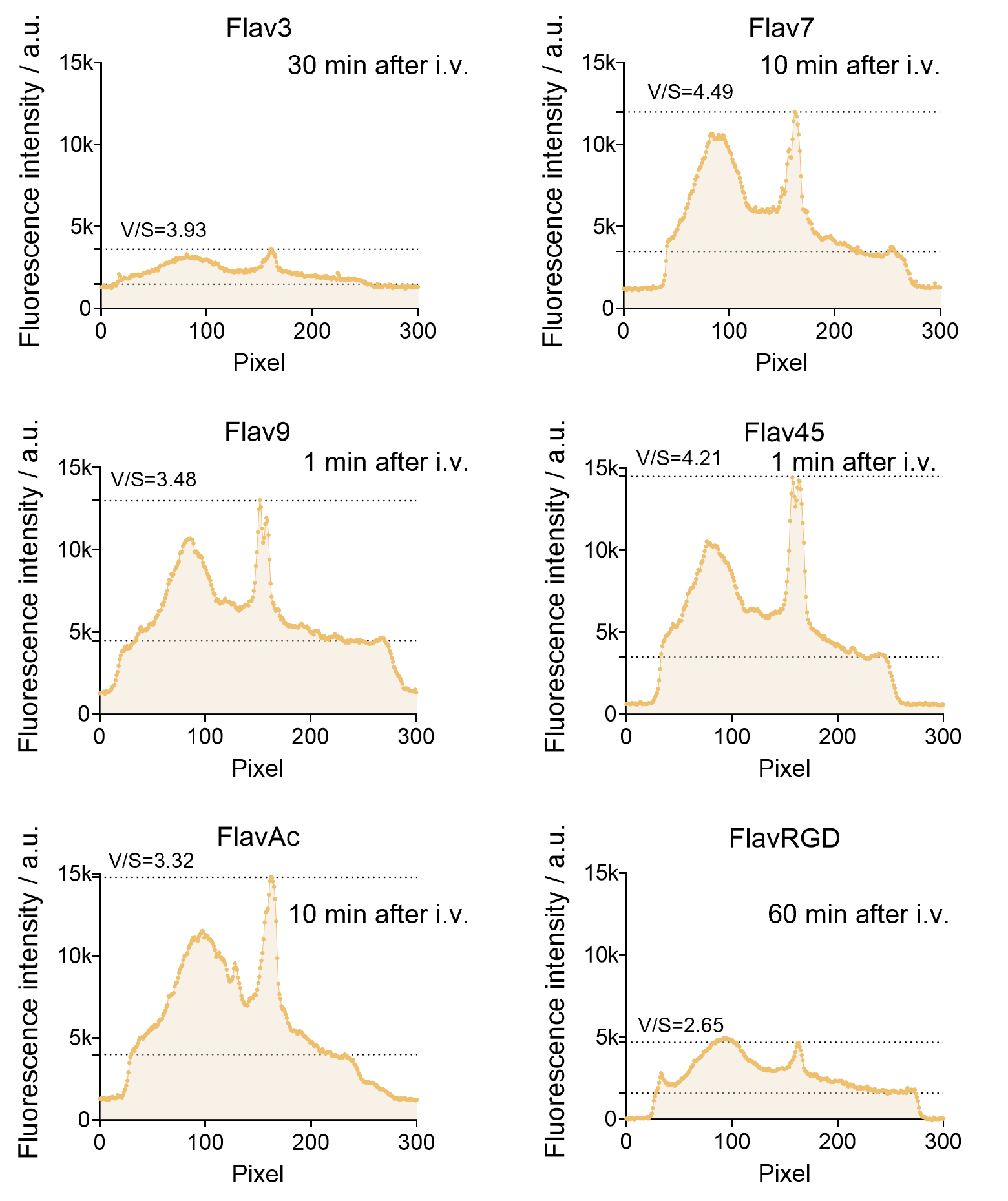
**

**Figure S22.** Fluorescence intensity and statistical analysis of hindlimb blood vessels​​ along the white dashed lines in figure S21, showing the vessel-to-skin (V/S) ratio analyzed at the time point of optimal contrast​​.

**
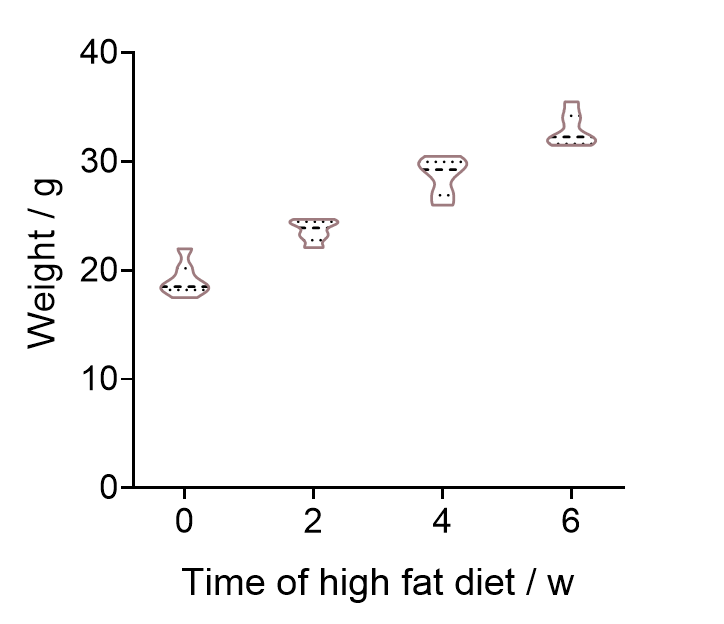
**

**Figure S23.** The weight changes of mice by high diet fat (n=6).

**
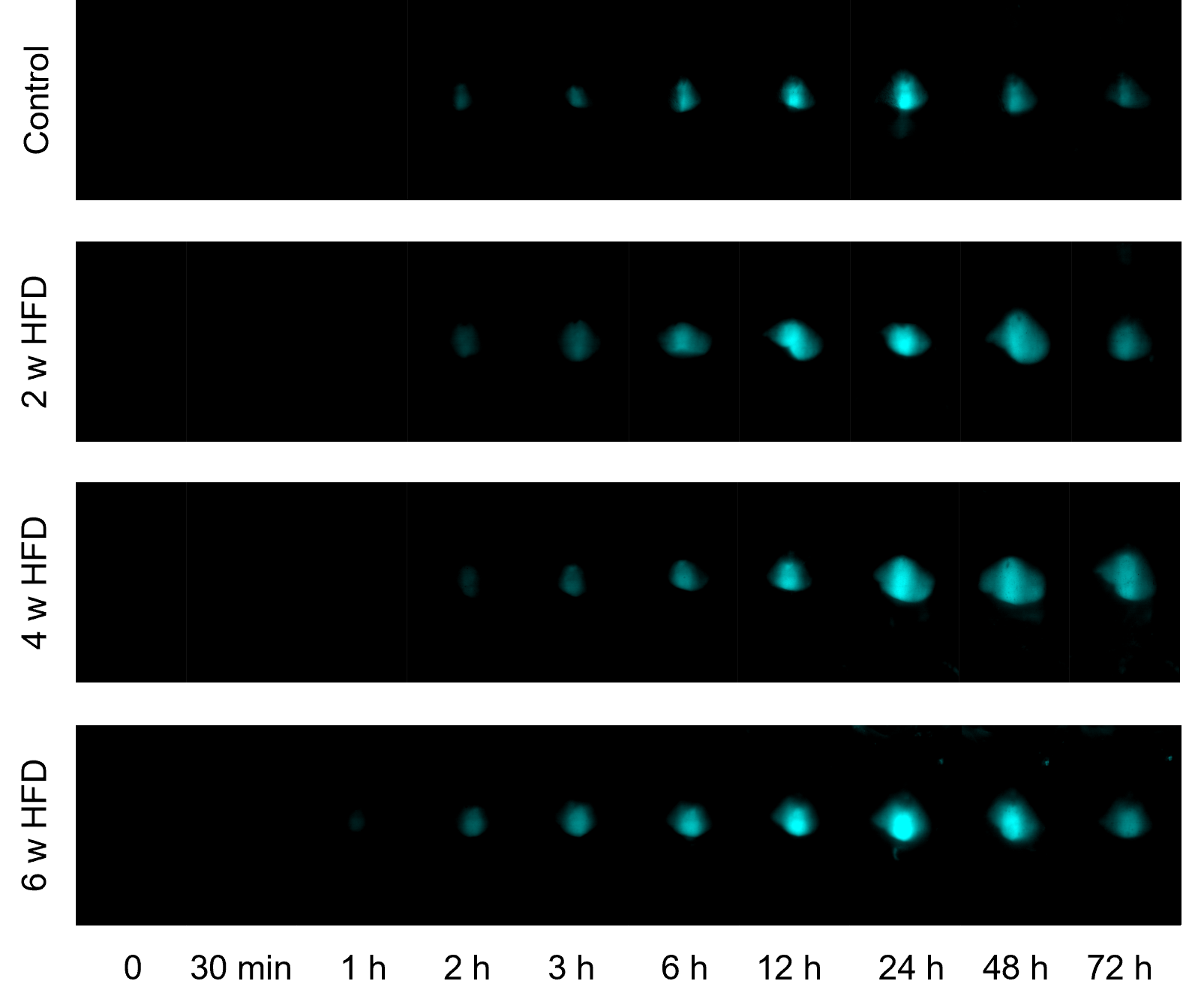
**

**Figure S24.** NIR-II imaging of living mice in the control group and the HFD group after tail vein injection of FlavAc over time. Injection dose: 0.6 μmol kg^-1^; exposure time: 10 ms; over 1100 nm; power density: 65 mW cm^-2^.


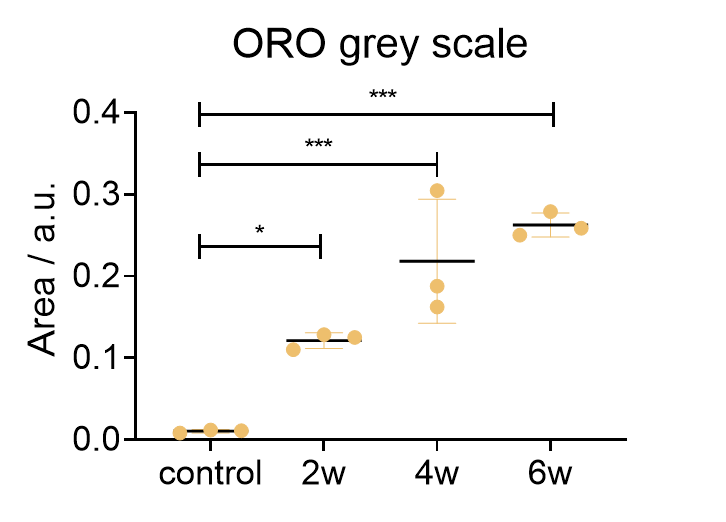


**Figure S25.** Gray-scale quantification of Oil Red O staining in mouse liver sections after different durations of high-fat feeding.

**Note:** Images were captured using an inverted fluorescence microscope, converted to 32-bit format in ImageJ software, and analyzed for areas within the threshold range of 15 to 110.

**
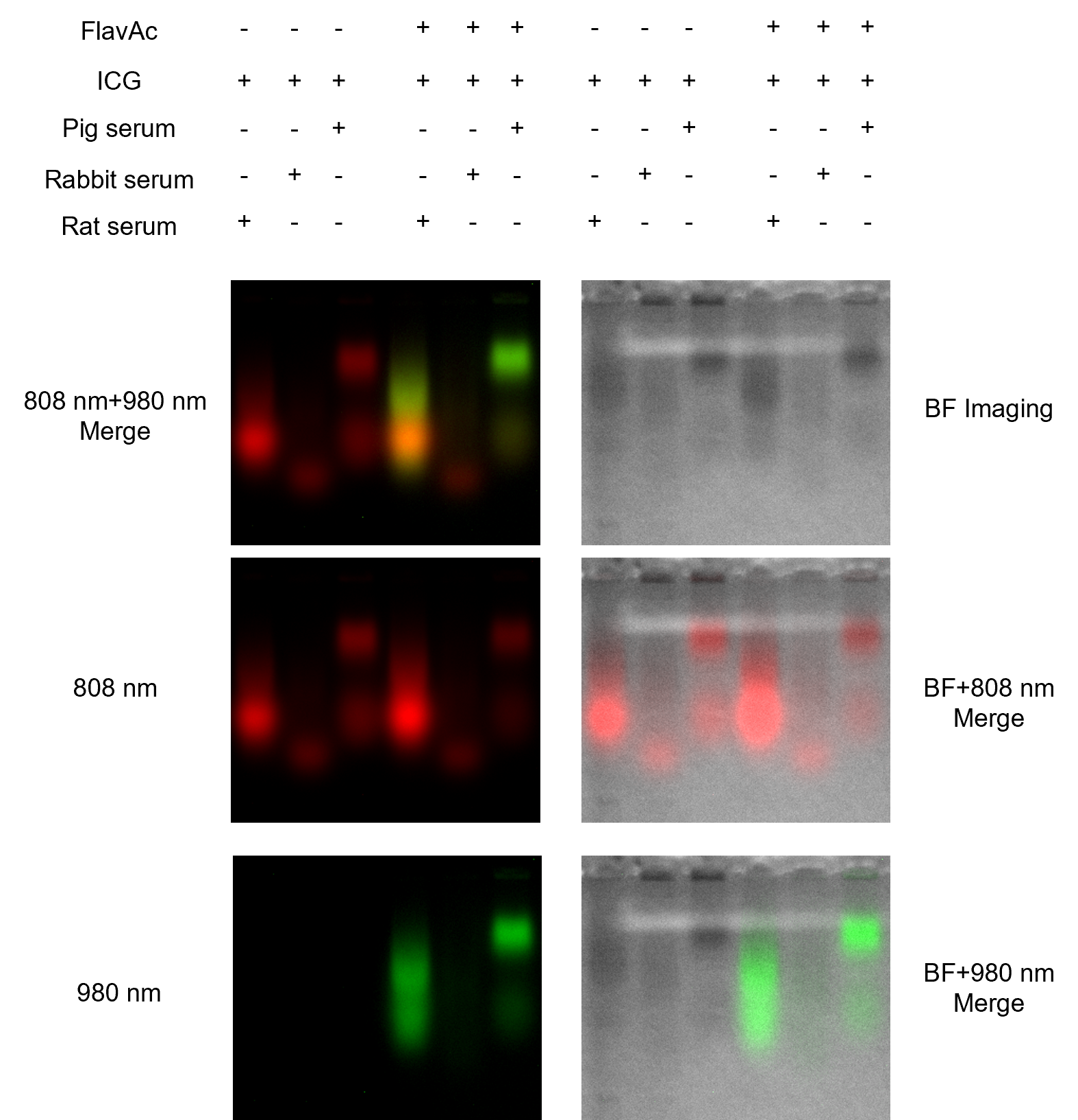
**

**Figure S26.** Separation of serum co-labeled by albumin-seeking dyes (ICG) and lipoprotein-seeking dyes (FlavAc) by agarose gel electrophoresis. BF: bright field.

**
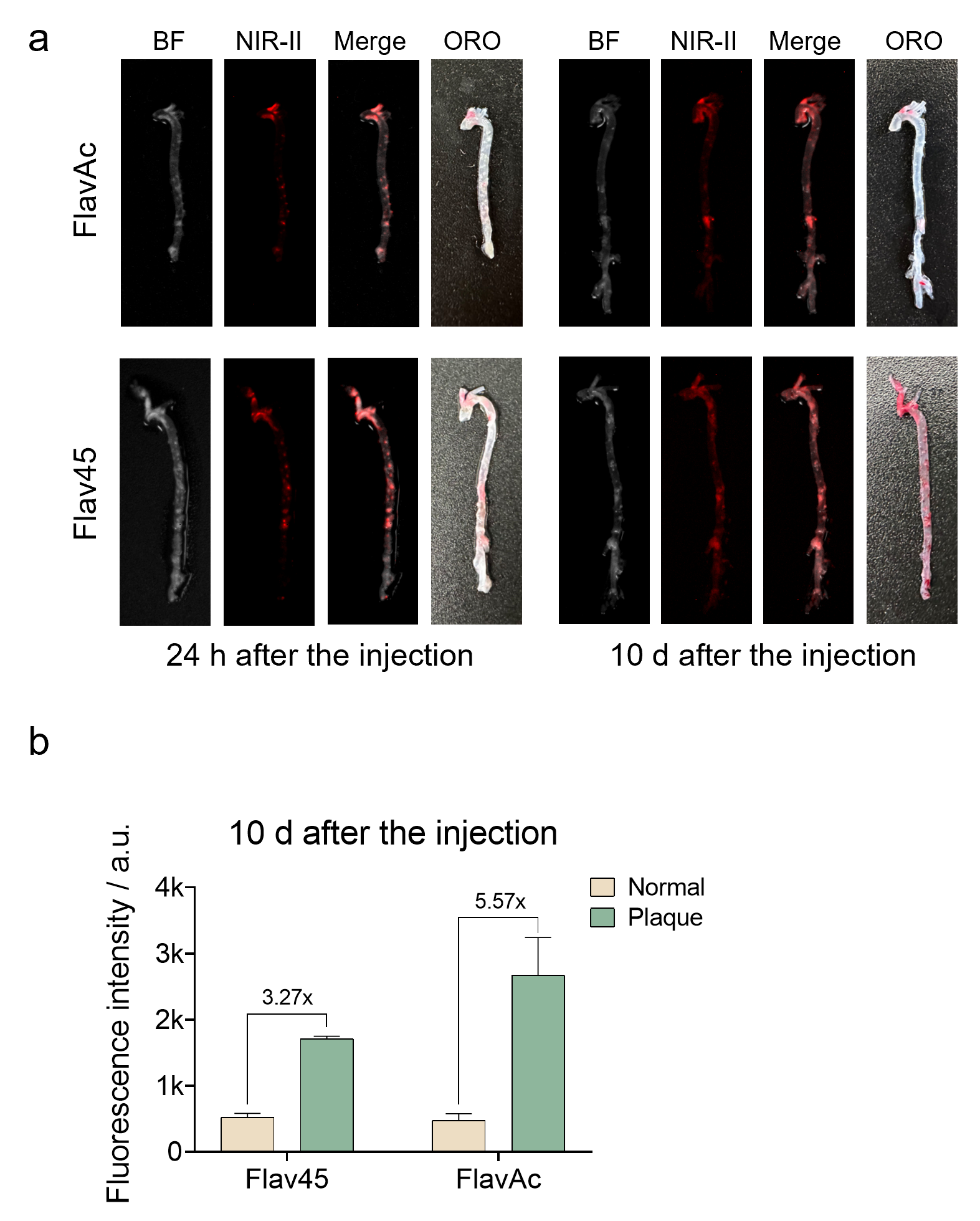
**

**Figure S27.** (a) Ex vivo imaging of aortas in ApoE^-/-^ mice (12 w high fat diet feeding) after intravenous injection of FlavAc and Flav45 for 24 h and 10 d. (b) NIR-II fluorescence intensity of plaque and normal vascular tissue in ApoE^-/-^ mice 10 d post-administration from a.

**
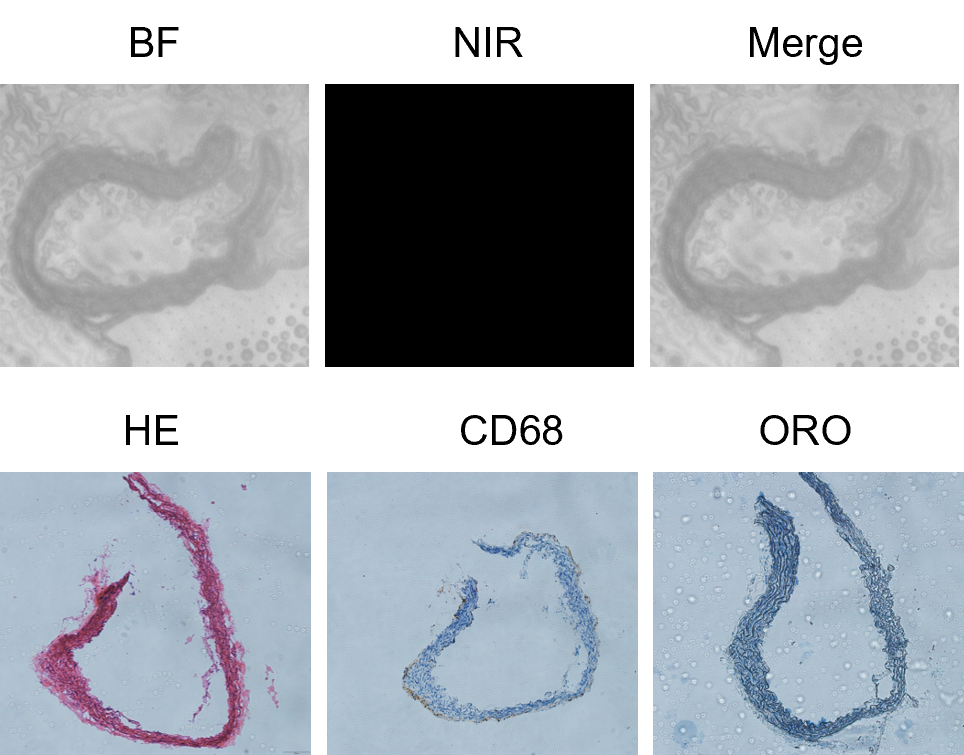
**

**Figure S28.** Comparison of NIR-II imaging, H&E staining, CD86 staining, and ORO staining for normal aorta.

**NMR spectra, chromatogram and mass spectra**

^1^H NMR spectra of IR-6B3Ac


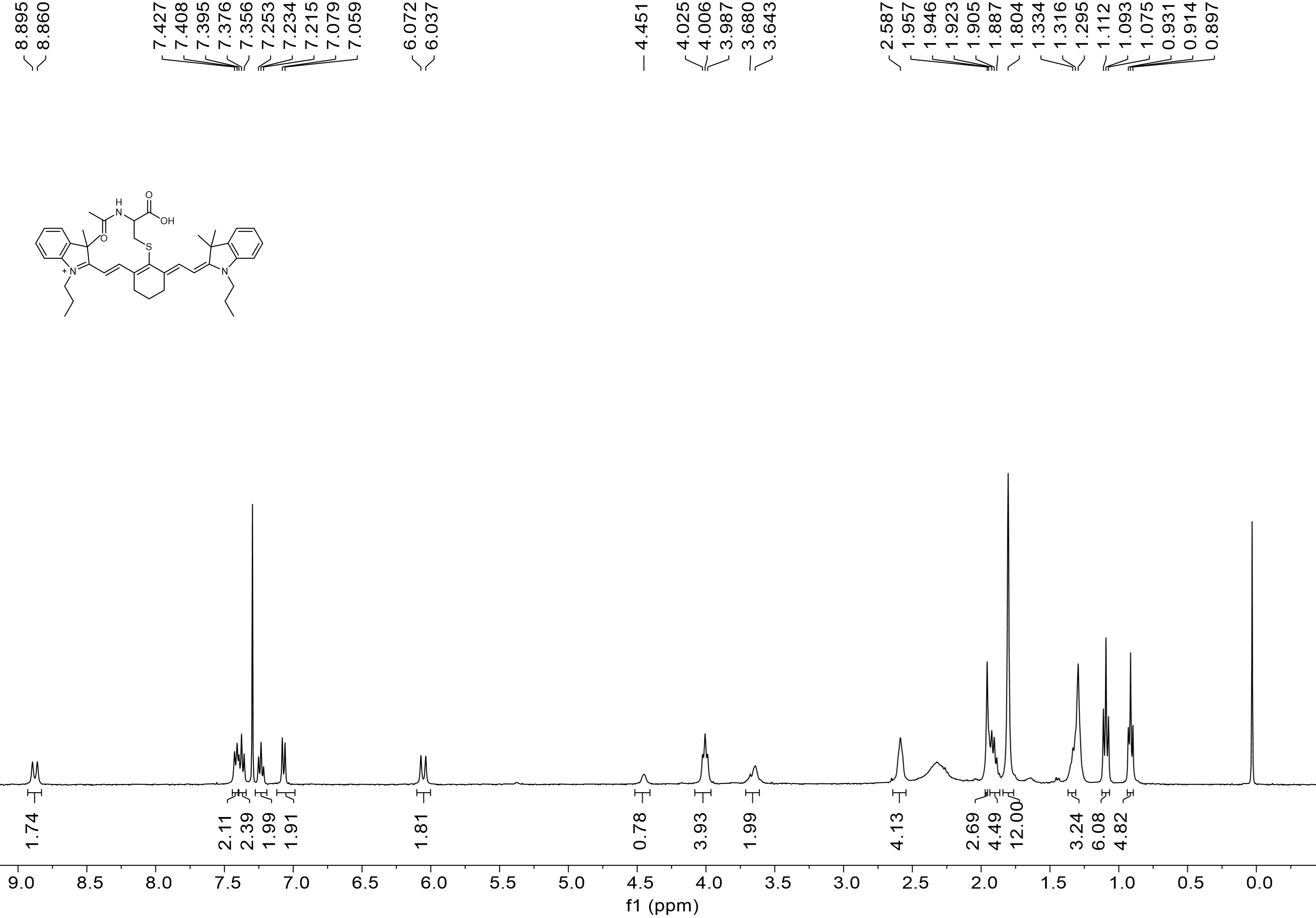


Chromatogram of IR-6B3Ac


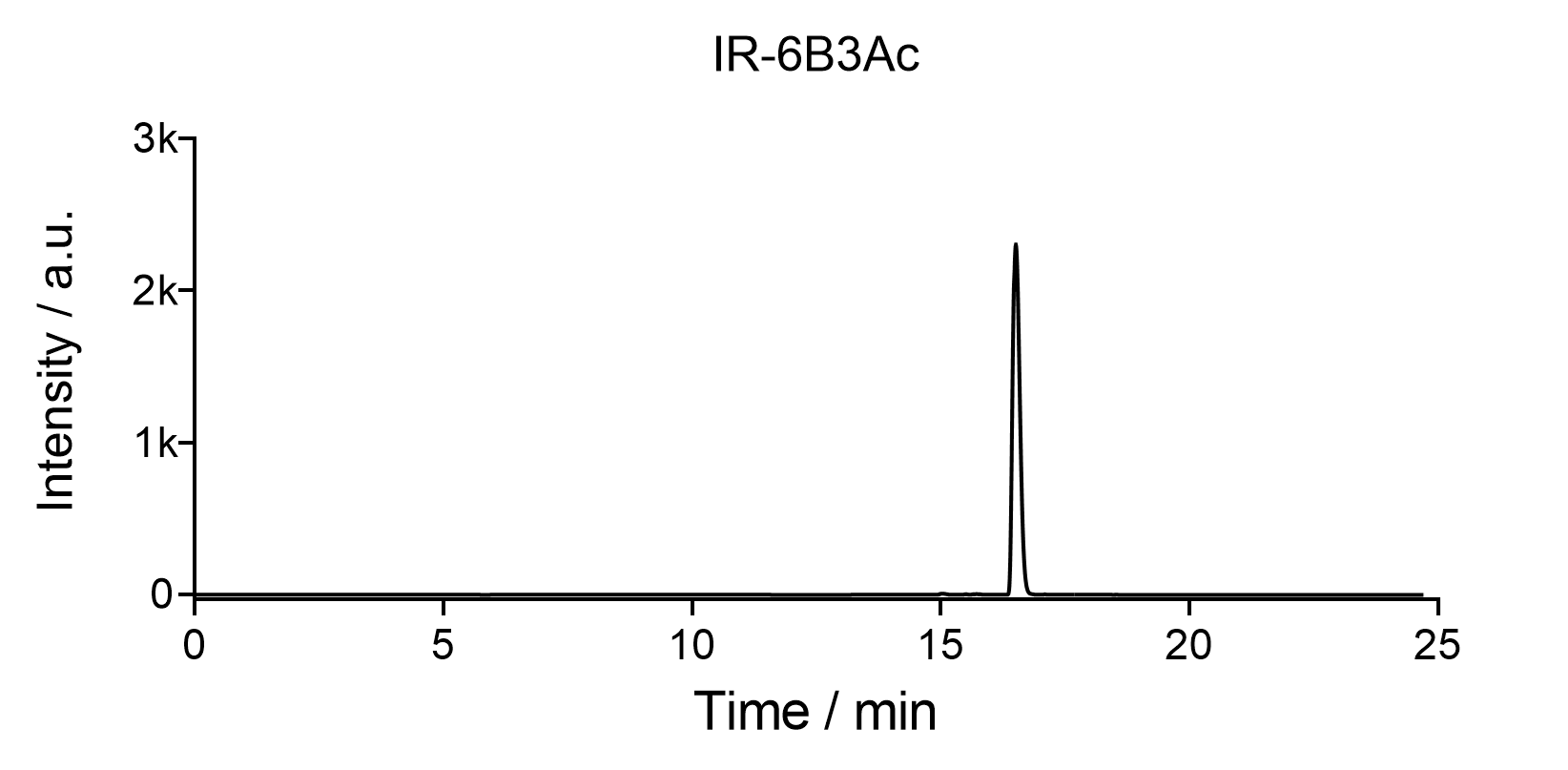


LC-HRMS spectra of IR-6B3Ac


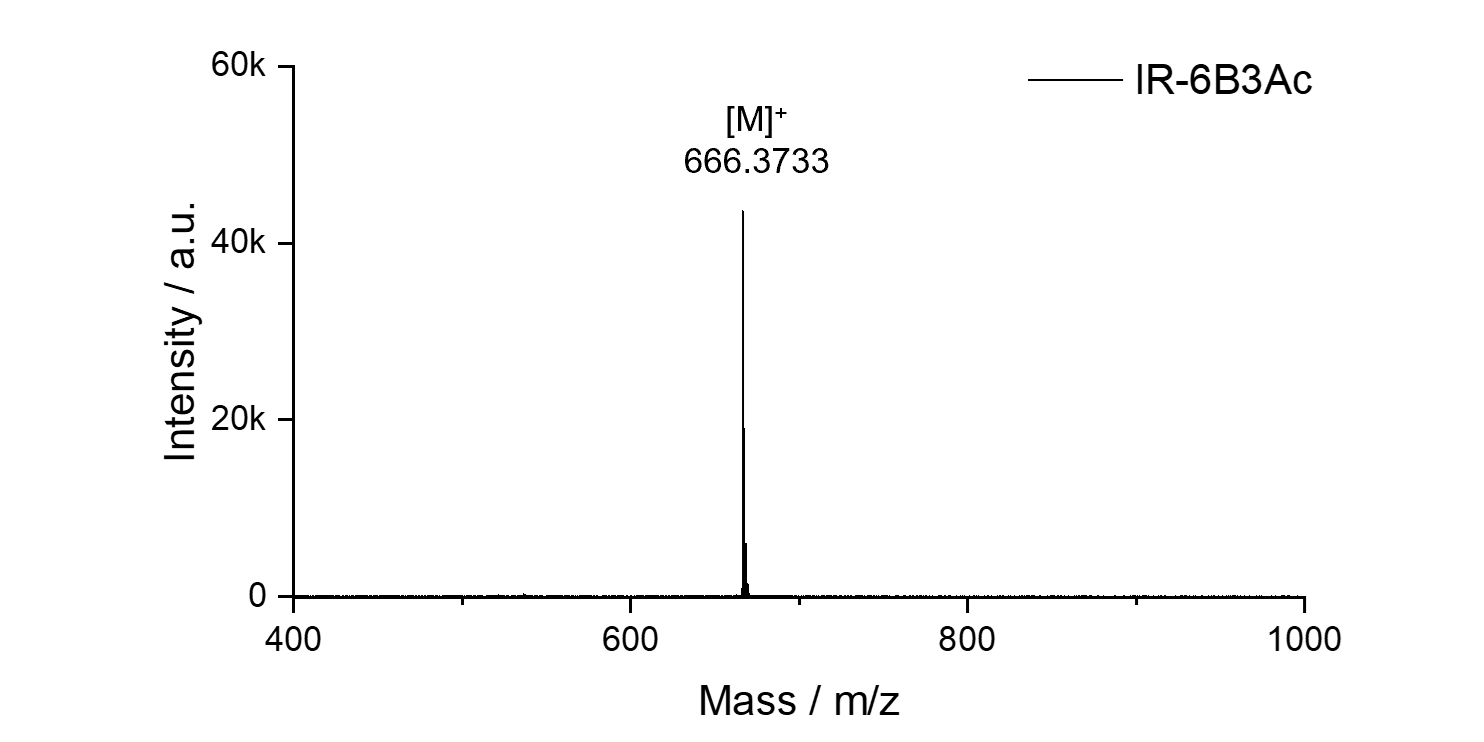


^1^H NMR spectra of IR-6B6CAc


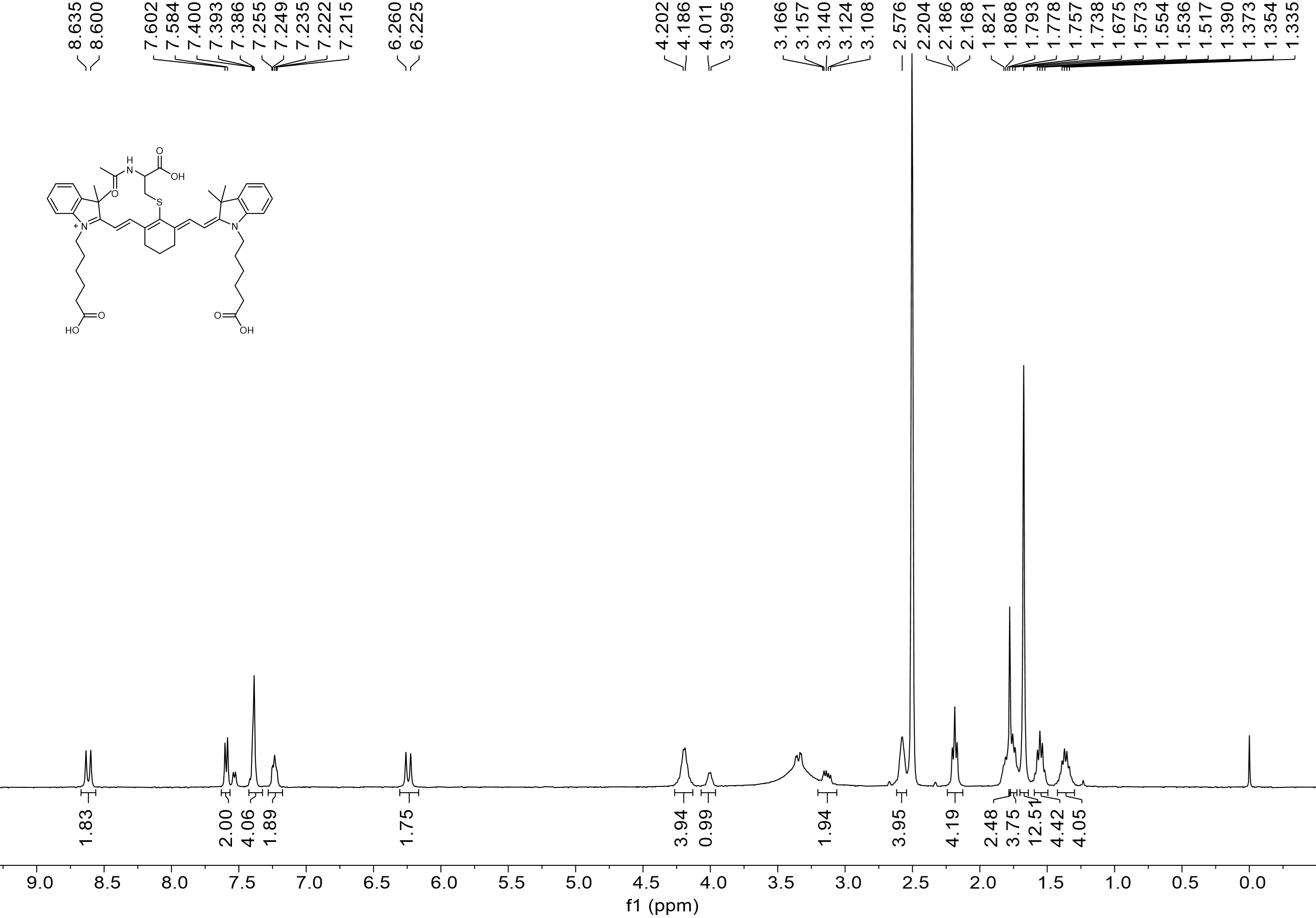


Chromatogram of IR-6B6CAc


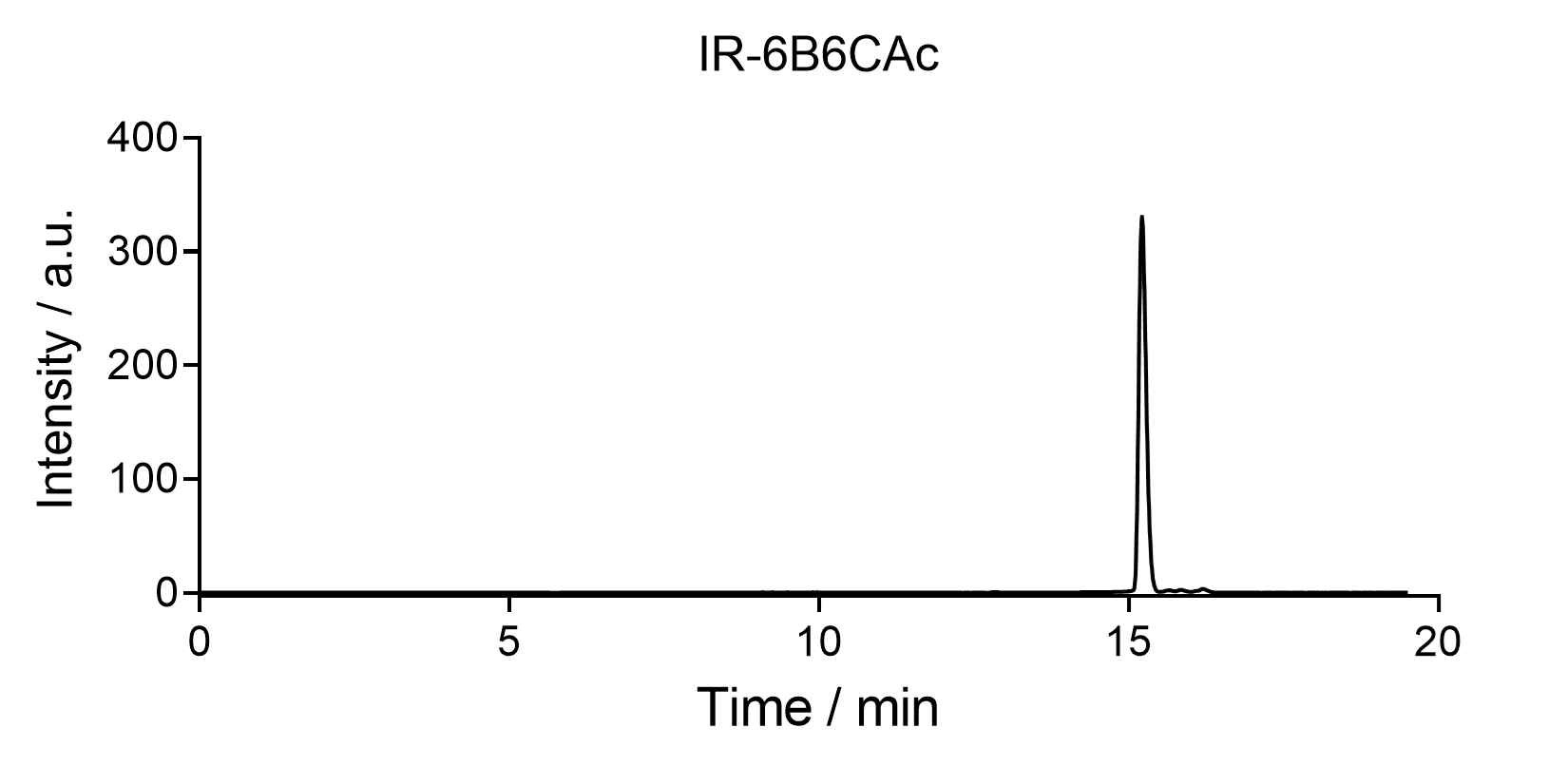


LC-HRMS spectra of IR-6B6CAc


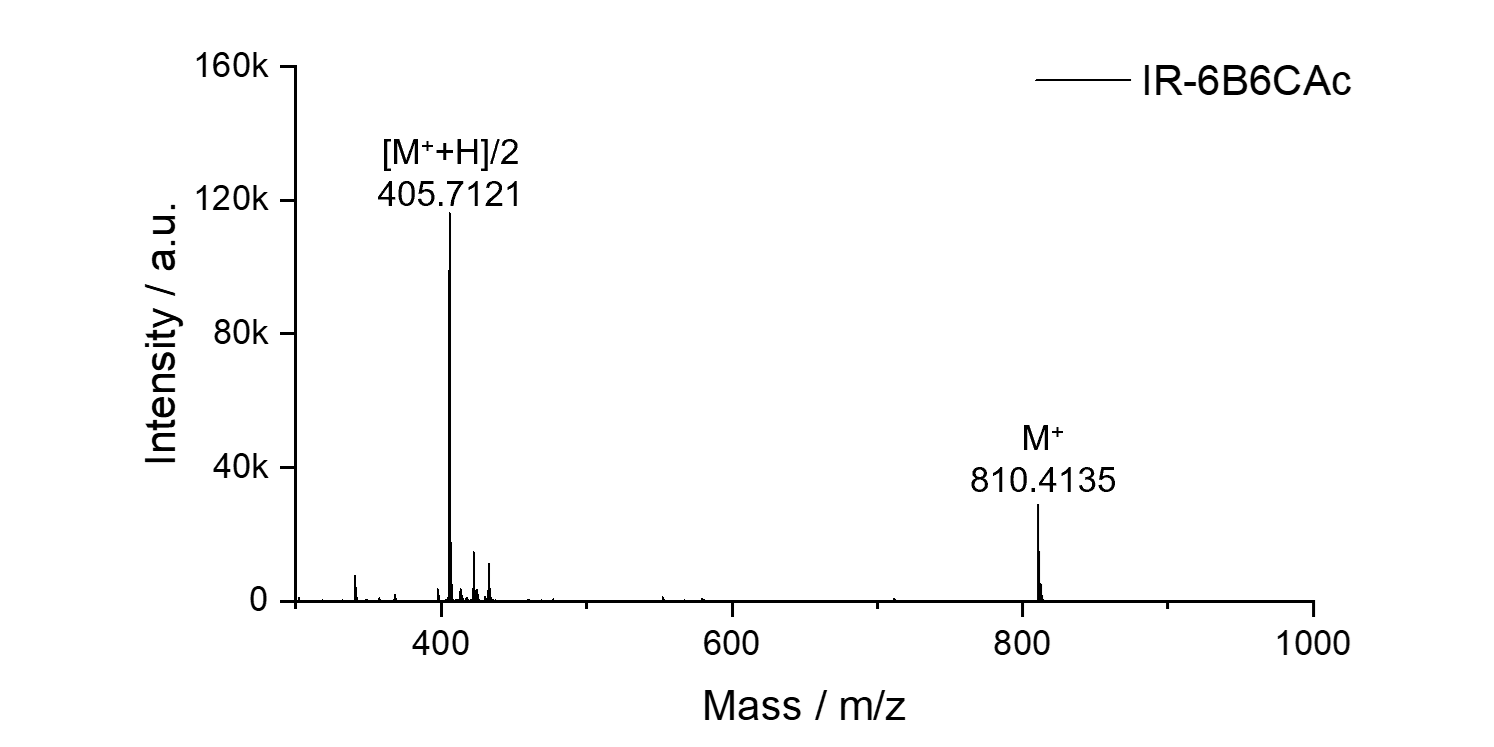


^1^H NMR spectra of IR-6N3Ac


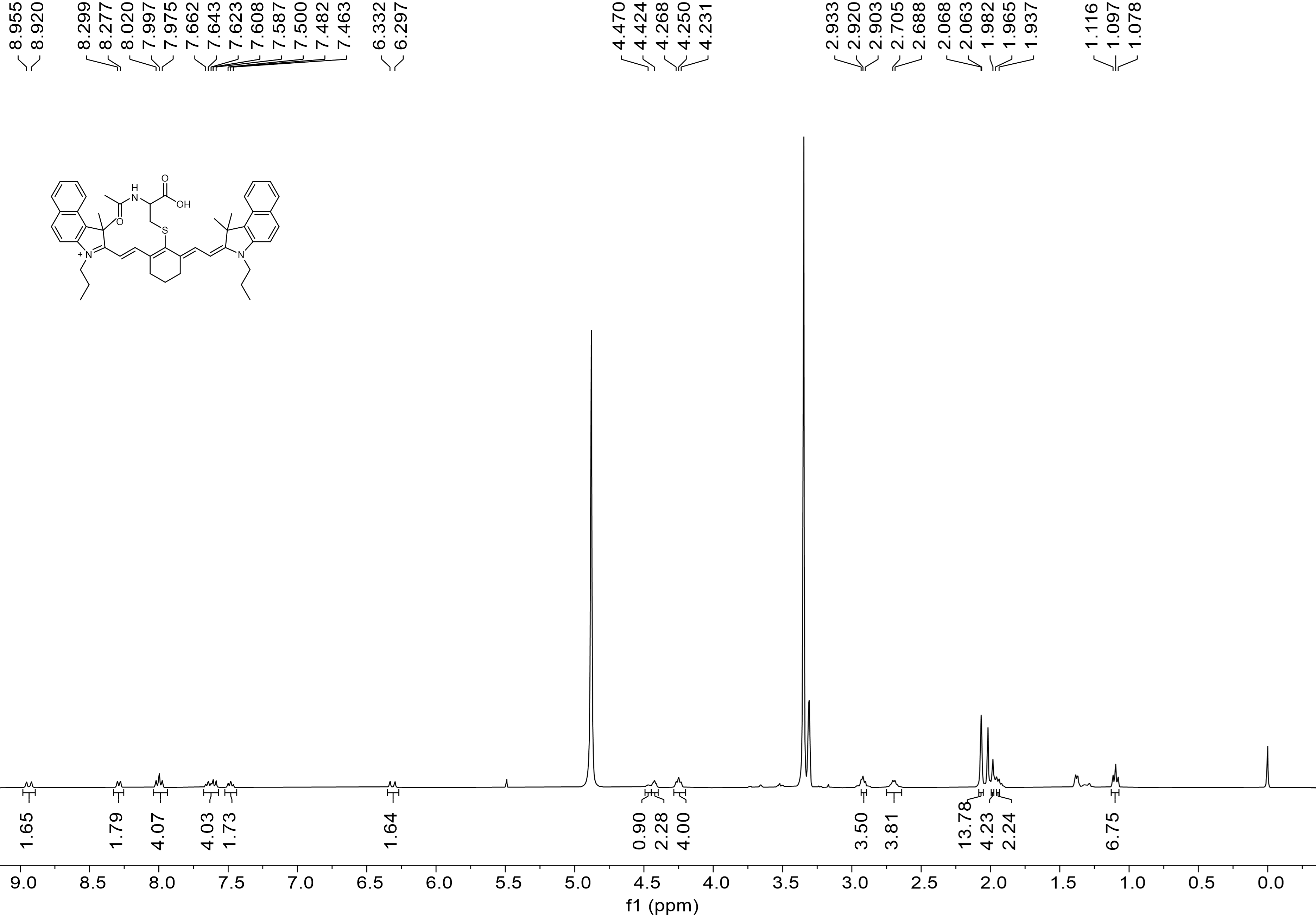


Chromatogram of IR-6N3Ac


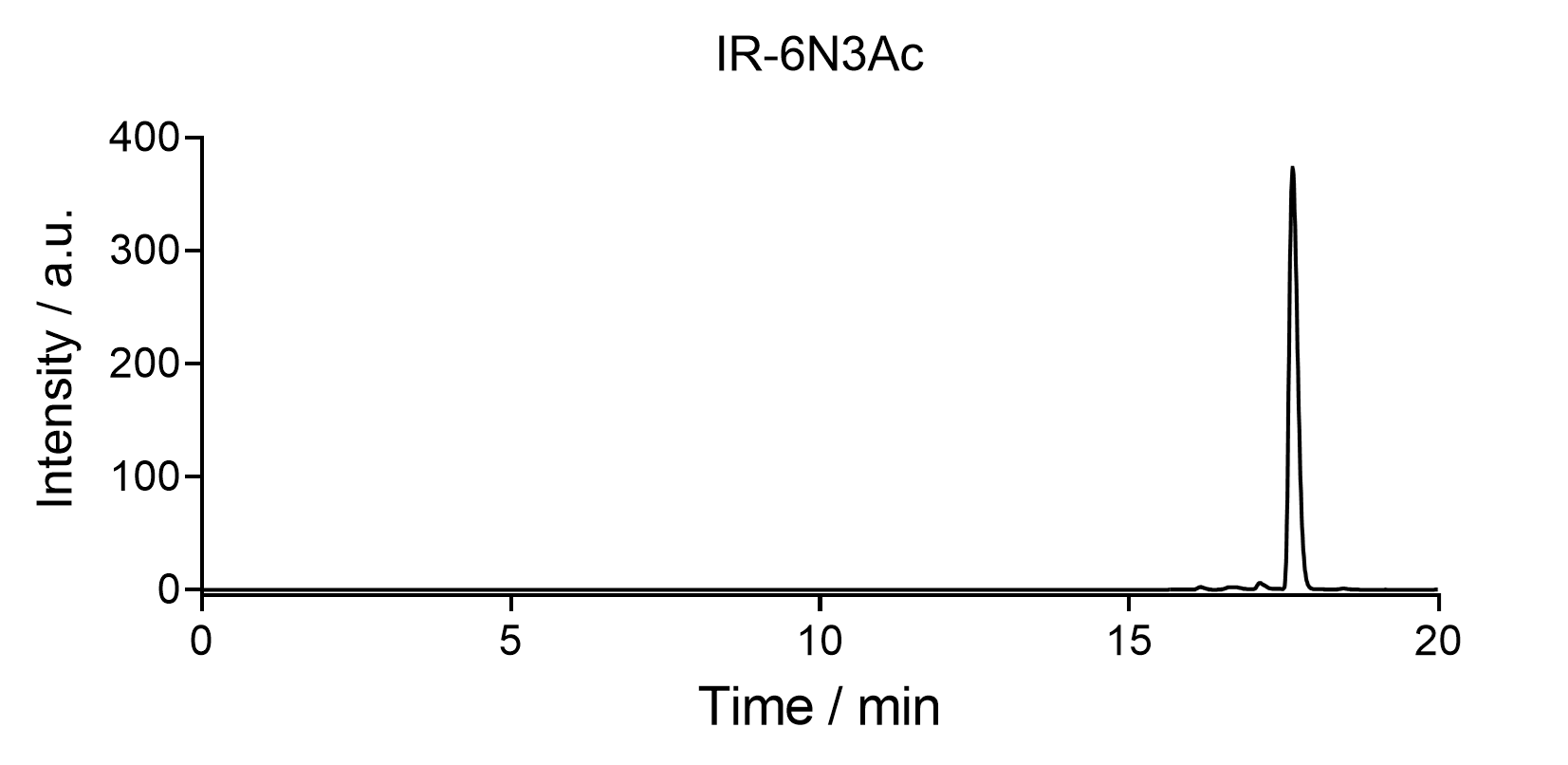


LC-HRMS spectra of IR-6N3Ac


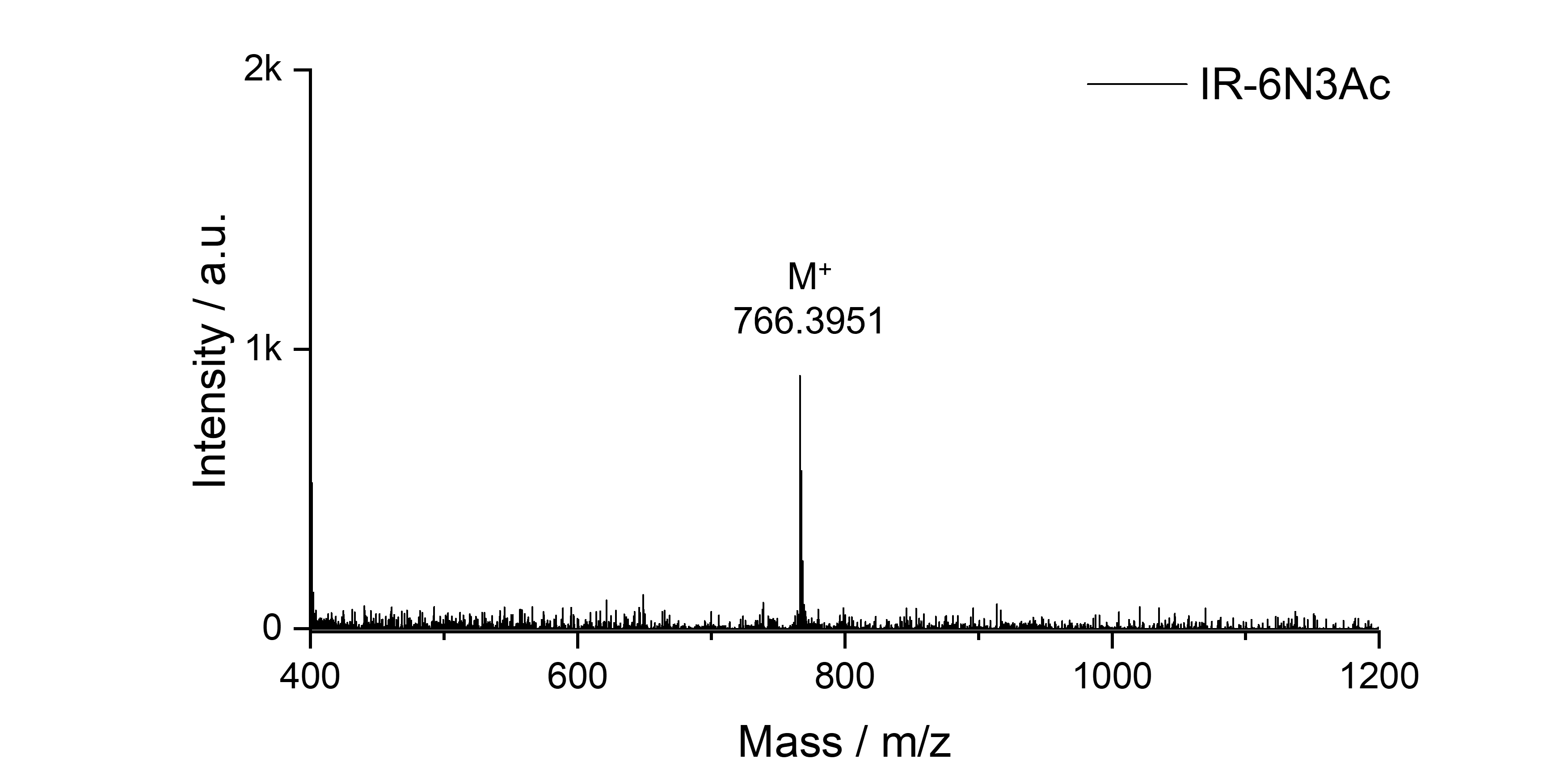


^1^H NMR spectra of IR-6N4SAc


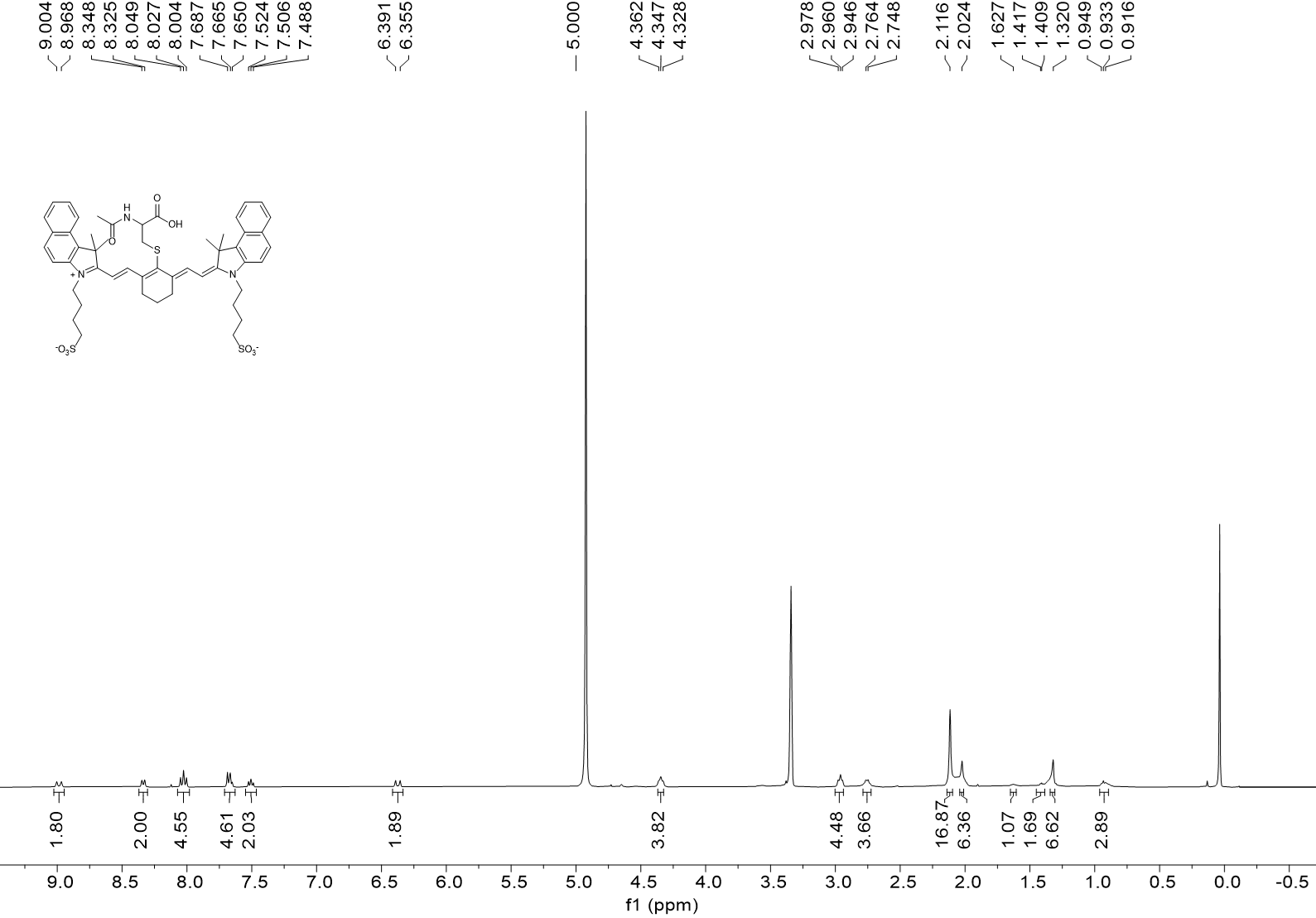


Chromatogram of IR-6N4SAc


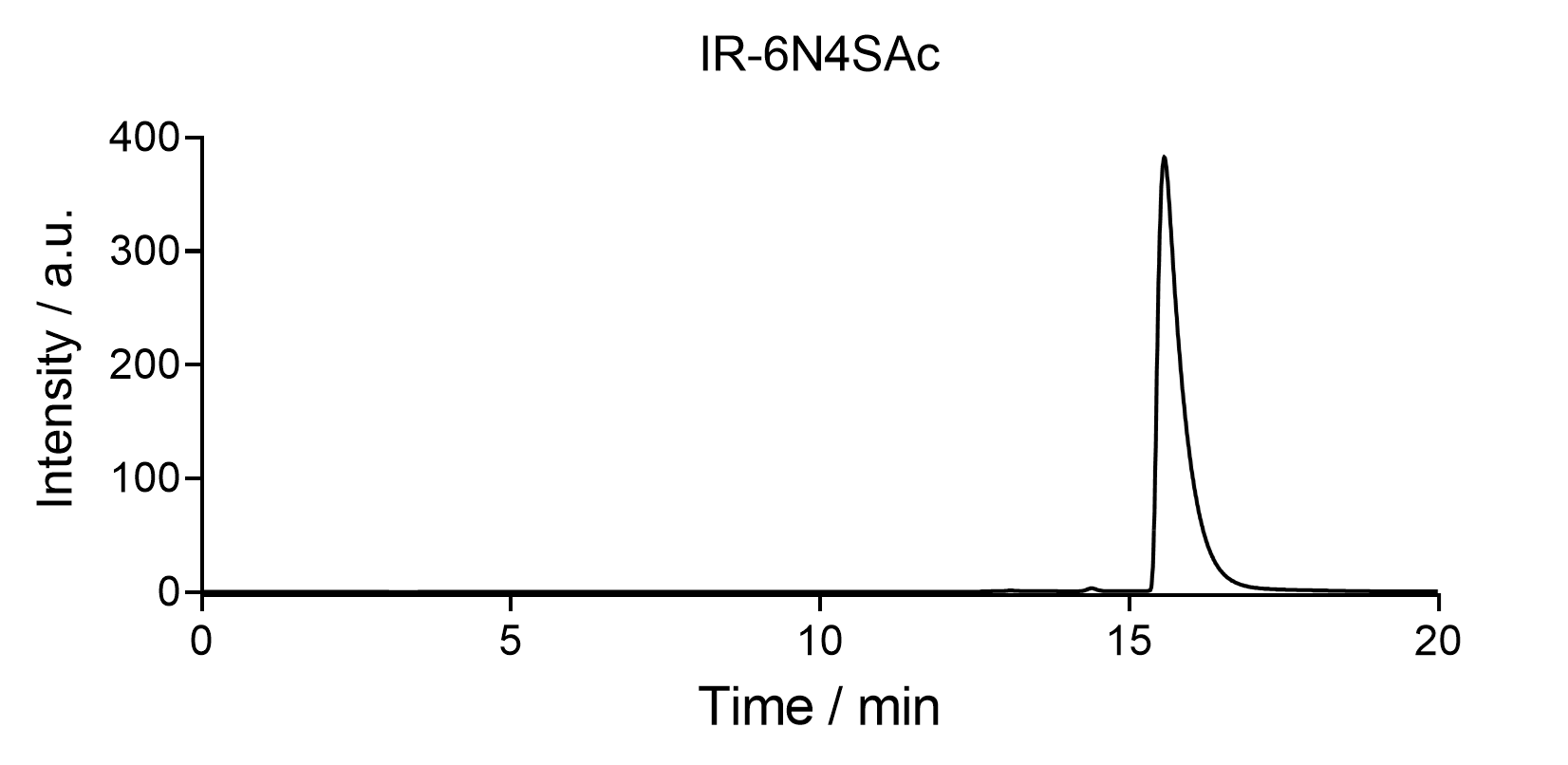


LC-HRMS spectra of IR-6N4SAc

.
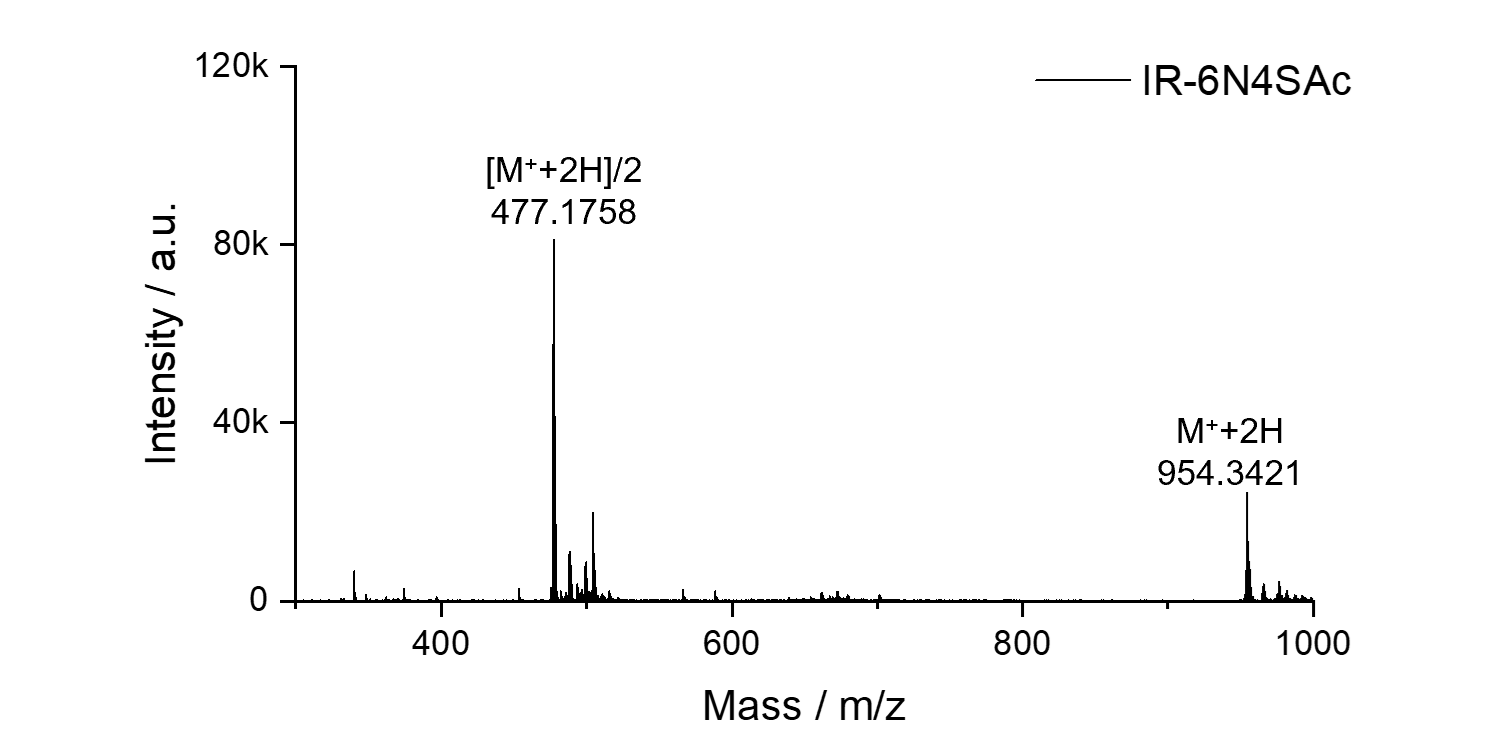


^1^H NMR spectra of IR-6B9Ac.


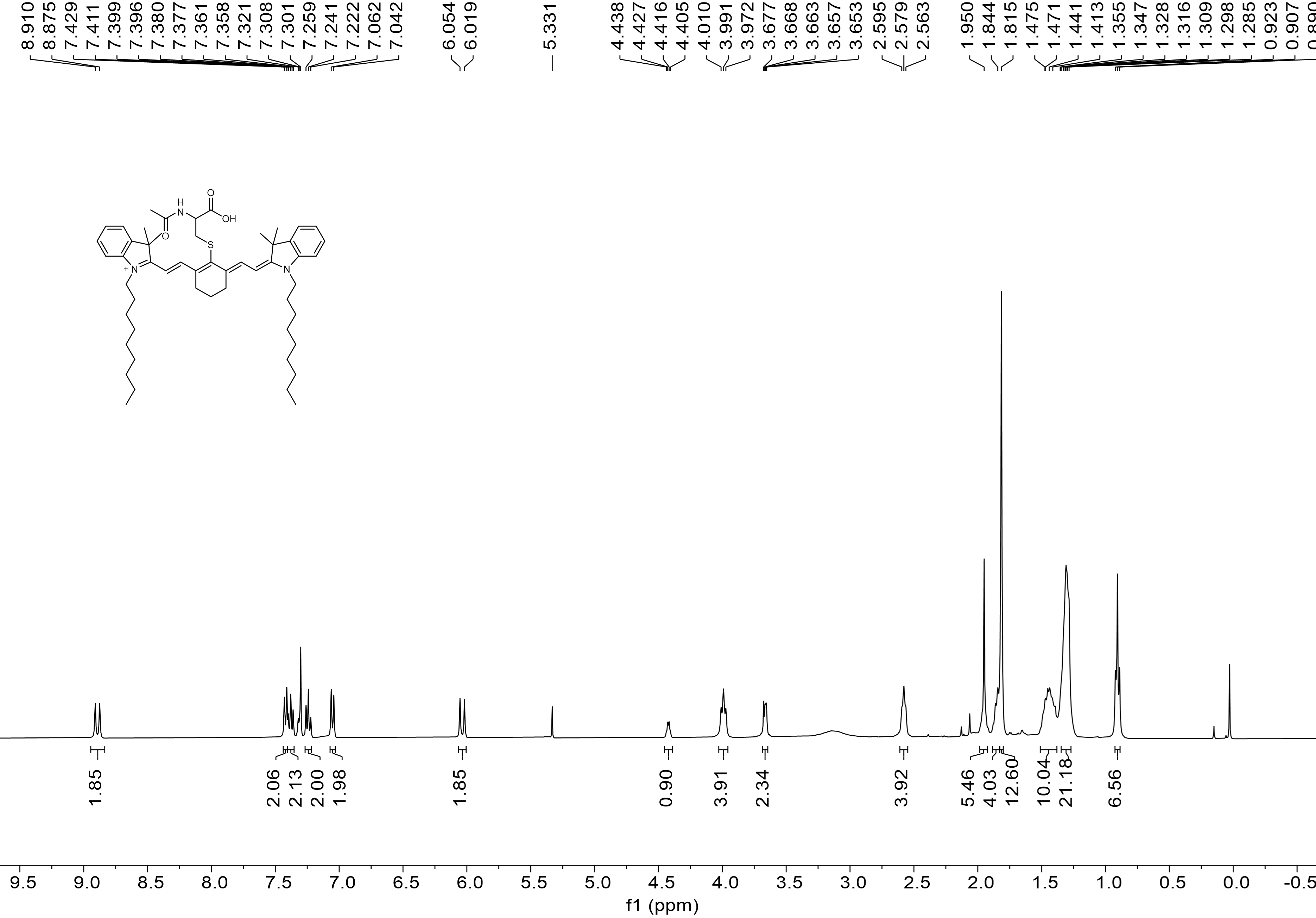


Chromatogram of IR-6B9Ac


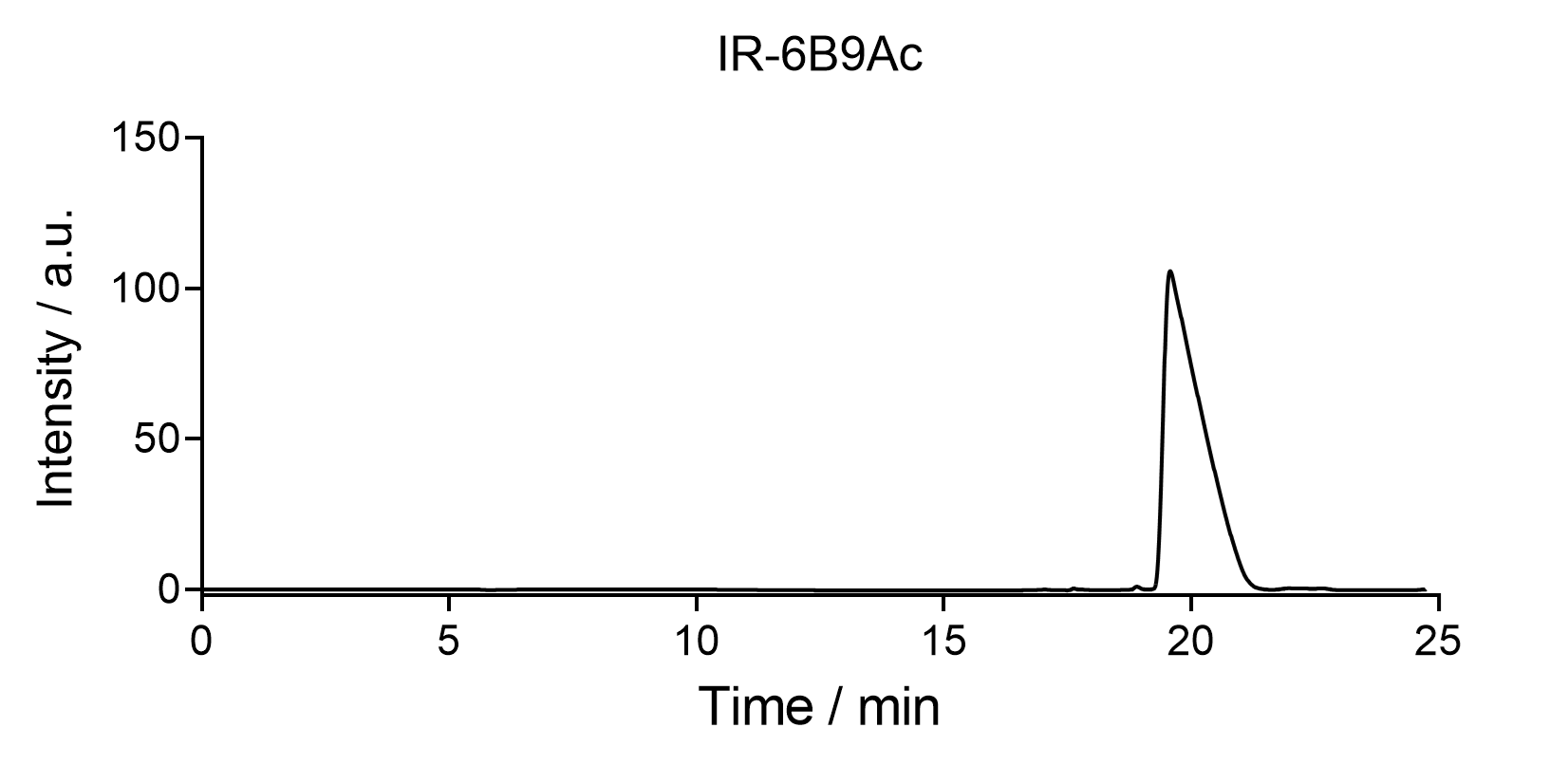


LC-HRMS spectra of IR-6B9Ac


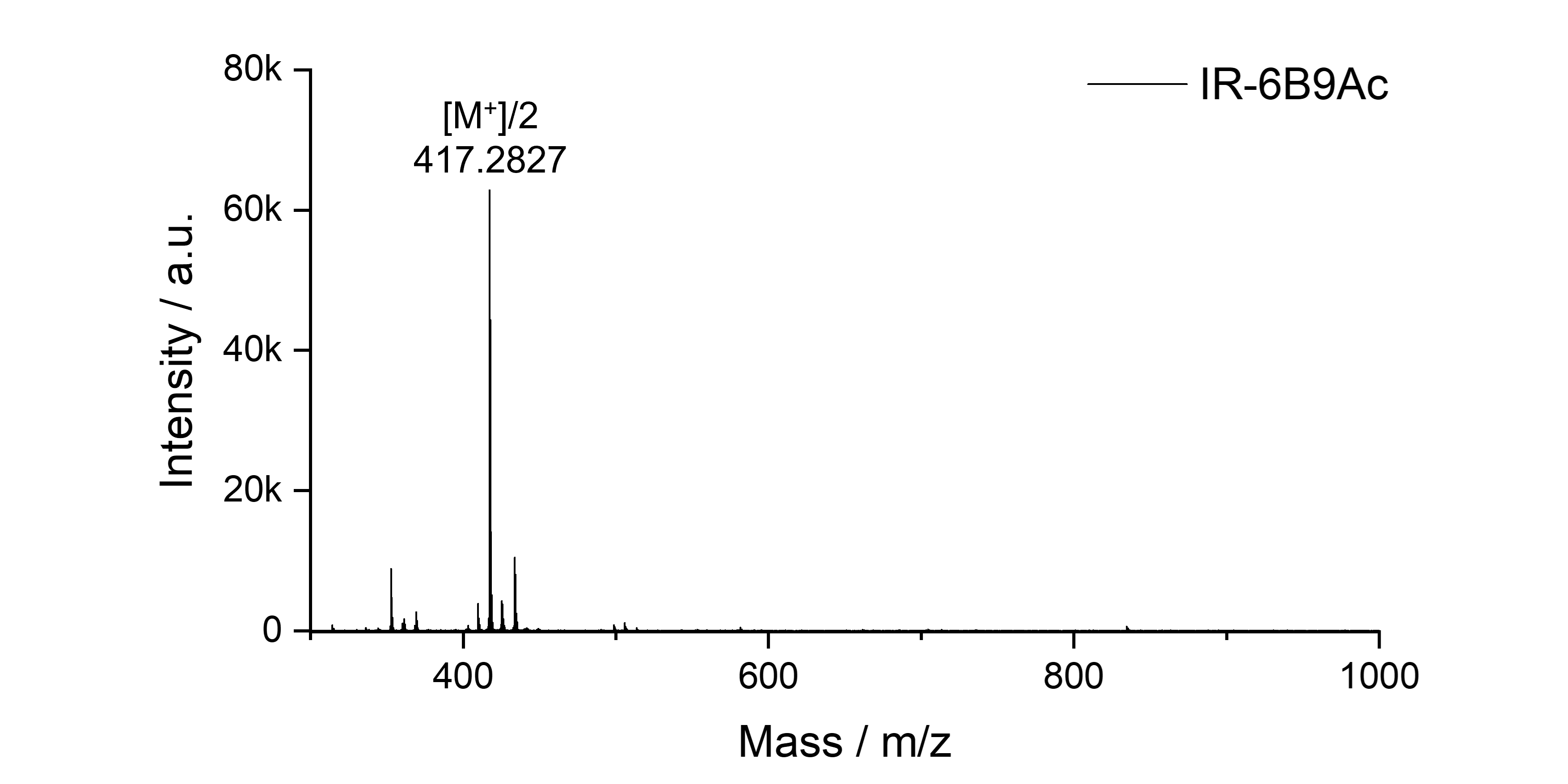


^1^H NMR spectra of IR-6B12Ac


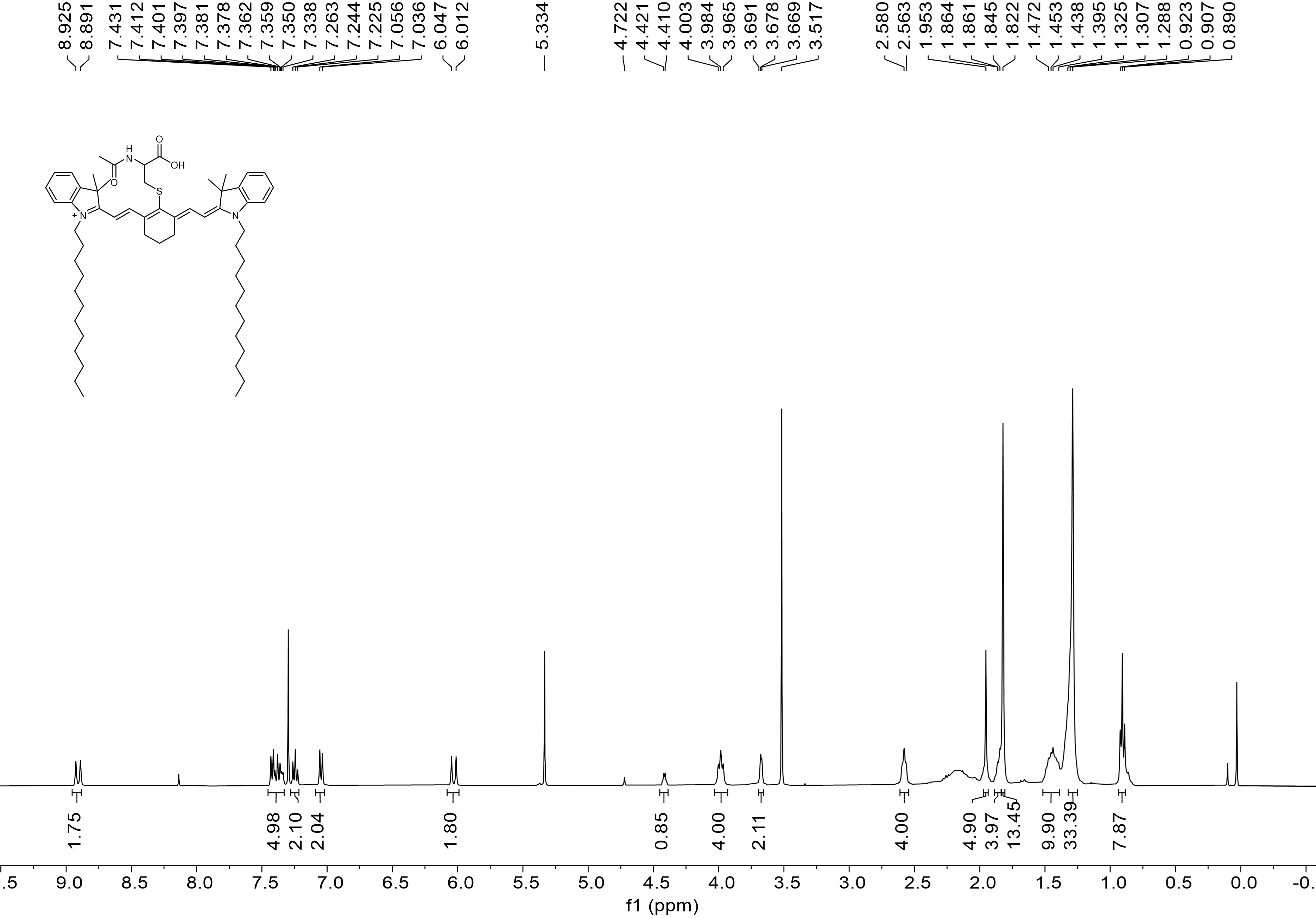


Chromatogram of IR-6B12Ac


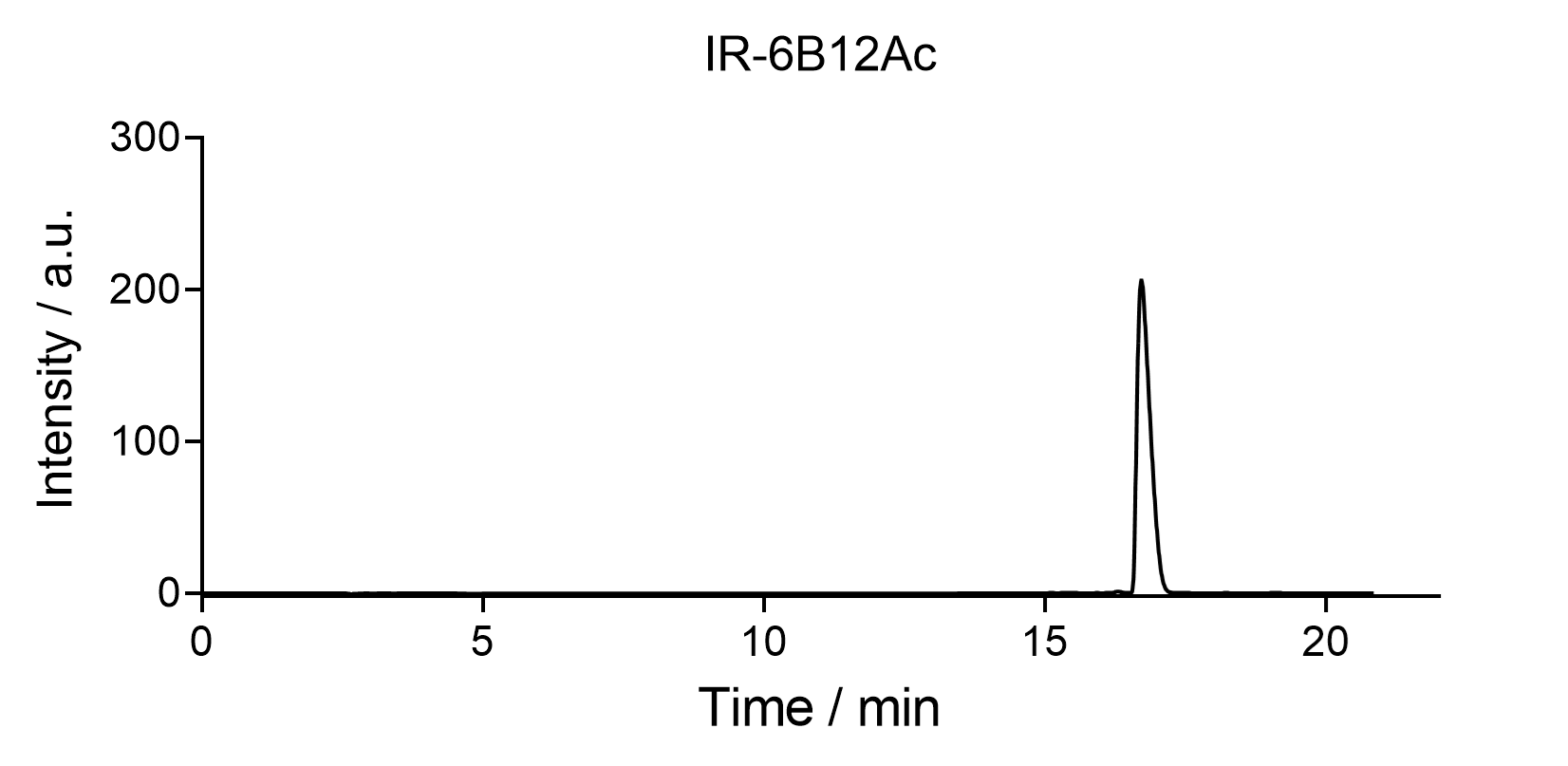


MALDI-TOF-MS spectra of IR-6B12Ac


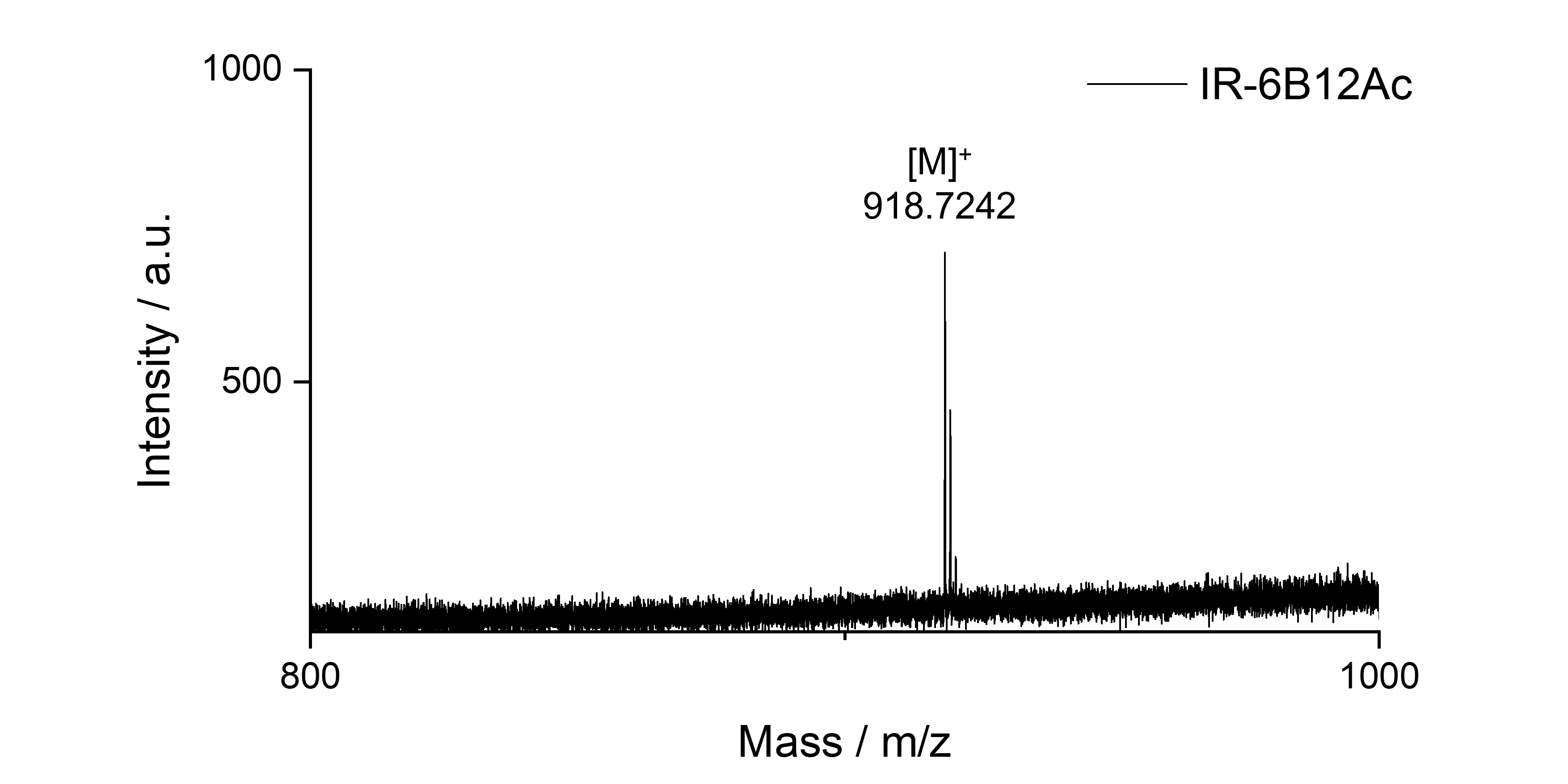


^1^H NMR spectra of FlavAc


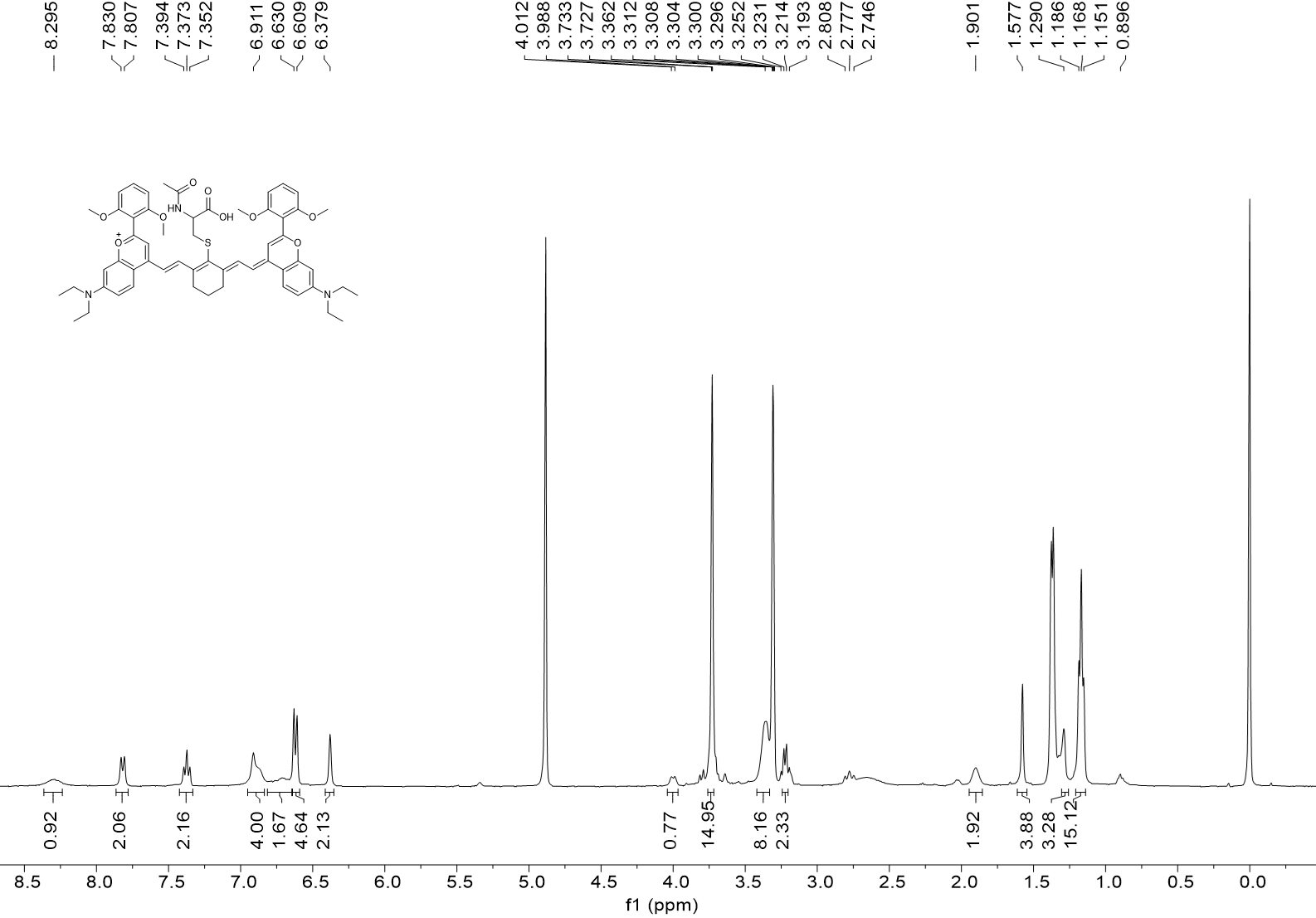


Chromatogram of FlavAc


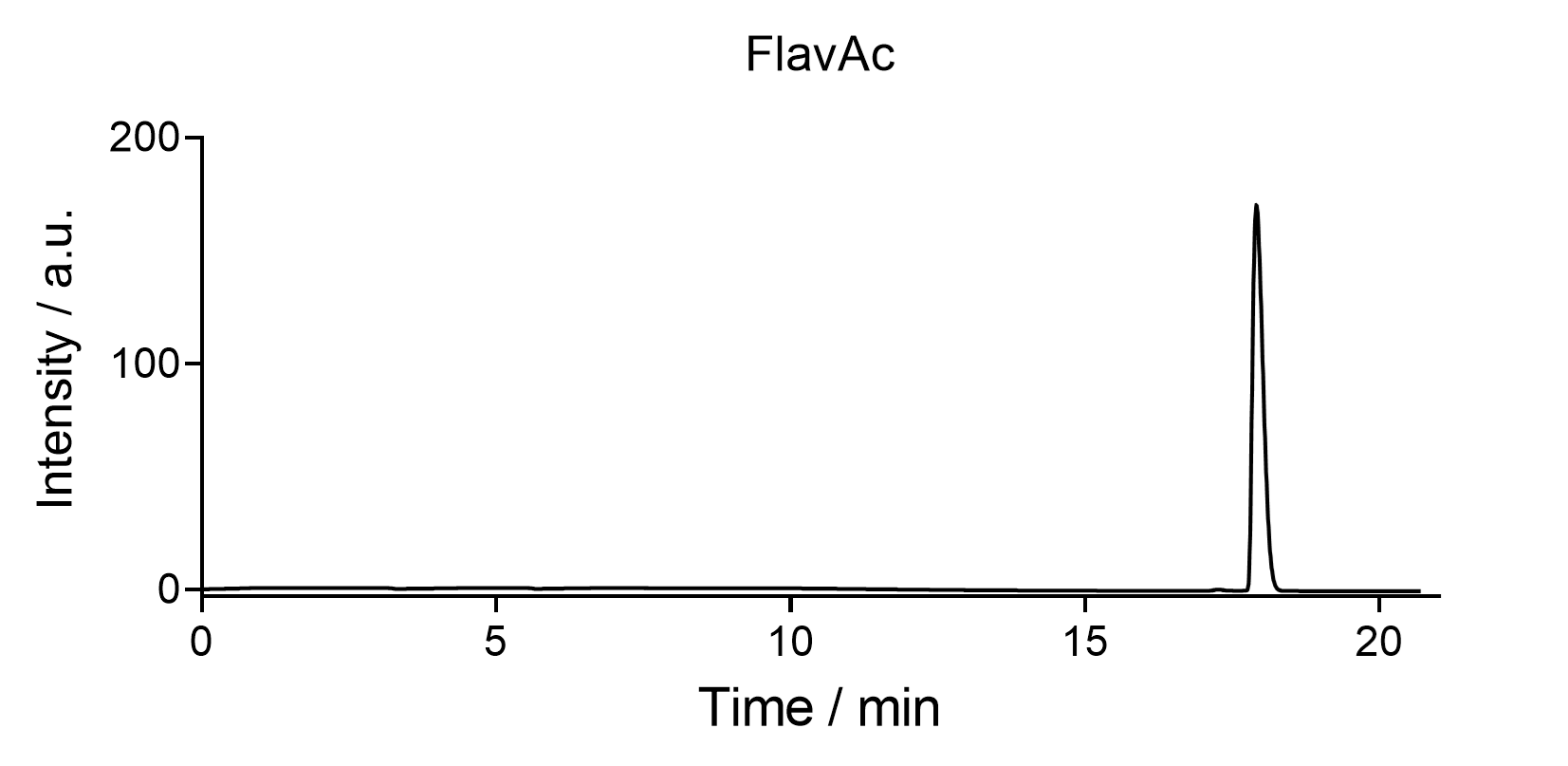


LC-HRMS spectra of FlavAc


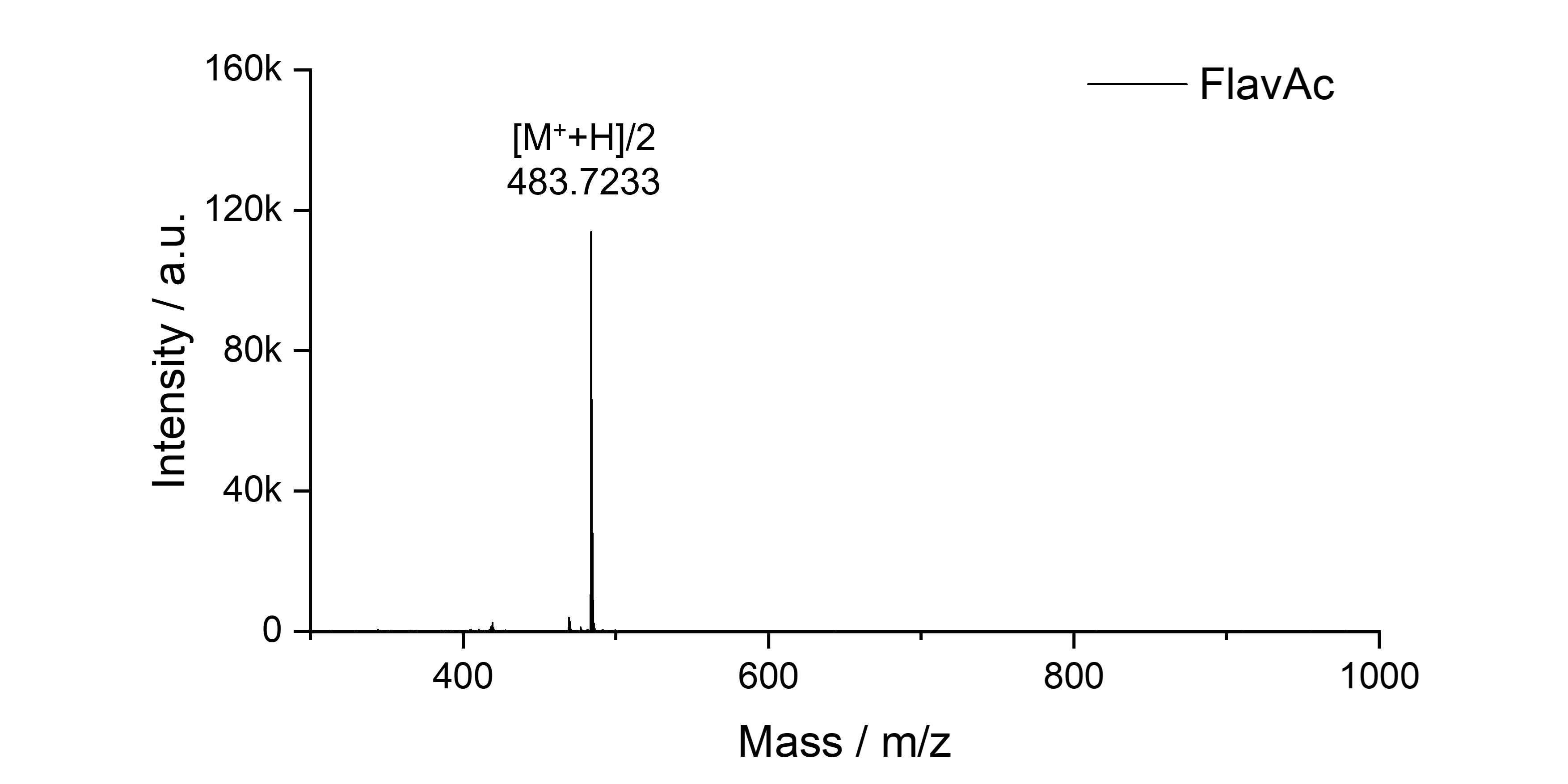


^1^H NMR spectra of PhFlavAc


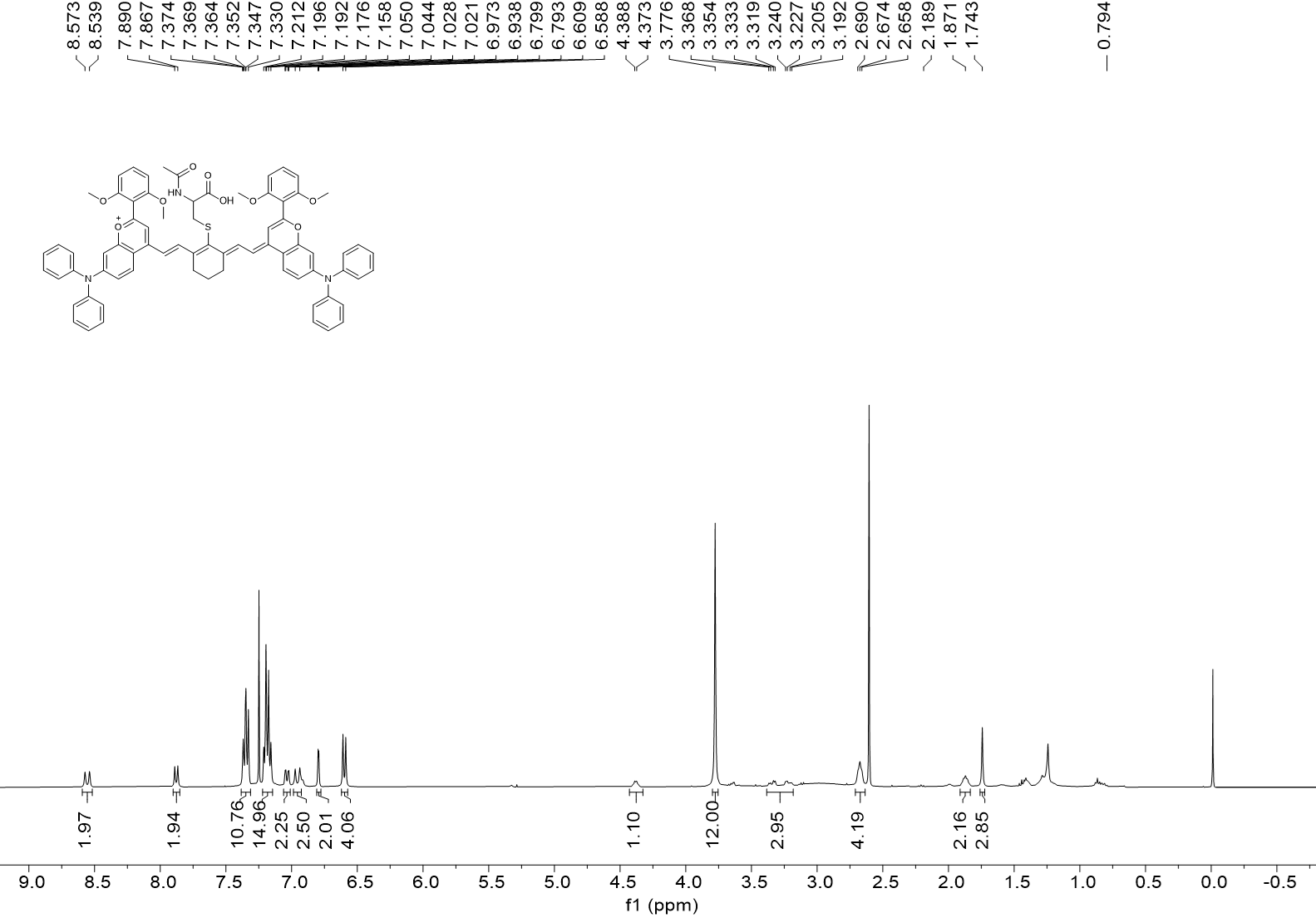


Chromatogram of PhFlavAc


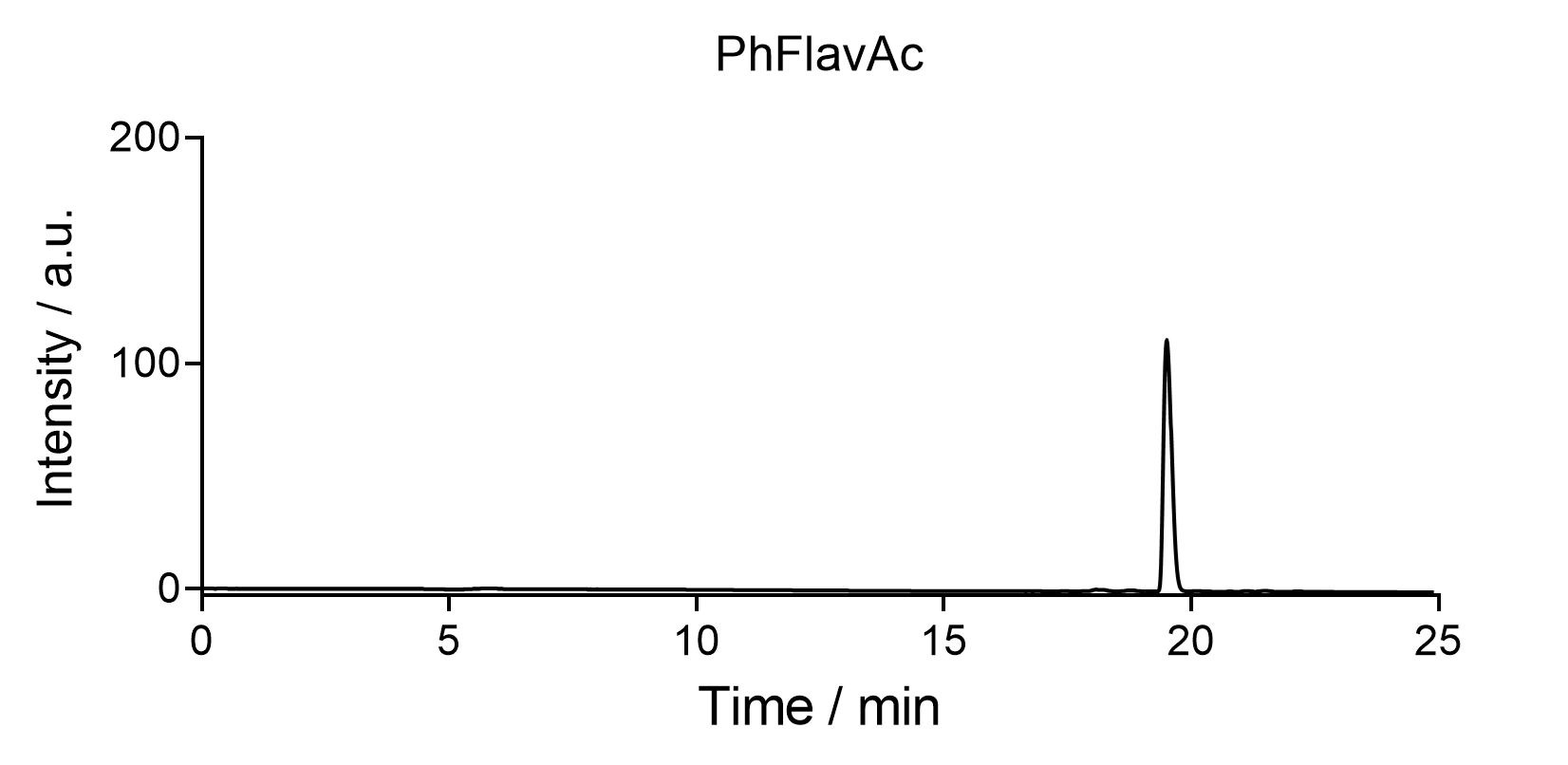


MALDI-TOF-MS spectra of PhFlavAc


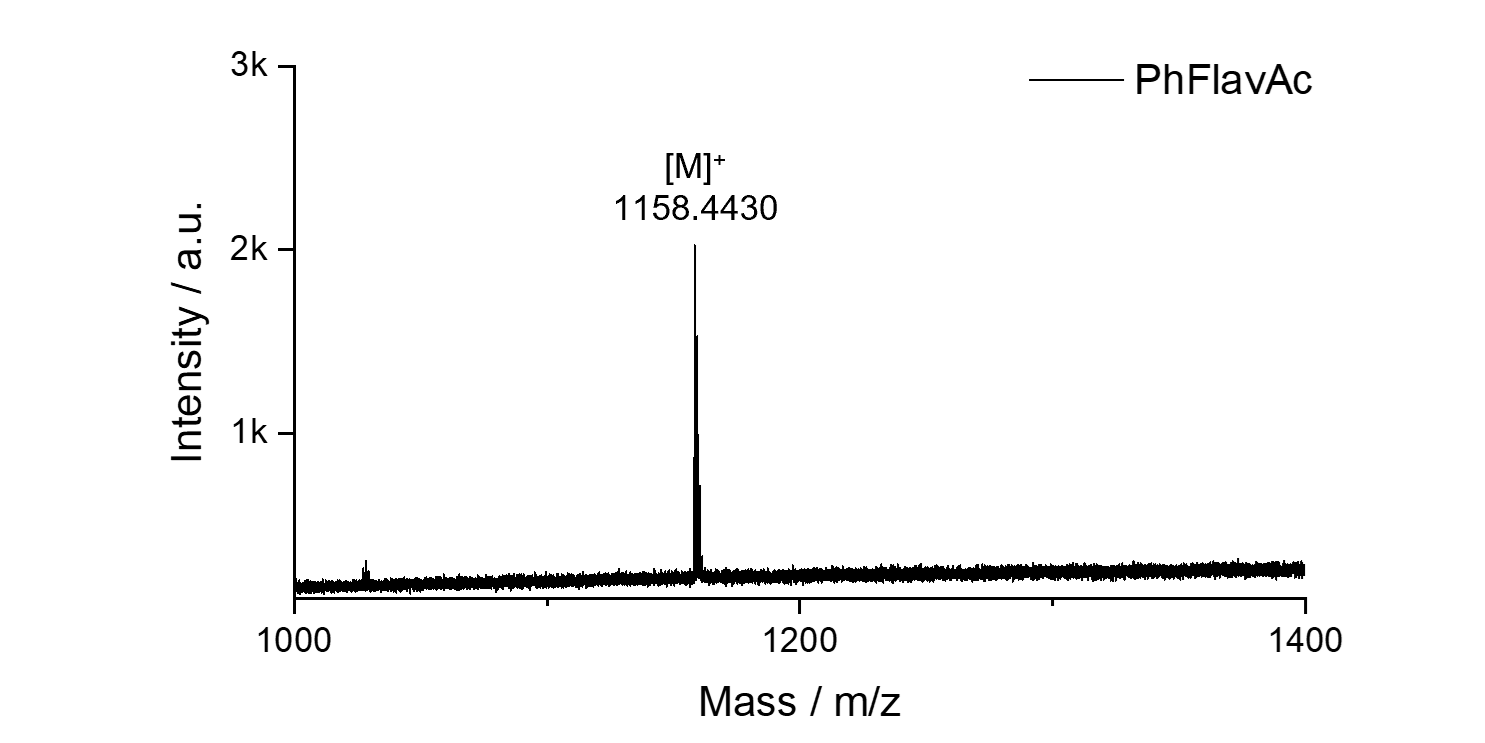


^1^H NMR spectra of FlavBu


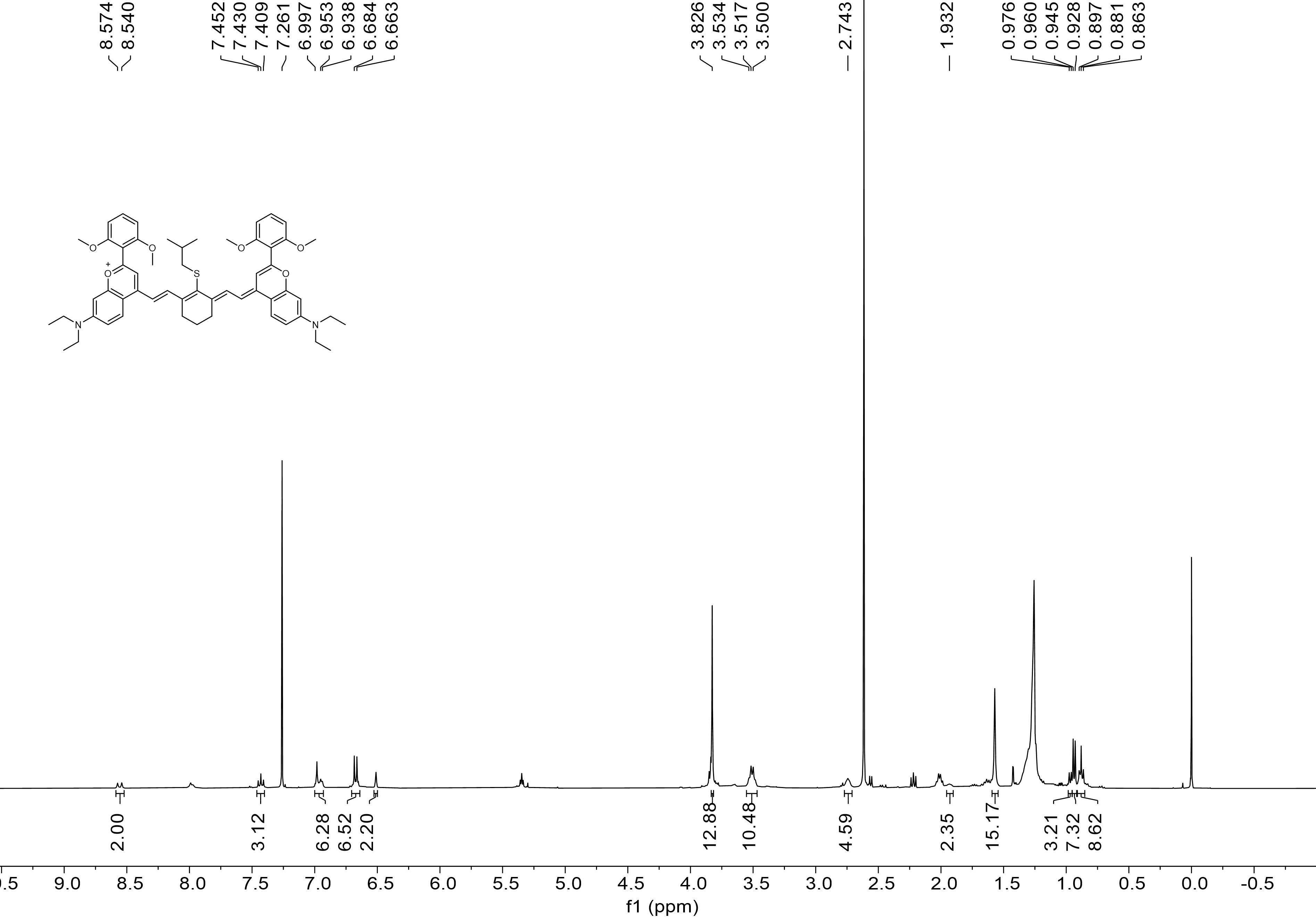


Chromatogram of FlavBu


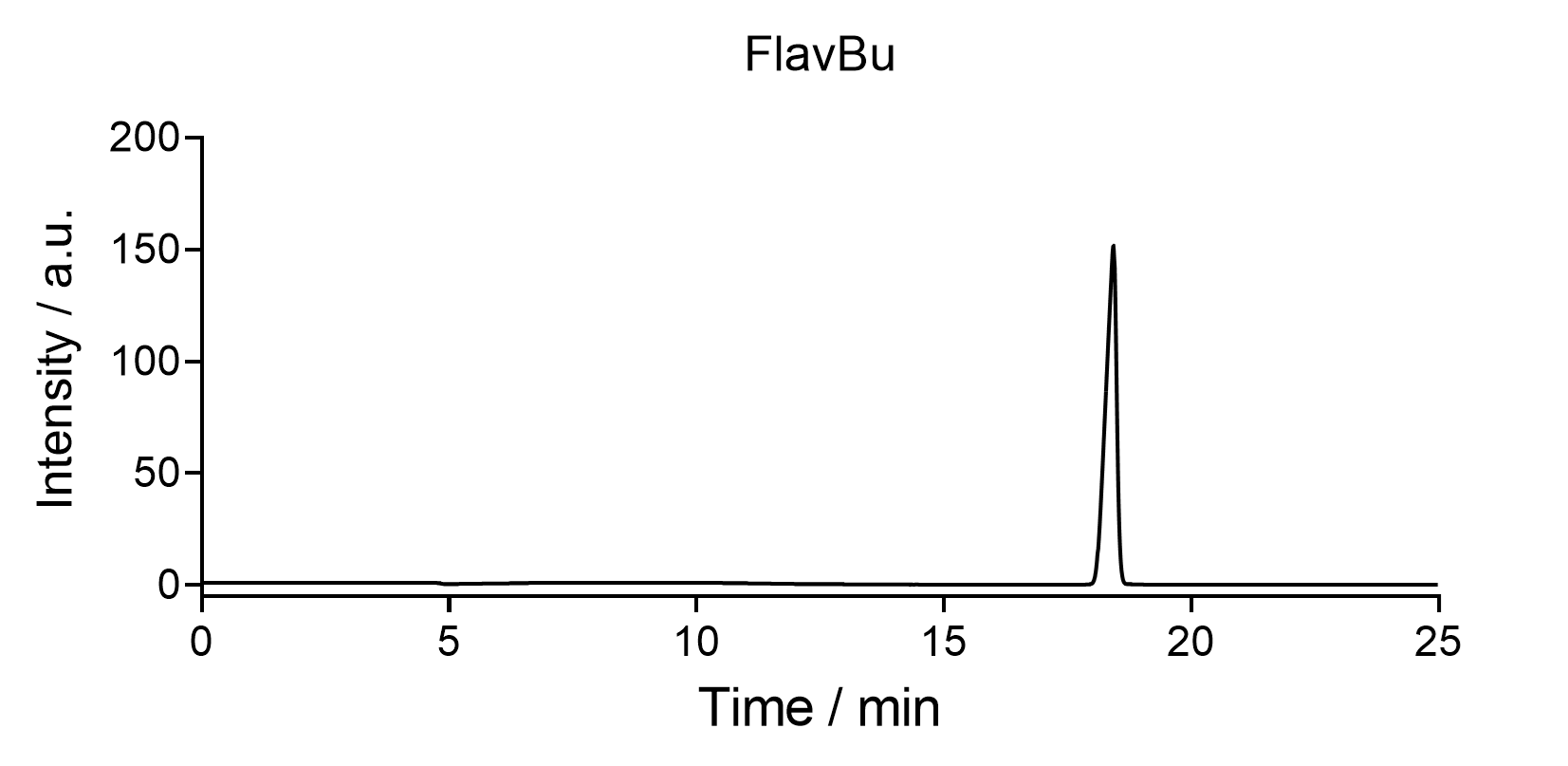


LC-HRMS spectra of FlavBu


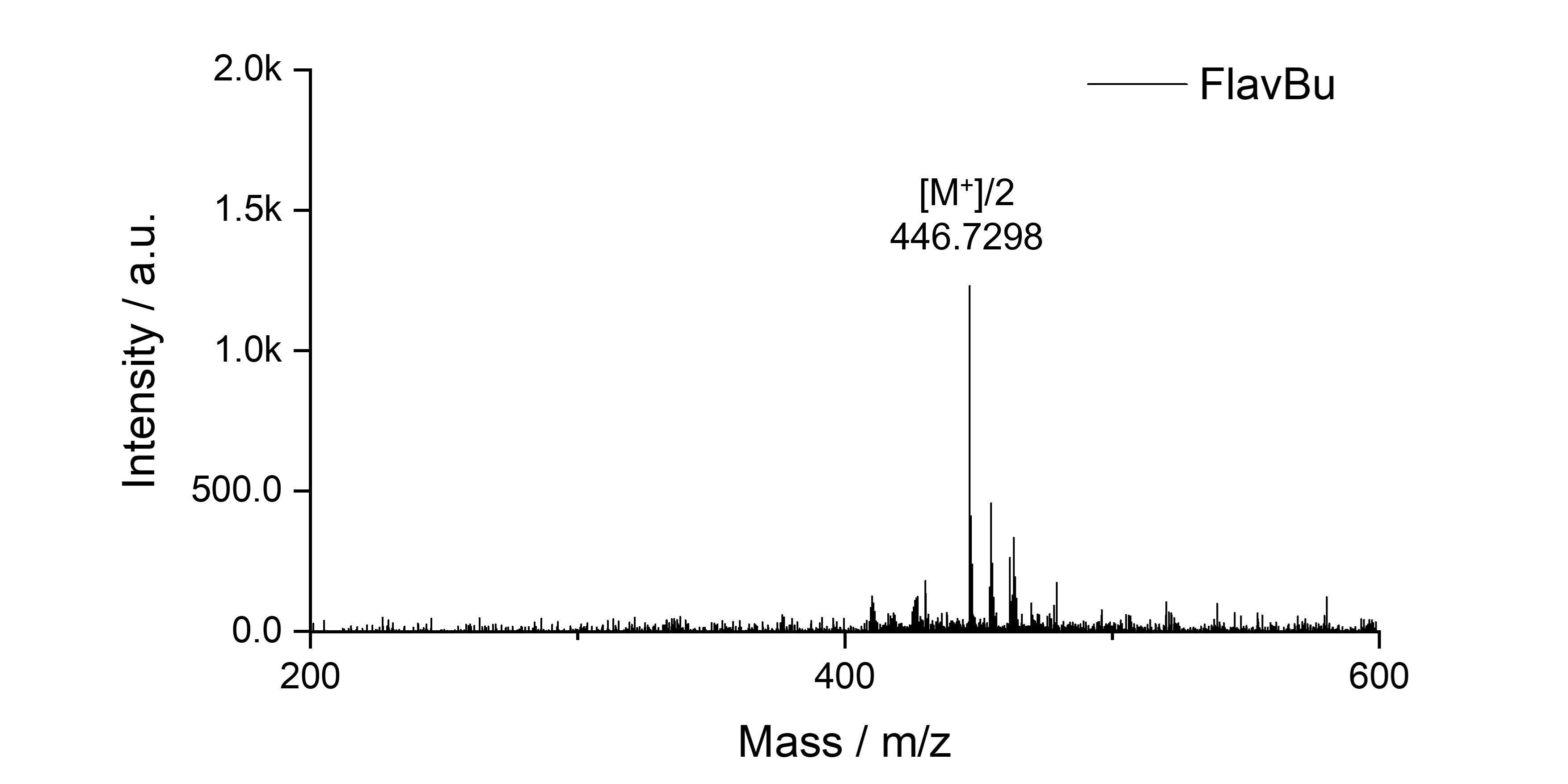


^1^H NMR spectra of FlavRGD


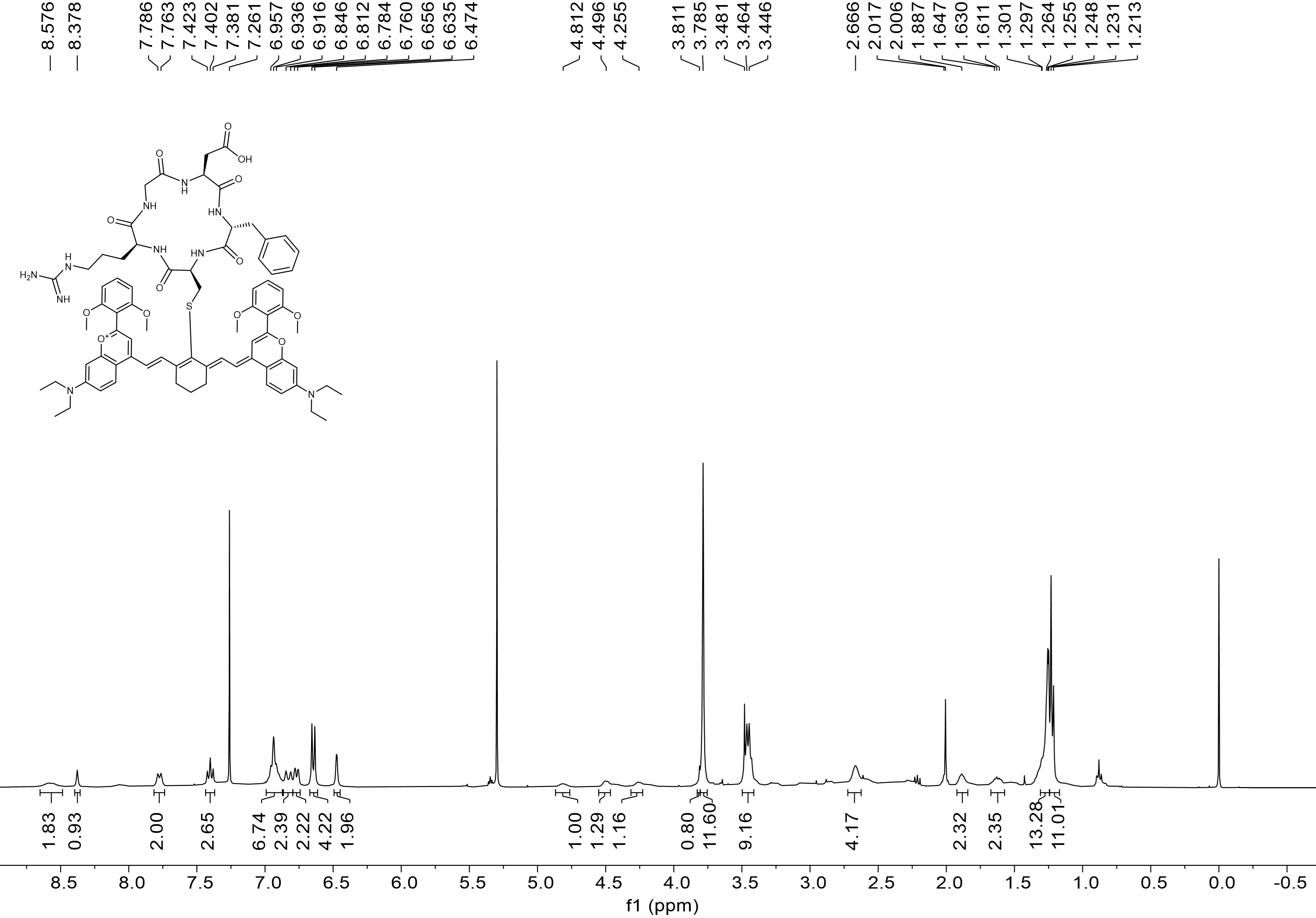


Chromatogram of FlavRGD


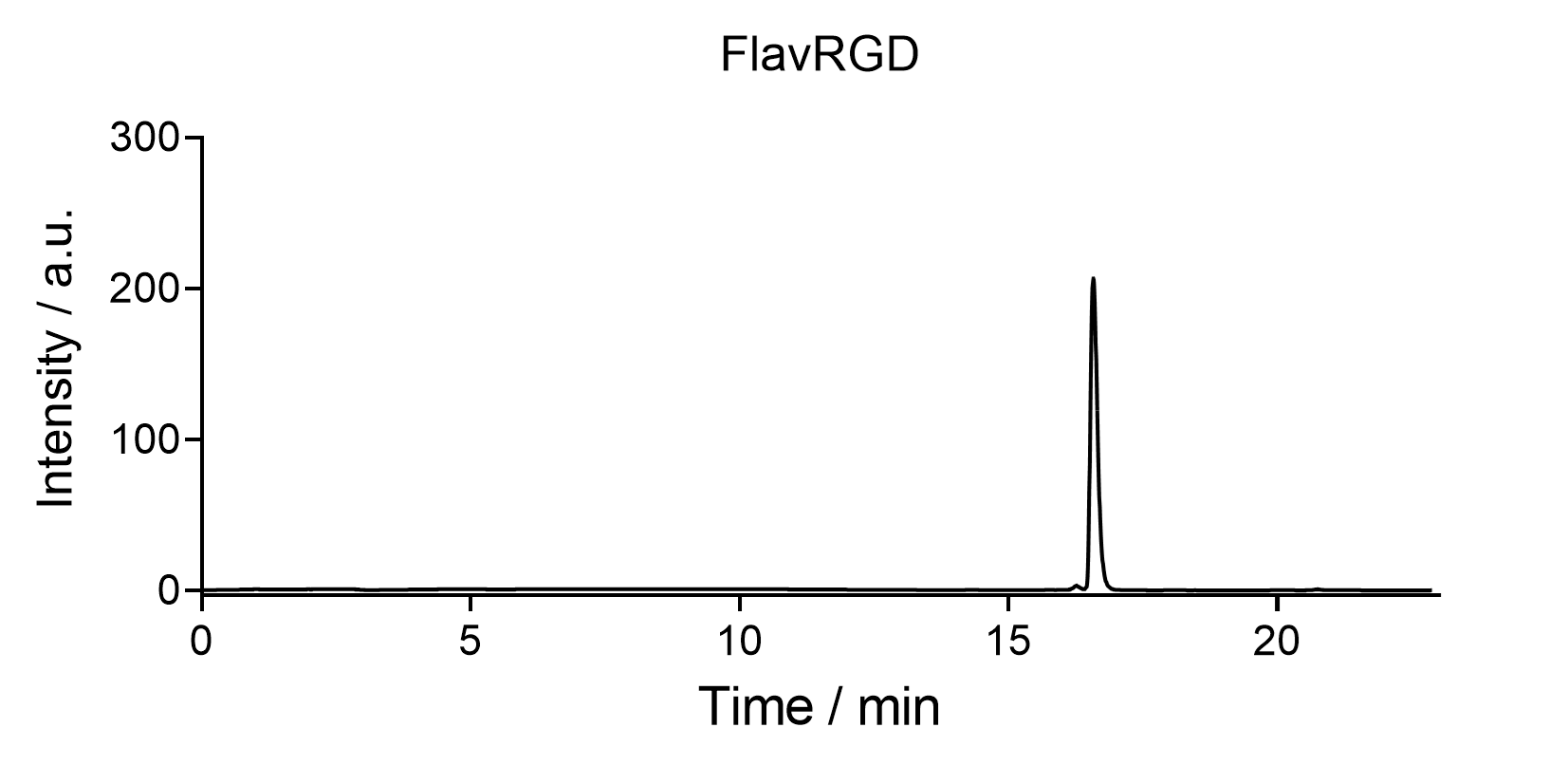


MALDI-TOF-MS spectra of FlavRGD


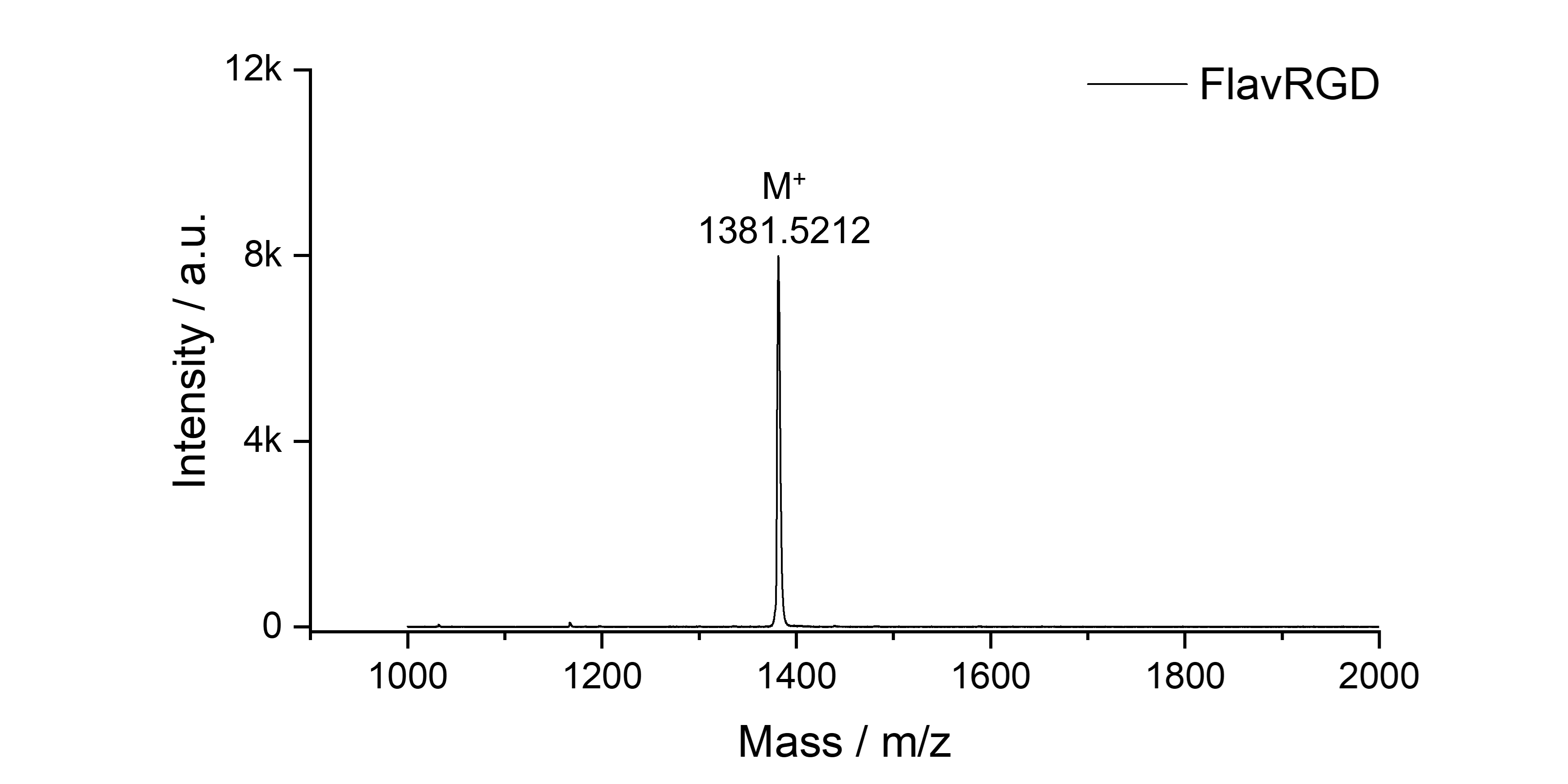


^1^H NMR spectra of FlavCOOH

^
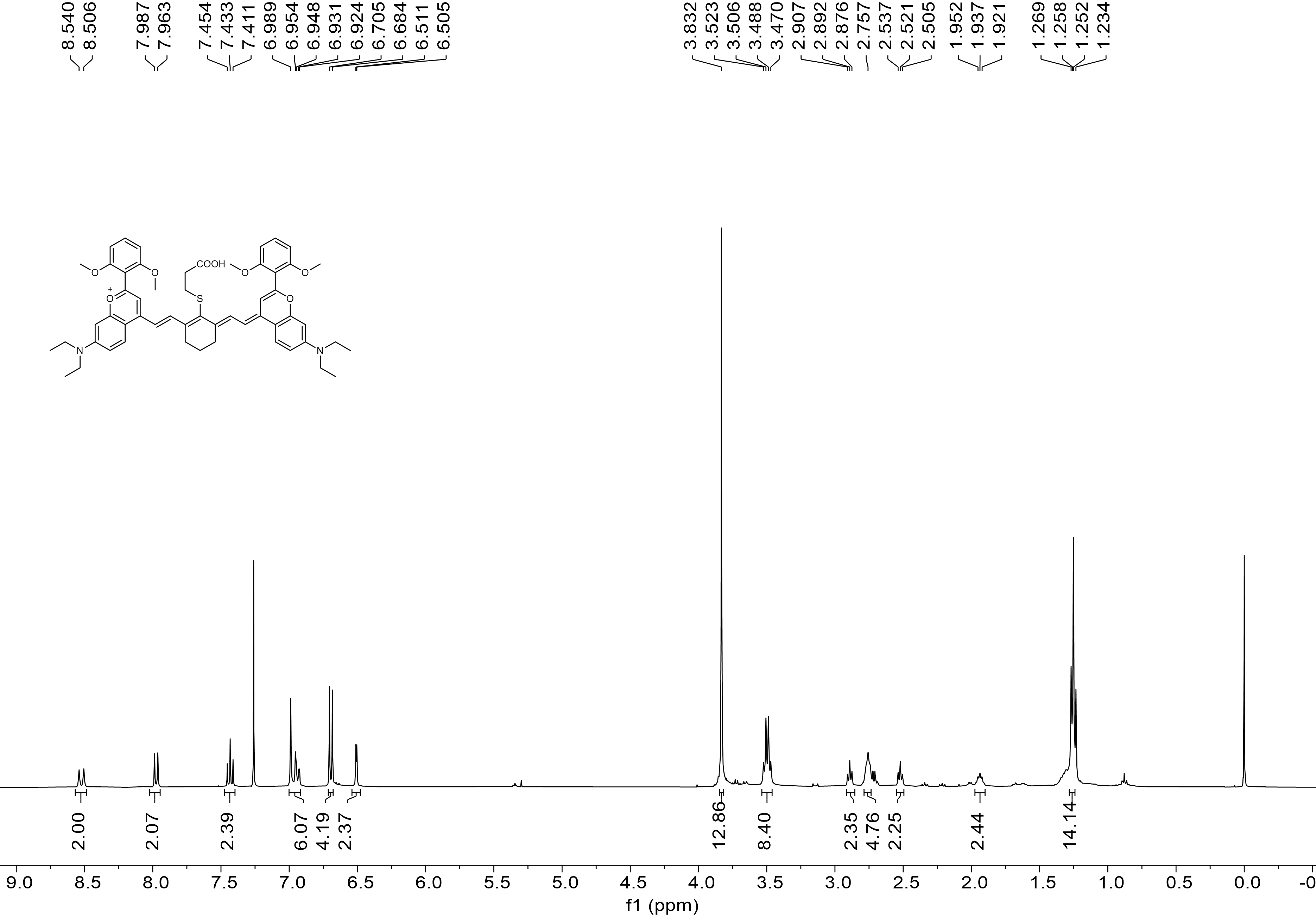
^

^1^H NMR spectra of Flav2


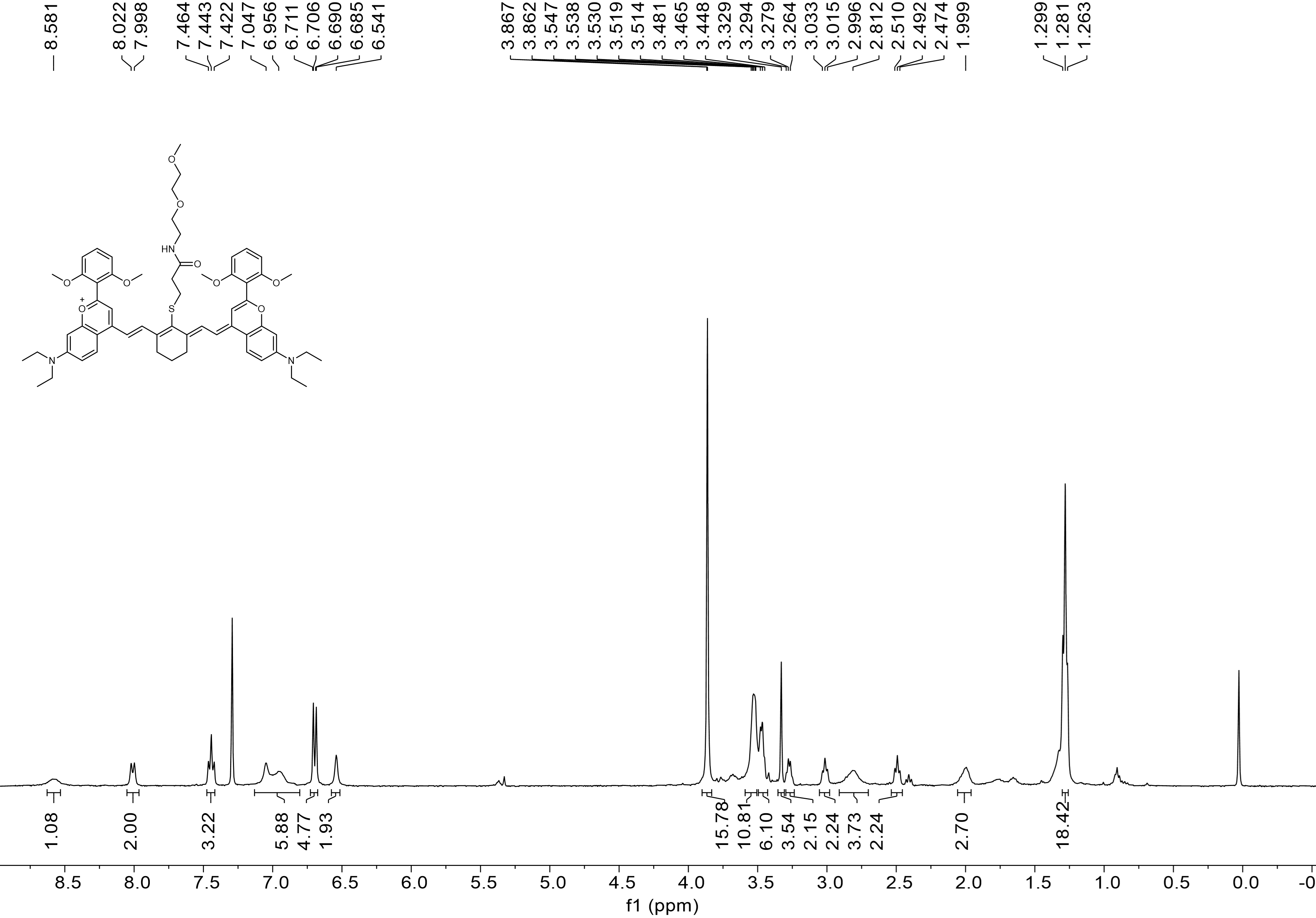


Chromatogram of Flav2


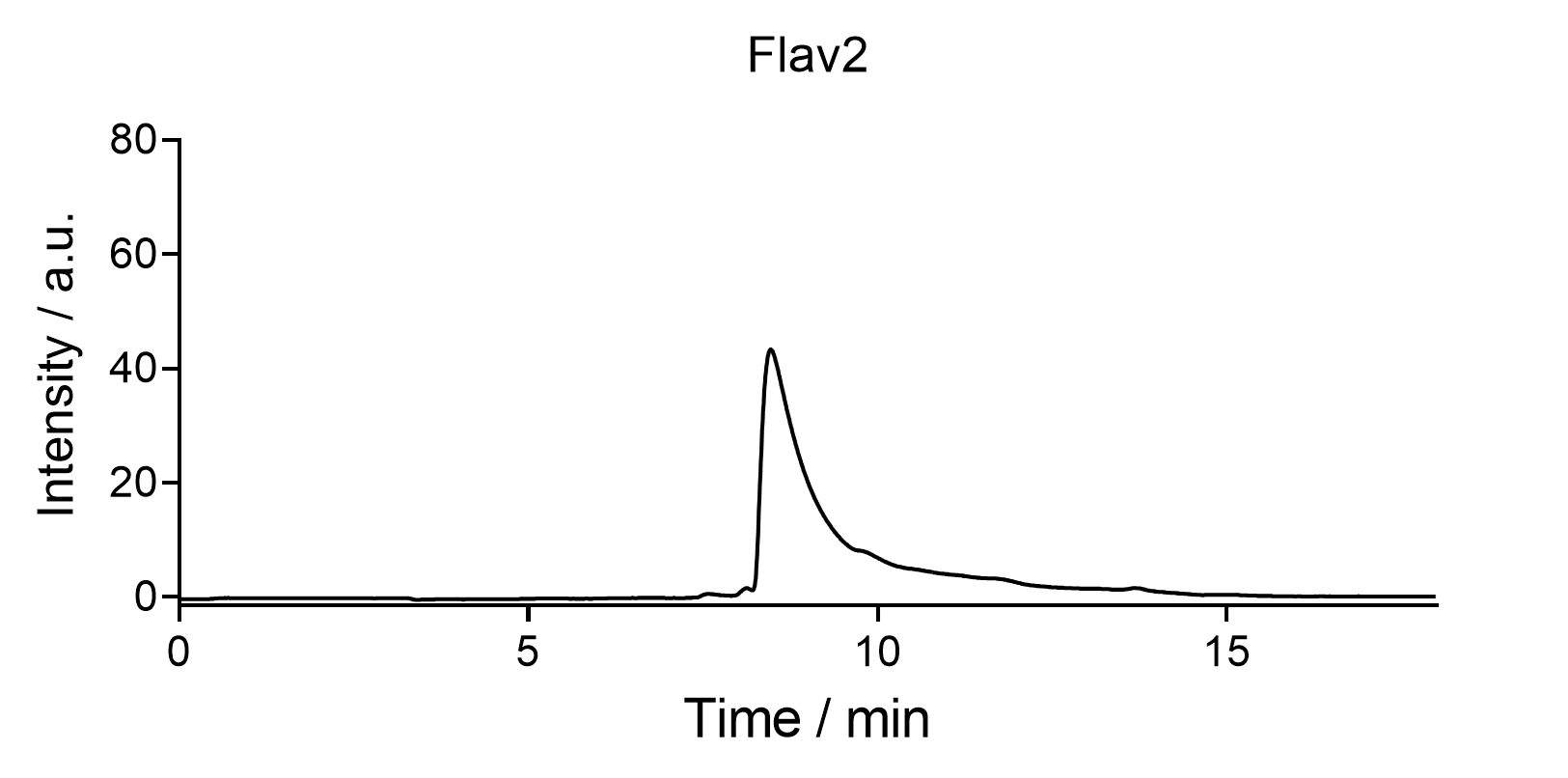


LC-HRMS spectra of Flav2


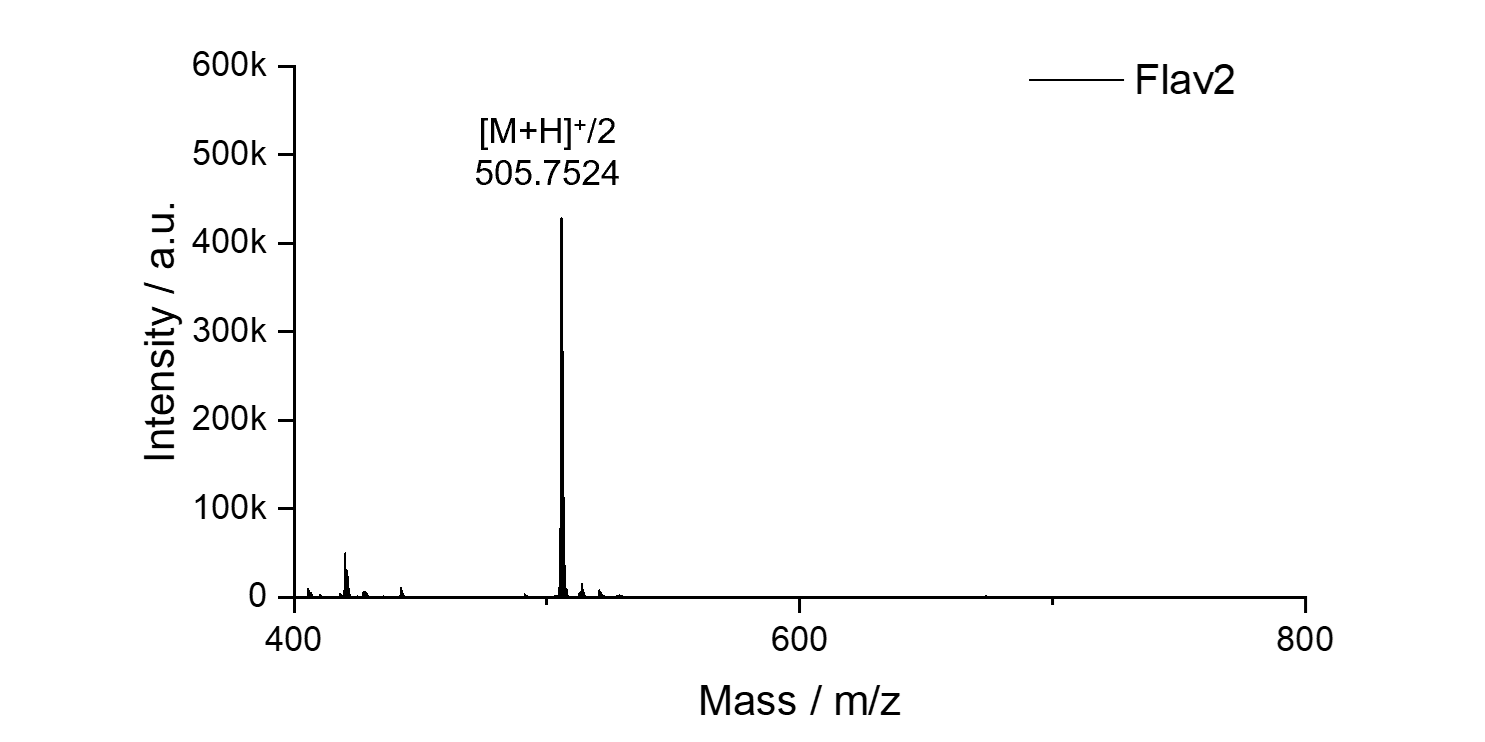


^1^H NMR spectra of Flav3


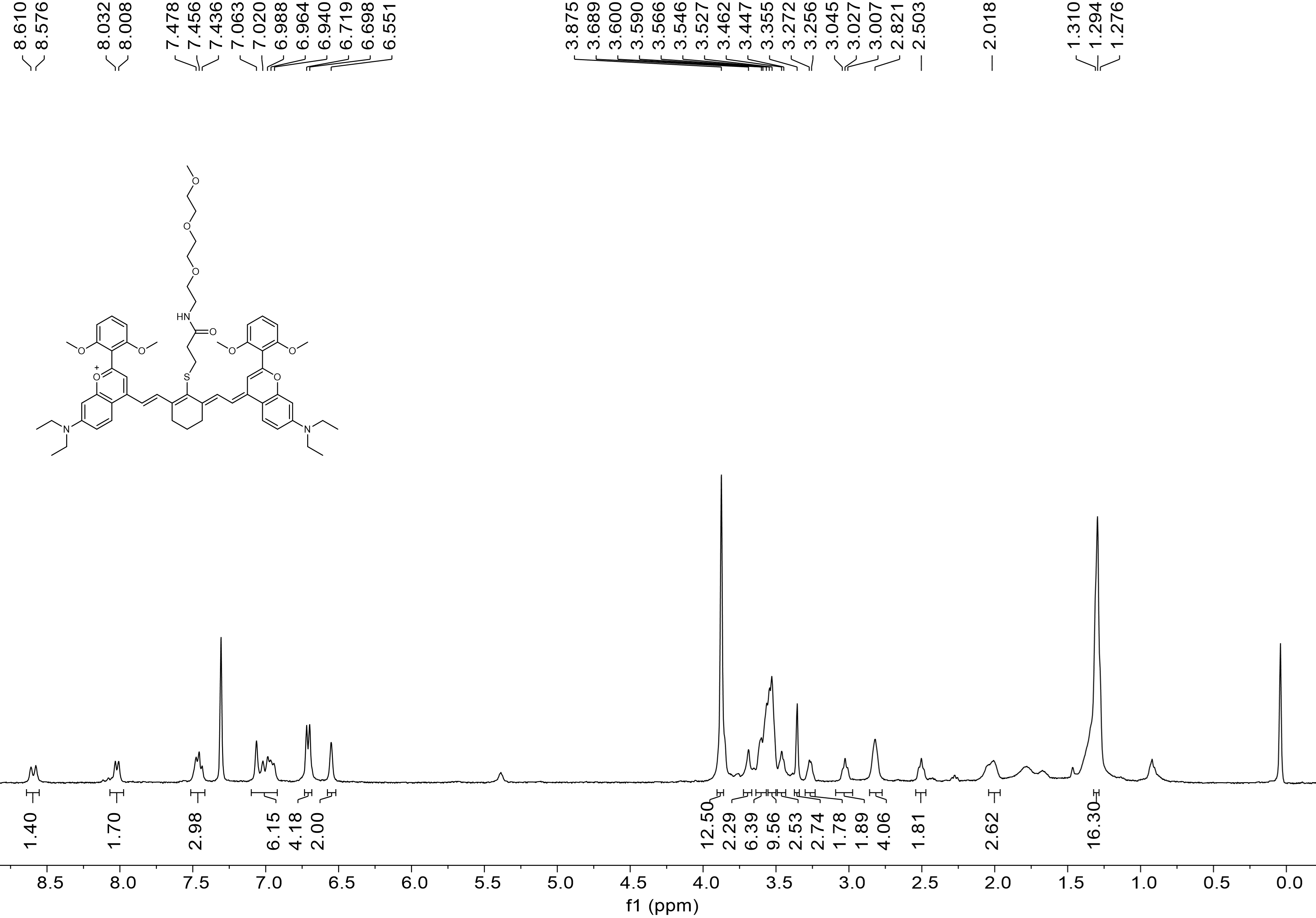


Chromatogram of Flav3


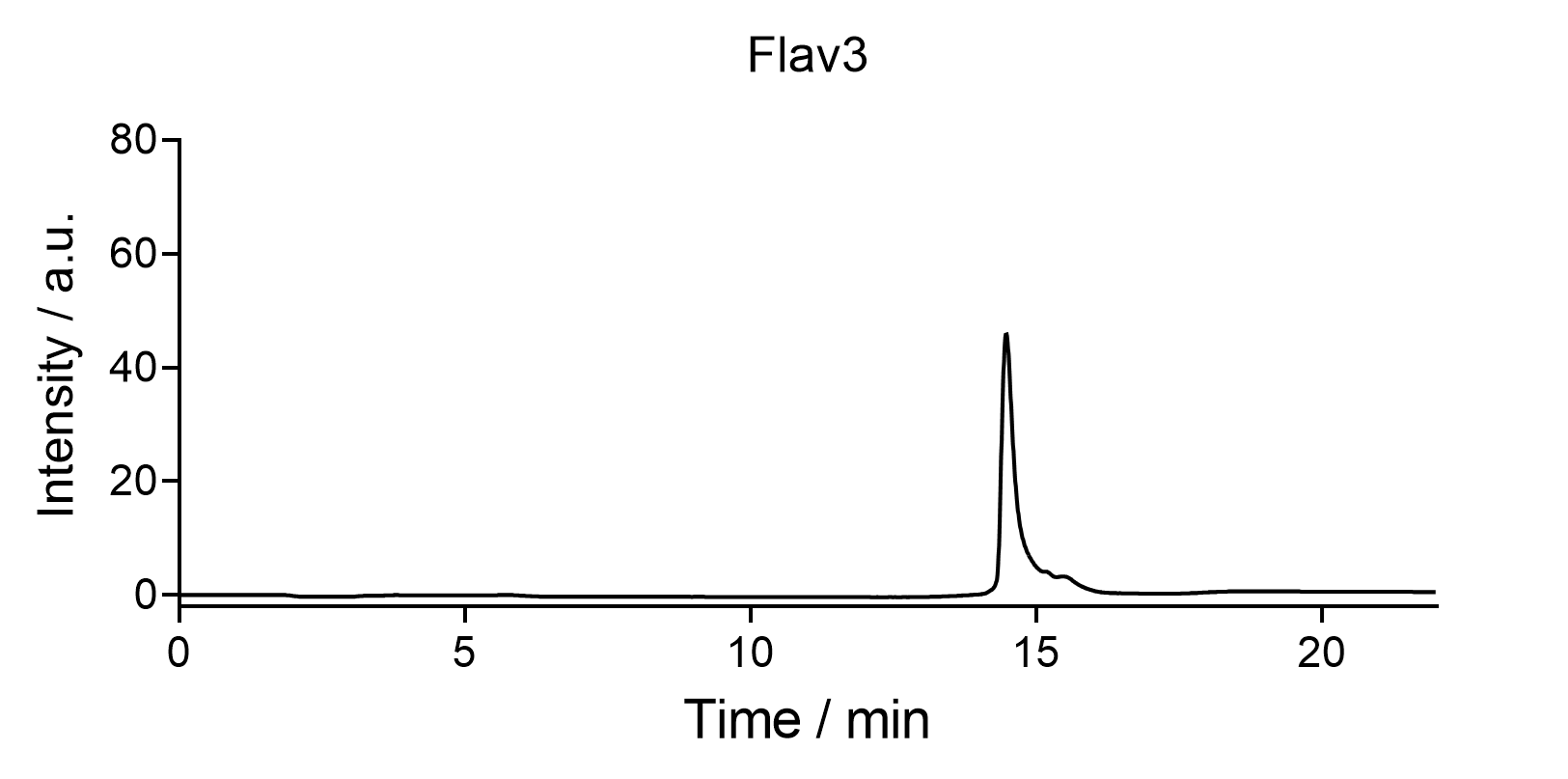


MALDI-TOF-MS spectra of Flav3


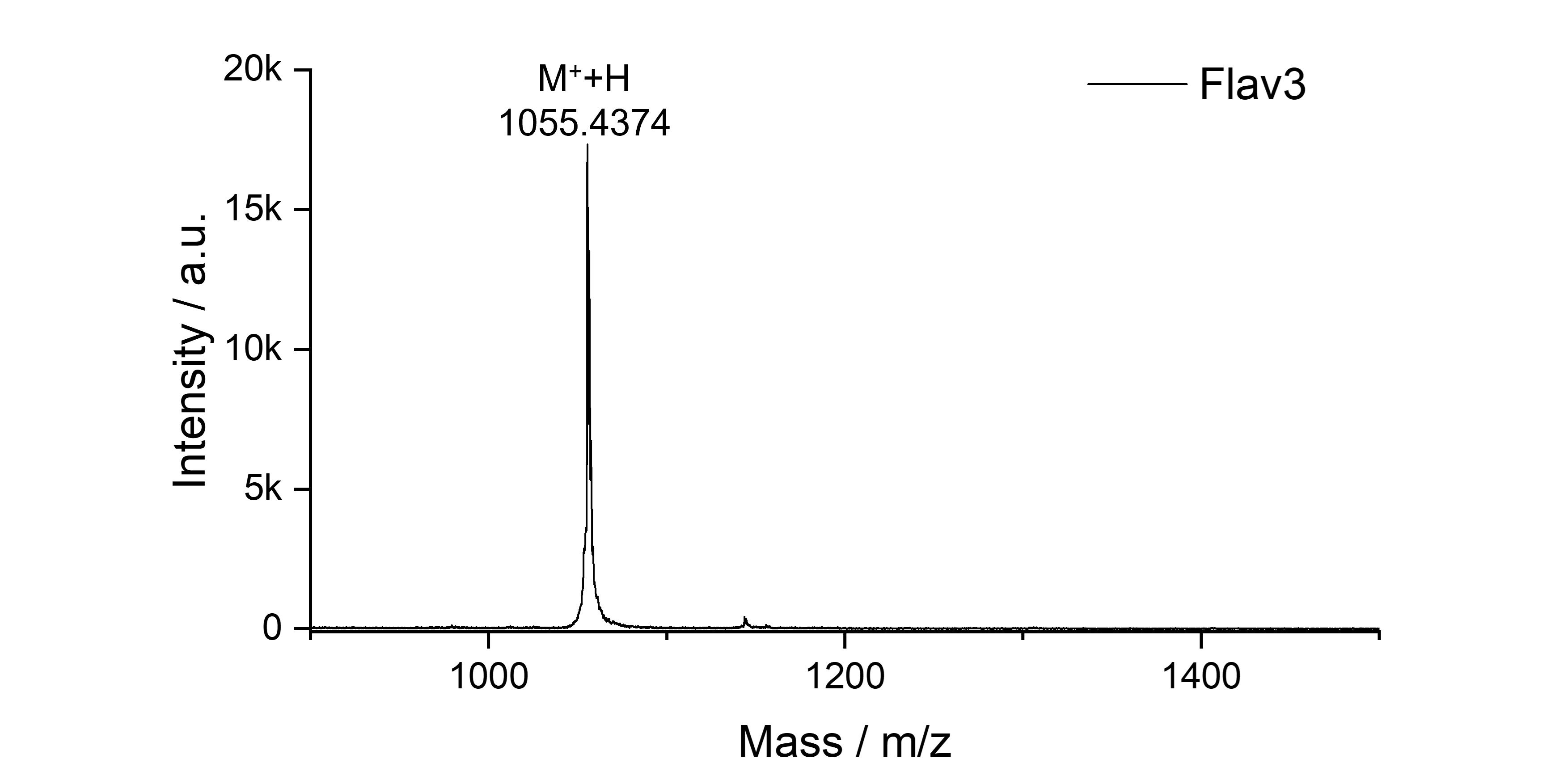


^1^H NMR spectra of Flav7


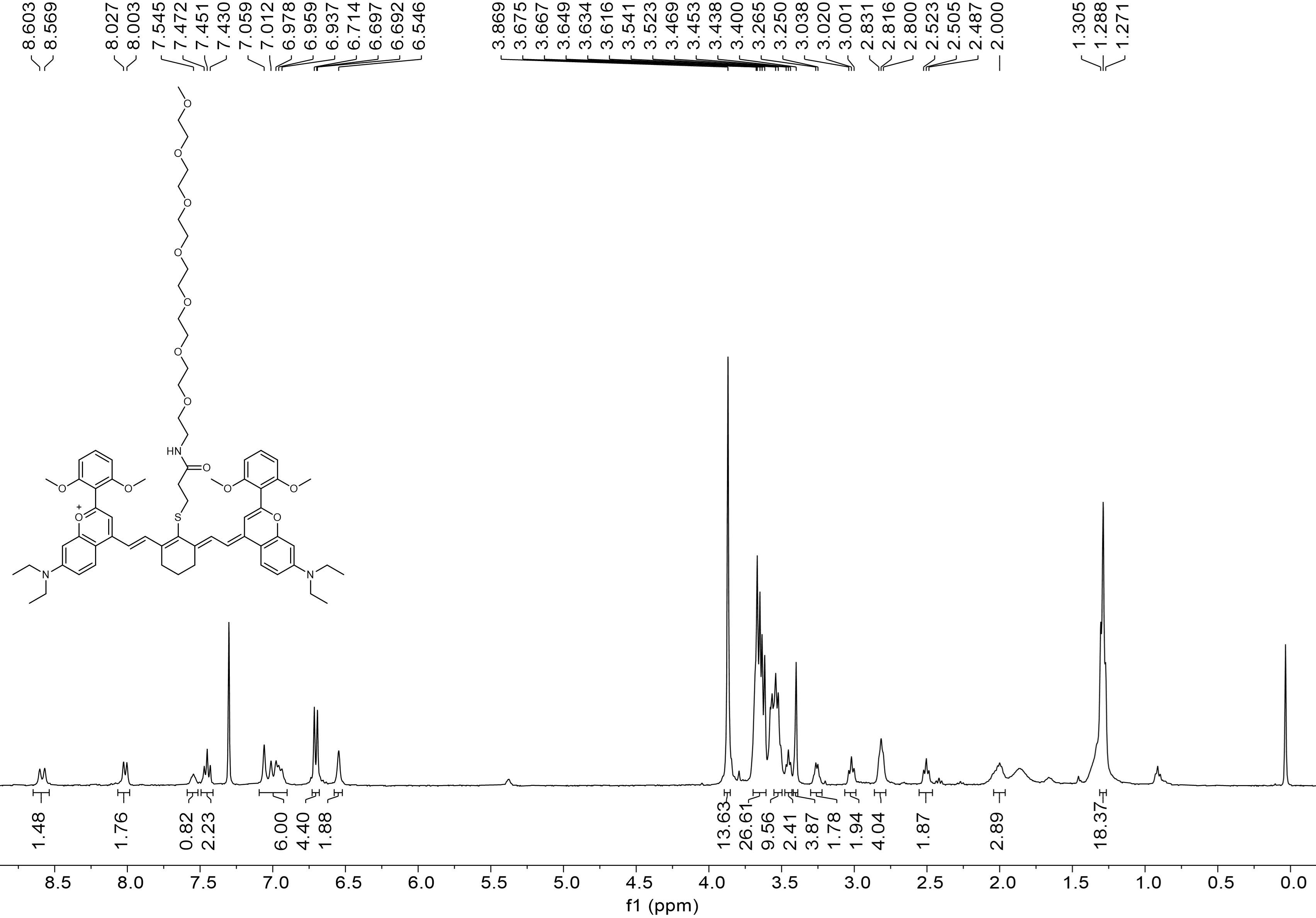


Chromatogram of Flav7


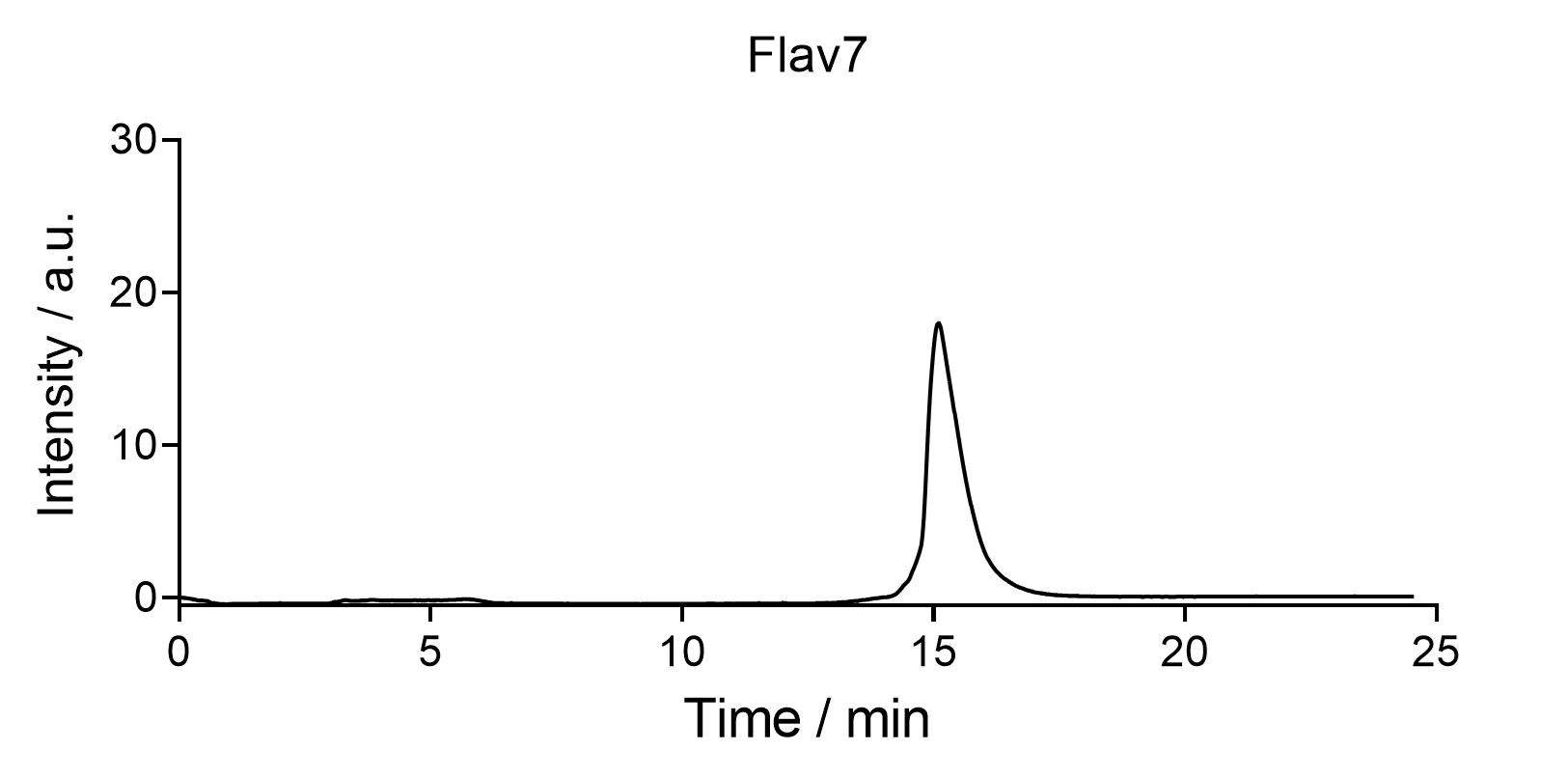


MALDI-TOF-MS spectra of Flav7


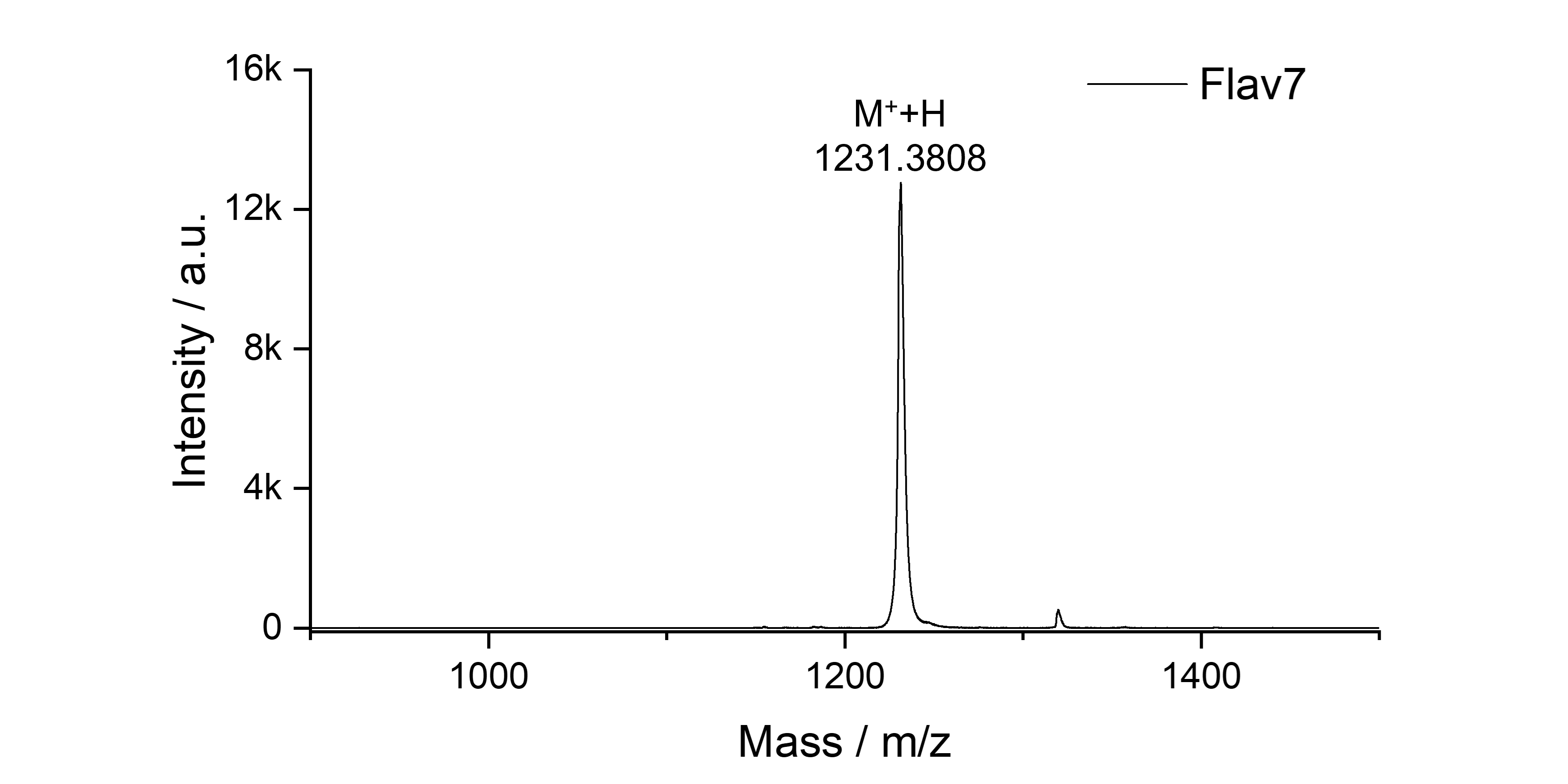


^1^H NMR spectra of Flav9


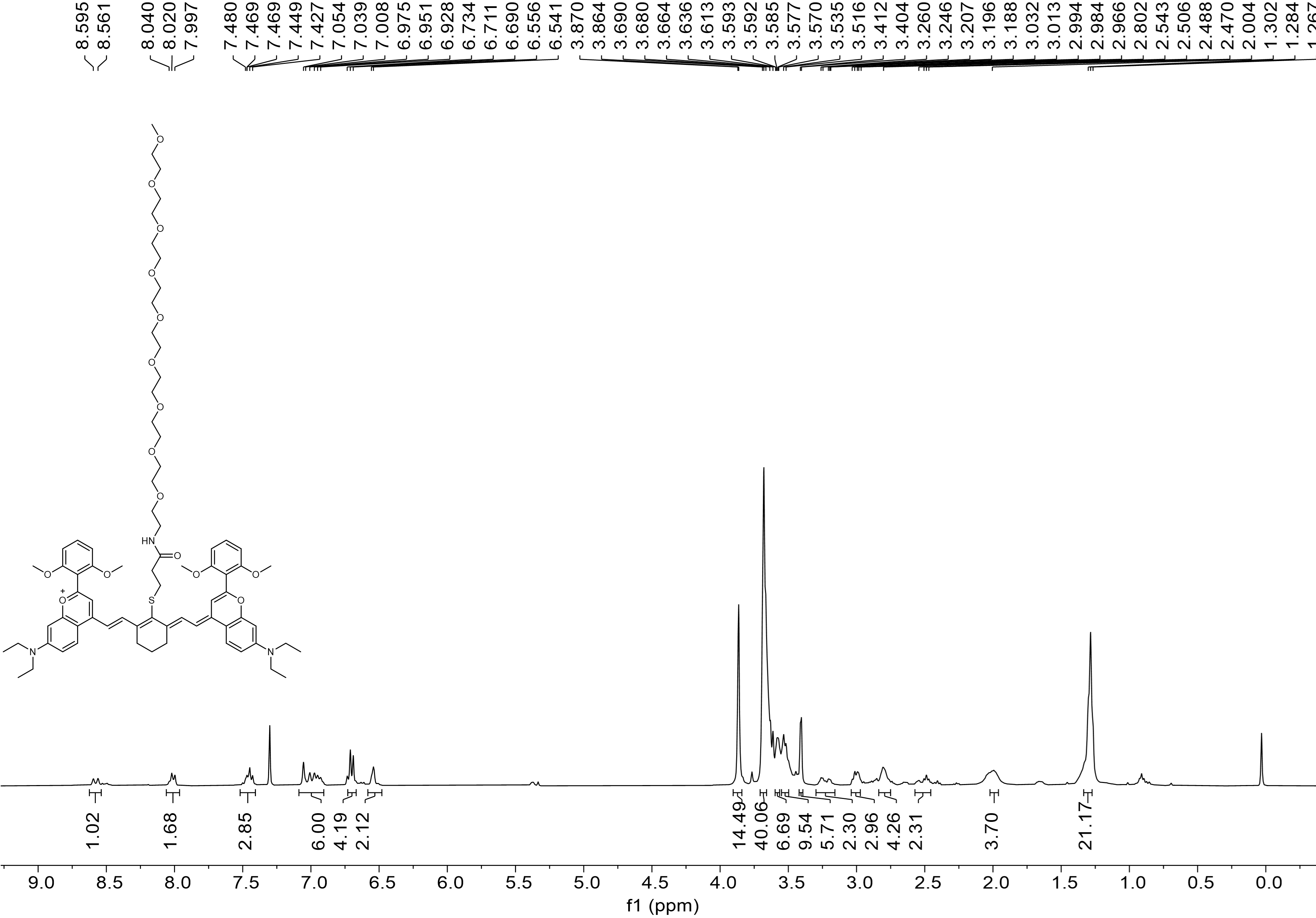


Chromatogram of Flav9


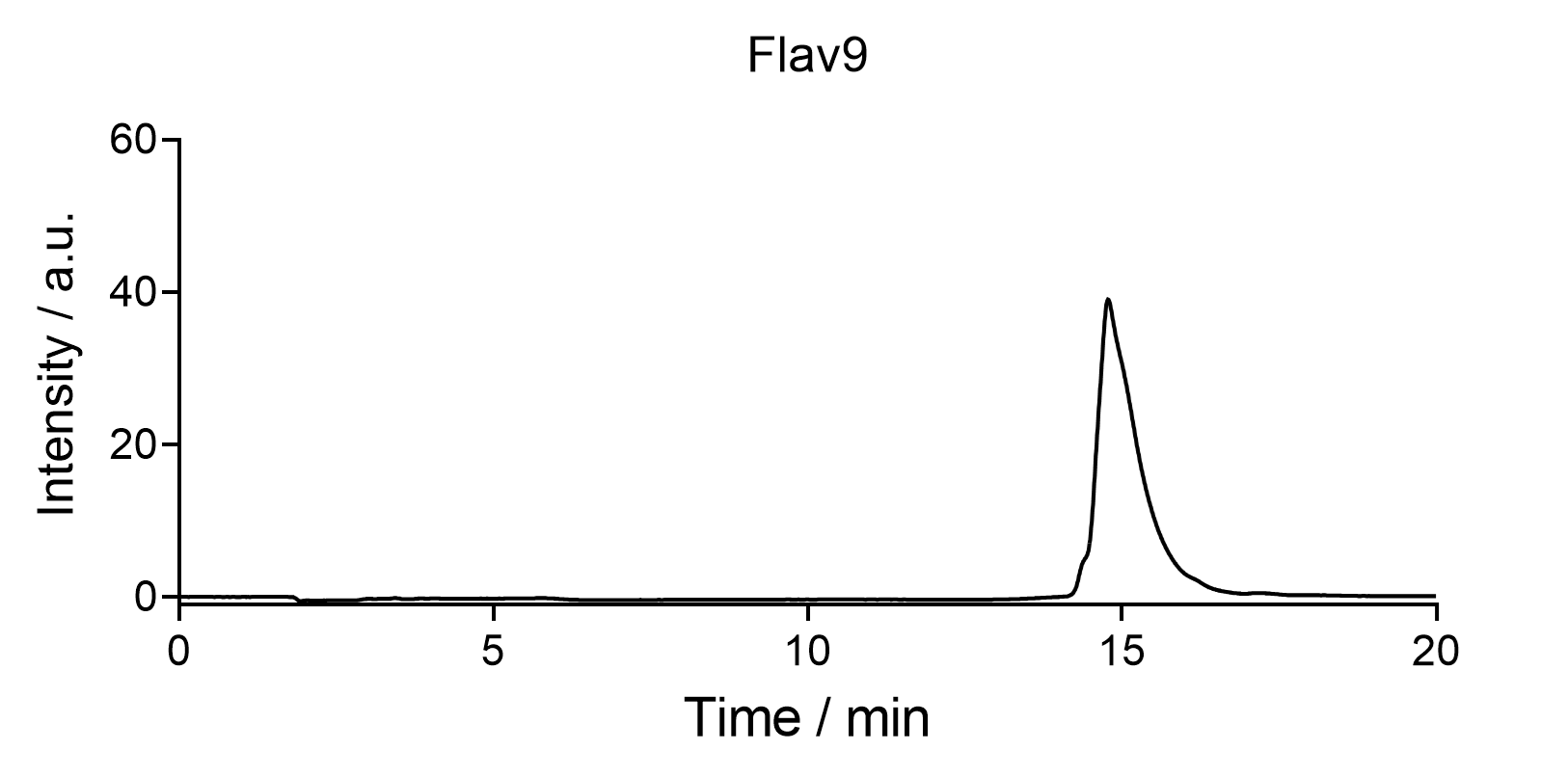


MALDI-TOF-MS spectra of Flav9


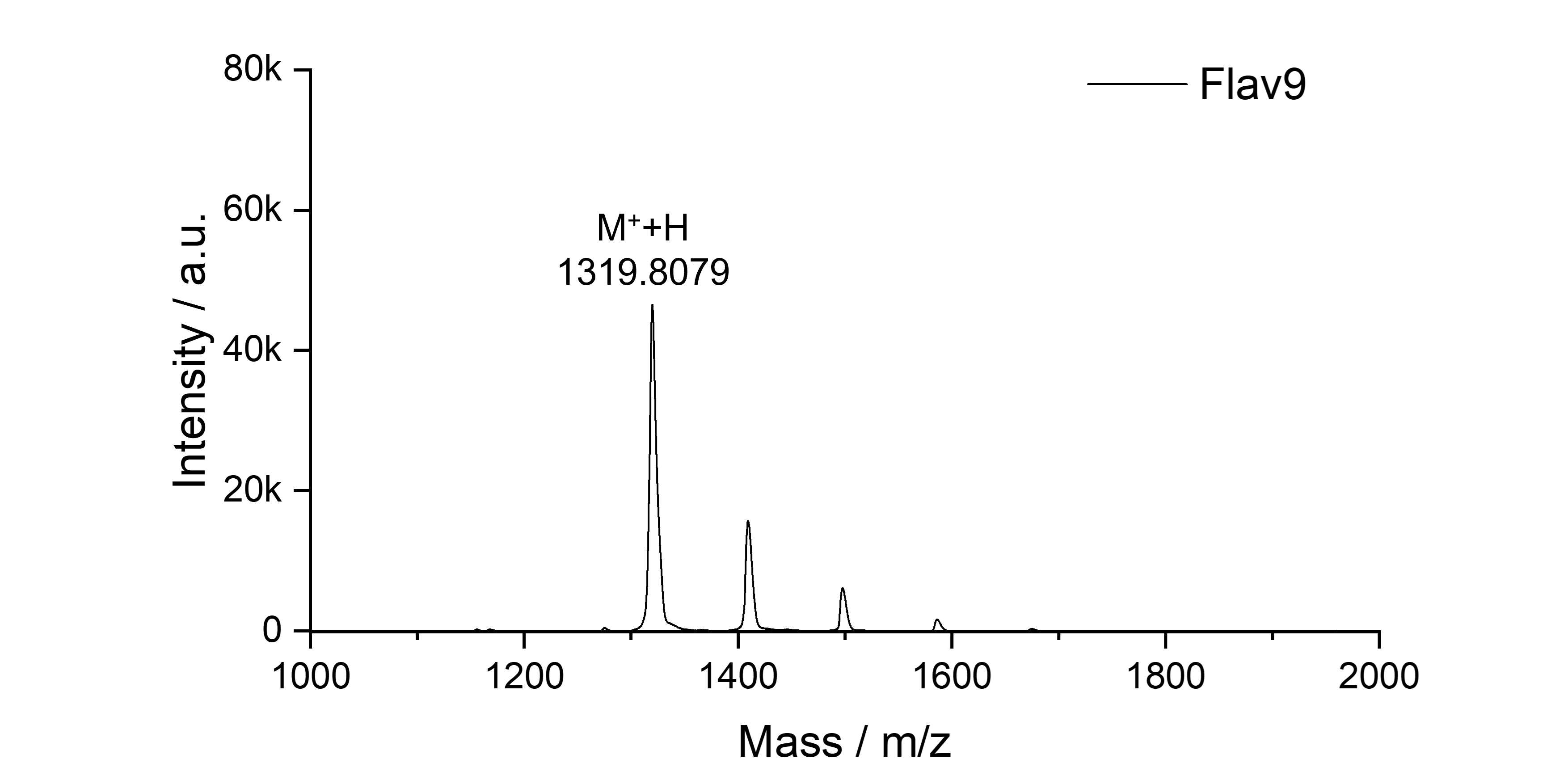


^1^H NMR spectra of Flav12


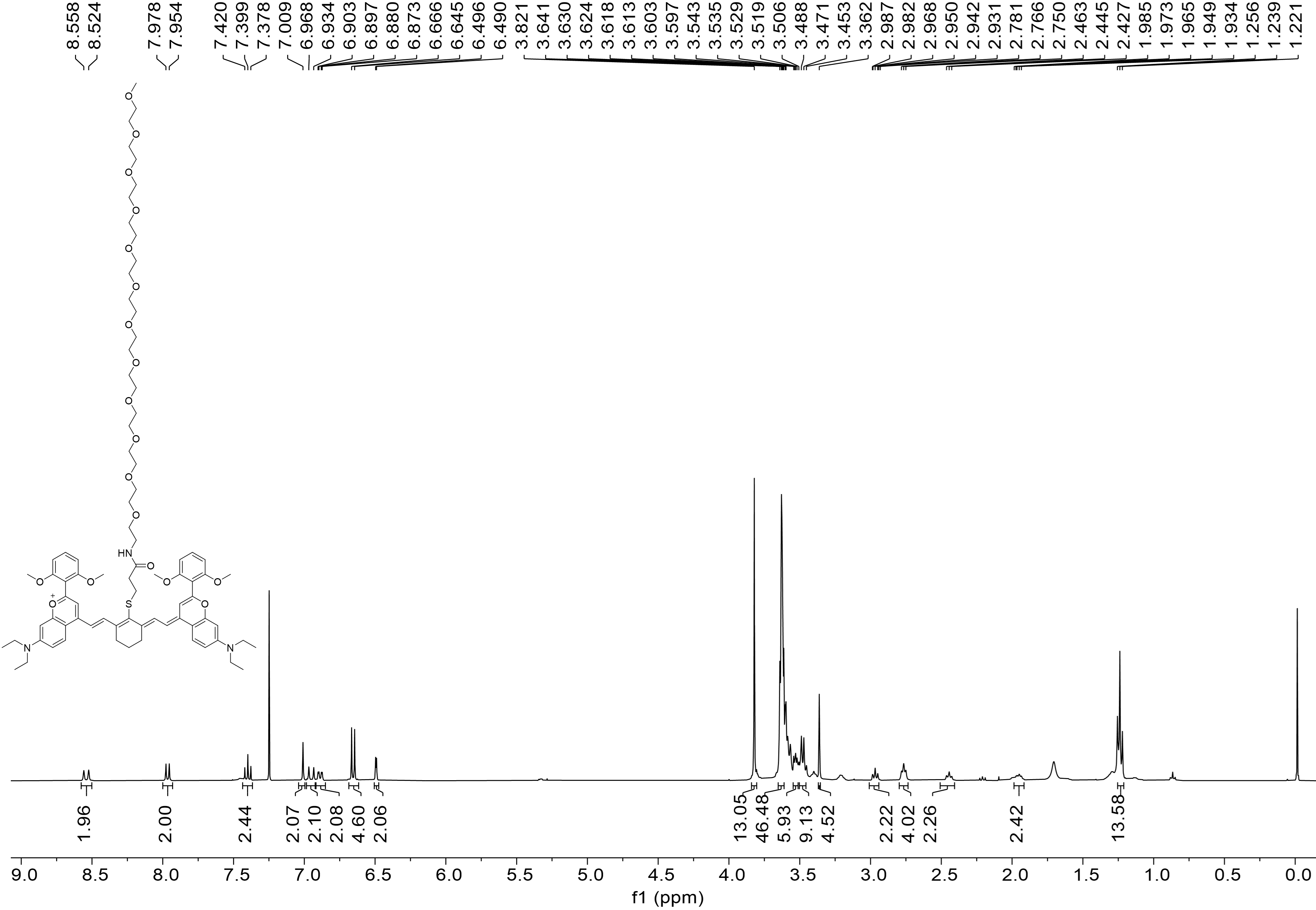


Chromatogram of Flav12


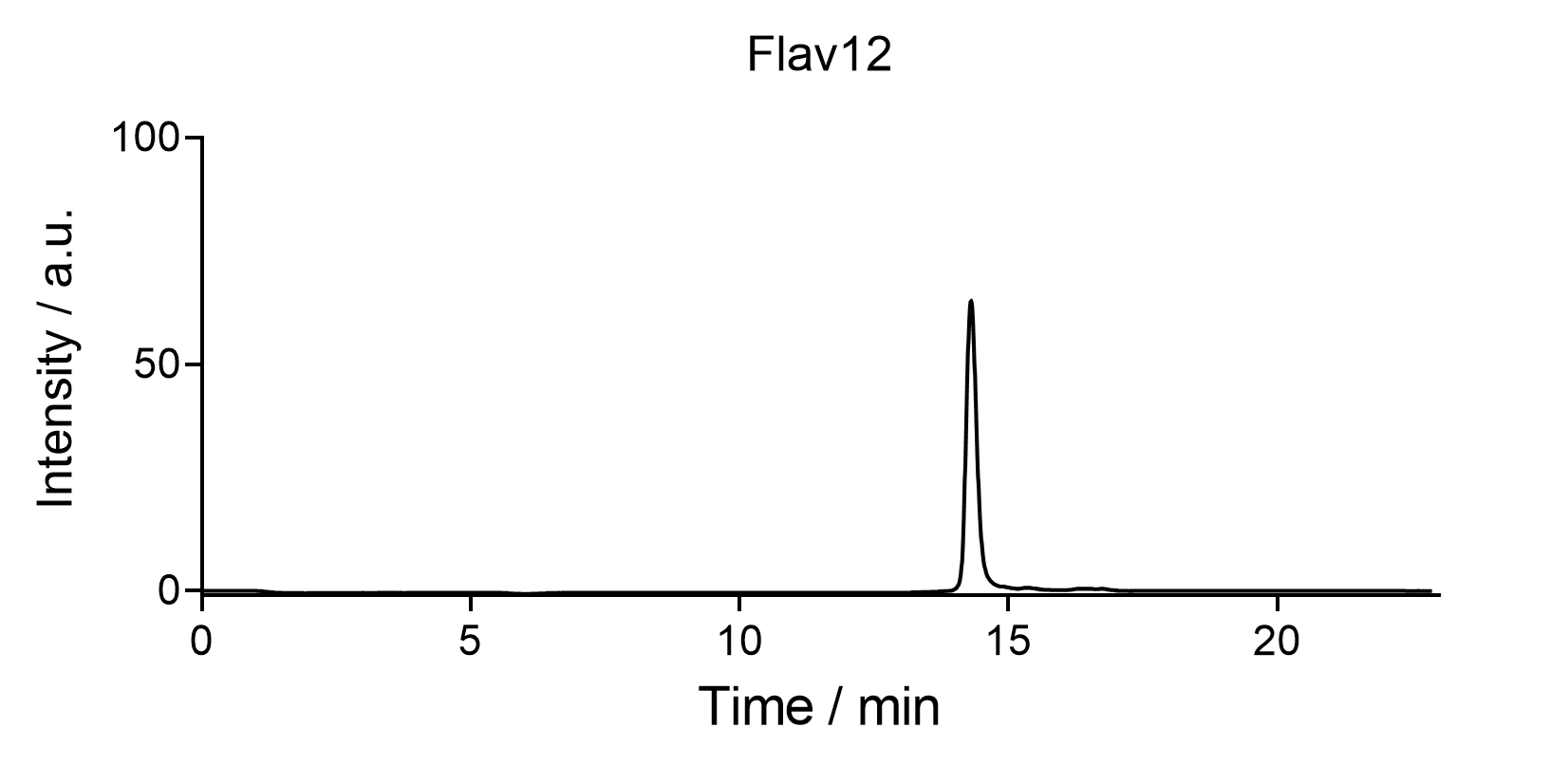


MALDI-TOF-MS spectra of Flav12


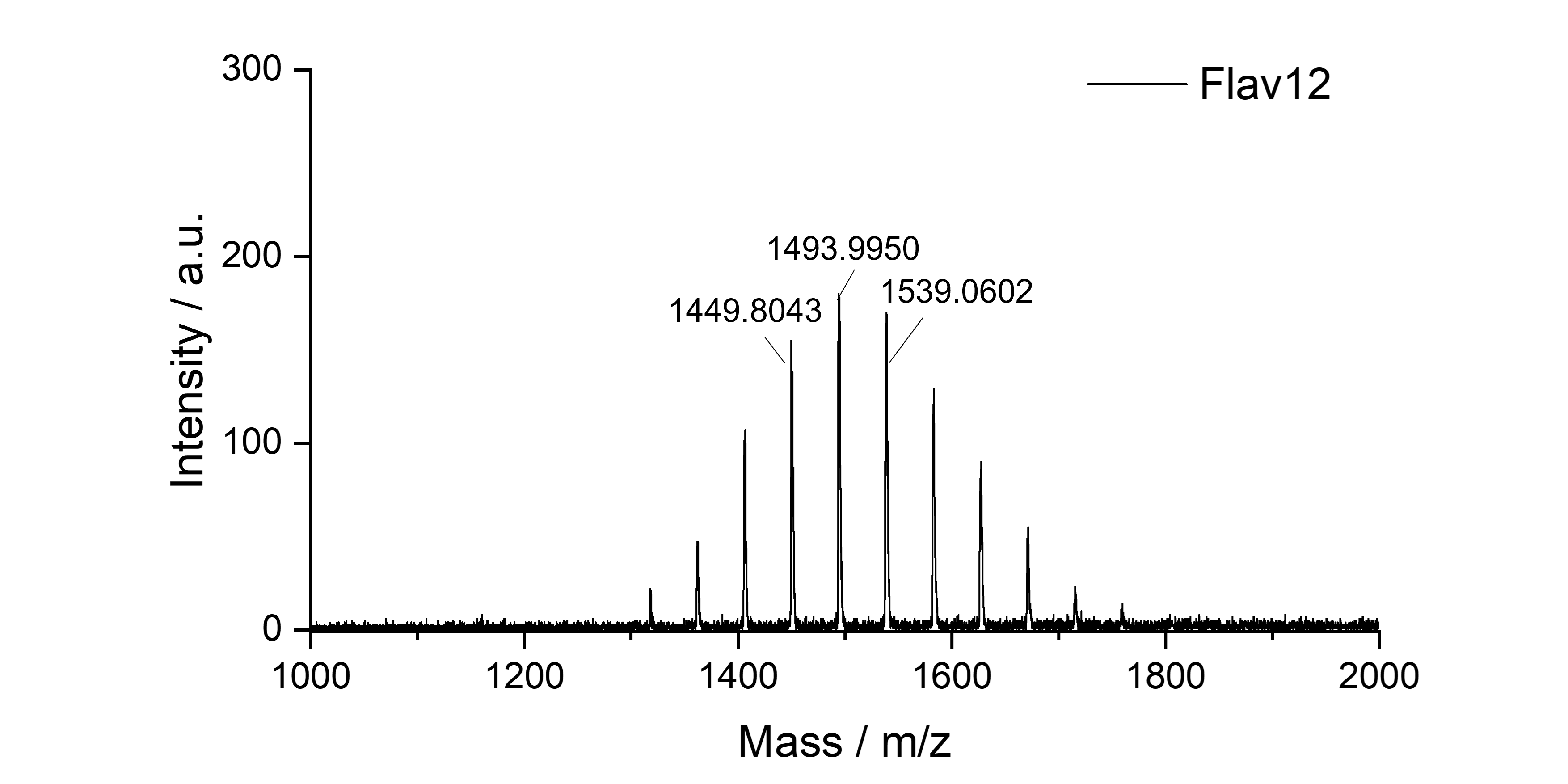


^1^H NMR spectra of Flav45


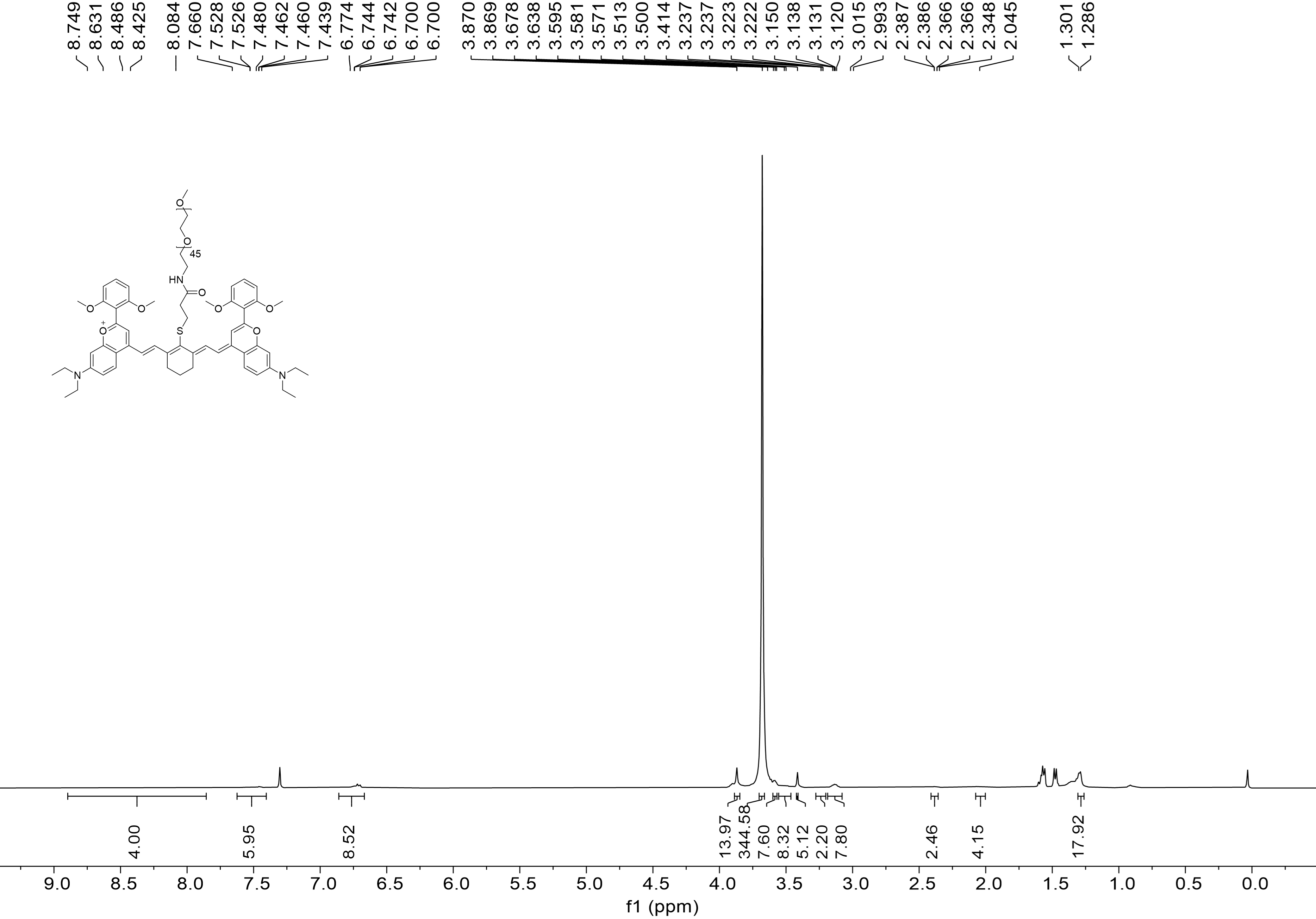


Chromatogram of Flav45


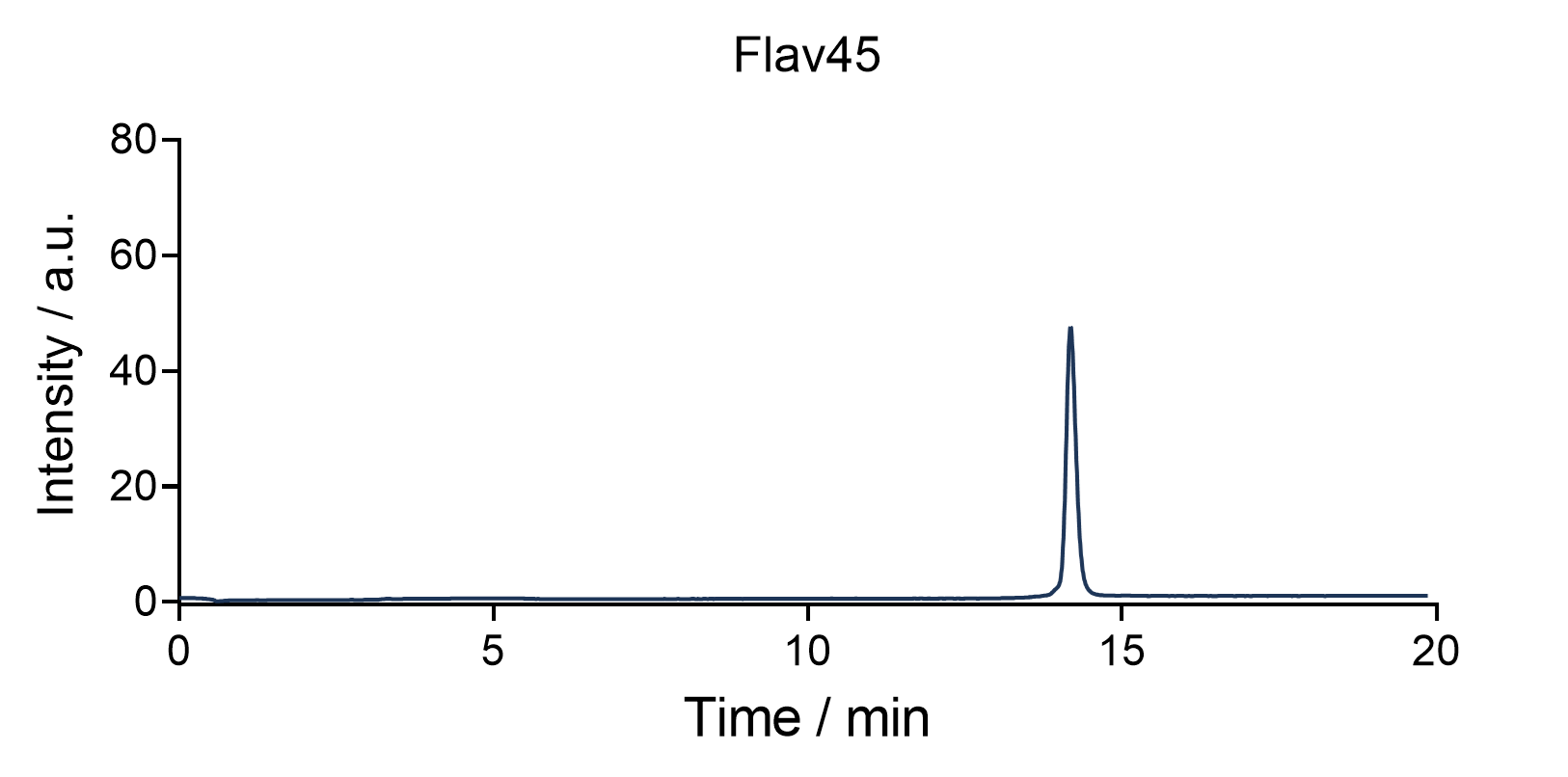


^13^C NMR spectra of Flav45


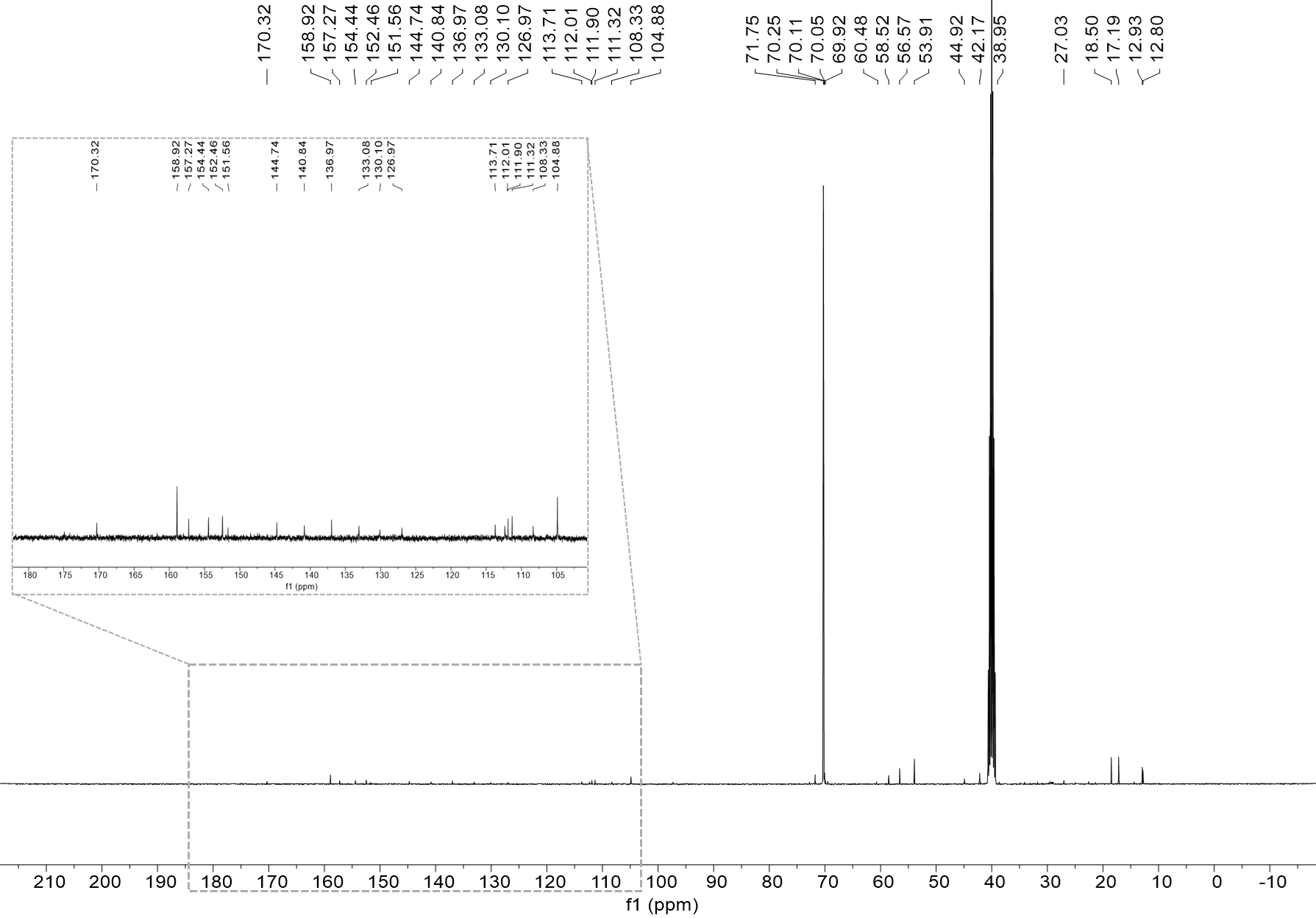


MALDI-TOF-MS spectra of Flav45


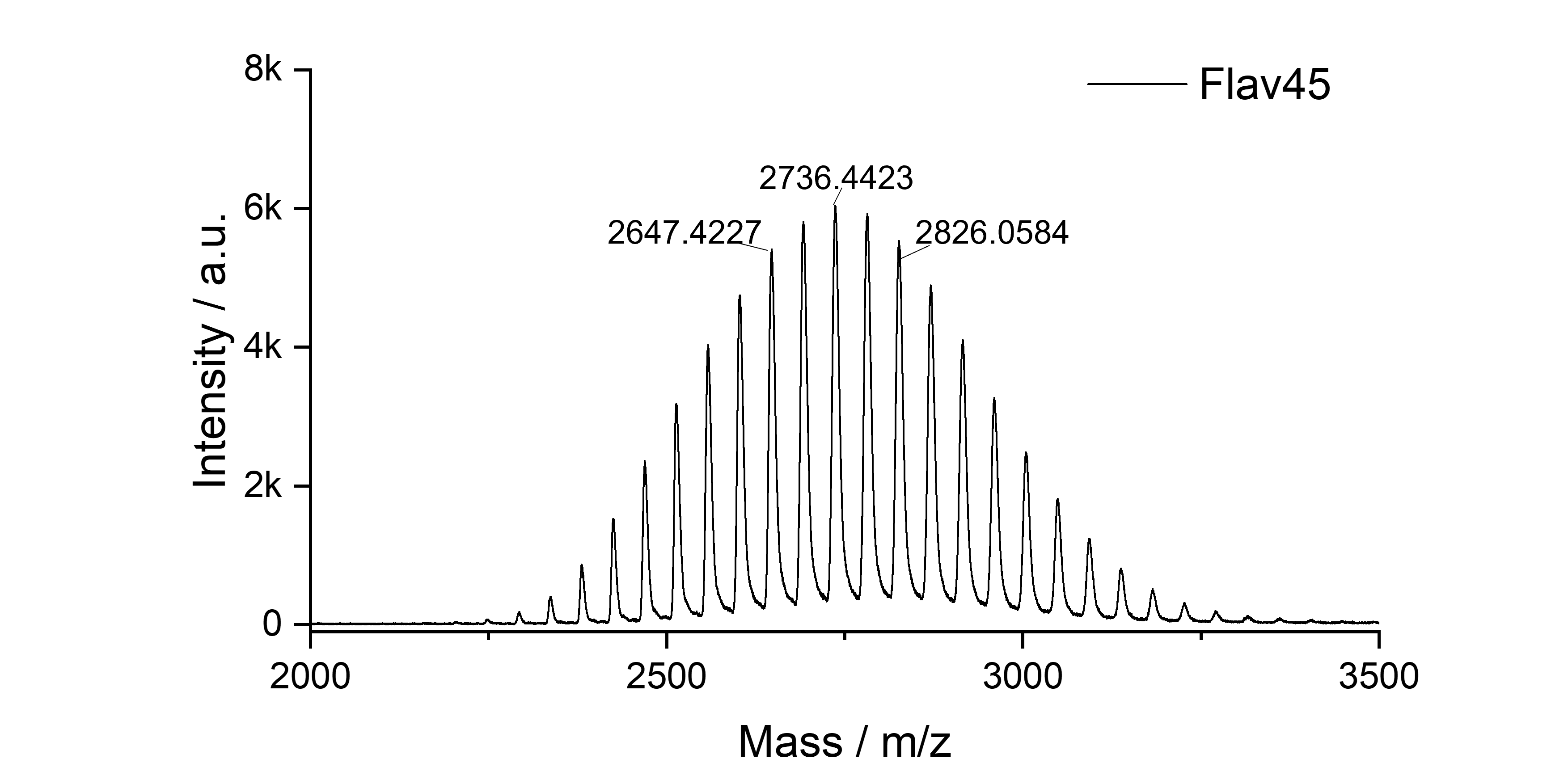


**References**

[1] L. Bai, Y. Jia, D. Ma, Y. Du, K. S. Hettie, Y. Zhang, S. Zhu, Chemogenic albumin-seeking NIR dyes for in vivo site-specific albumin tagging. *Sens. Actuators B Chem.* **2024**, 414, 135910.

[2] Z. Dang, X. Liu, Y. Du, Y. Wang, D. Zhou, Y. Zhang, S. Zhu, Ultra‐bright heptamethine dye clusters based on a self‐adaptive co‐assembly strategy for NIR‐IIb biomedical imaging. *Adv. Mater.* **2023**, 35, 2306773.

[3] H. Huang, W. Cruz, J. Chen, G. Zheng, Learning from biology: synthetic lipoproteins for drug delivery. *Wiley Interdiscip. Rev. Nanomed. Nanobiotechnol.* **2015**, 7, 298.
